# Ontogeny of dialects across chimpanzee vocalisations

**DOI:** 10.1371/journal.pone.0355313

**Published:** 2026-09-02

**Authors:** Kassandra Giragosian, Marina Davila-Ross, Derry Taylor

**Affiliations:** 1 Queen Mary University of London, London, United Kingdom; 2 University of Portsmouth, Portsmouth, United Kingdom; 3 Institute des Sciences Cognitives Marc Jeannerod, Lyon, France; University of Puerto Rico Medical Sciences Campus: Universidad de Puerto Rico Recinto de Ciencias Medicas, UNITED STATES OF AMERICA

## Abstract

Dialects are a vocal signature of population membership. Early in human ontogeny, vocal behaviour is relatively similar across populations from an acoustic perspective. As ontogeny progresses, the difference between populations becomes larger. While research in recent decades has shown chimpanzees show population-specific vocal signatures, debates regarding the sources of this variation, namely whether it results from social, genetic, or ecological factors, continue. By studying the ontogeny of vocal dialects, we can gain new insights into the processes generating cross-population variation in vocal acoustics. In this study, we measured the acoustic characteristics of infant (N = 21) and juvenile (N = 10) chimpanzee (*Pan troglodytes schweinfurthii*) laughs (N = 4733), grunts (N = 3694), and whimpers (N = 2798) at Chimfunshi Wildlife Orphanage and Gombe National Park. Using permuted discriminant function analysis, we found that we could discriminate between sites for all call types, indicating the presence of dialects across the chimpanzee vocal repertoire. This pattern held for juveniles, but infant whimpers did not show site-specific vocal signatures. Furthermore, we could discriminate between sites significantly better for juveniles compared to infants. We discuss the different processes through which the presence of population-specific vocal signatures across the vocal repertoire from infancy may emerge and argue that the development of increasingly recognisable vocal dialects in ontogeny suggests a role for social and ecological factors in shaping chimpanzee vocal acoustics.

## Introduction

Human sociality has a uniquely multi-level structure, with networks of friendships collectively making up communities, which in turn together make up cultural groups, countries, and even continents of people with shared identities [1].Within this multi-level structure humans co-operate and compete with one another [2]. Yet, even within a community, we often encounter individuals who are unknown to us. This poses an interesting question – how do we decide who to co-operate with? It has been argued that humans solve this problem using linguistic tags such as dialects, which are population-specific pronunciations that can be used to discriminate in-group from out-group members [3,4]. Human vocal communication is correspondingly extremely versatile from an acoustic perspective, encoding information about dynamic states such as emotion and arousal, as well as static information, such as identity at the individual and group level [5]. Early in human vocal development, within the first year, vocalisations are more acoustically similar across populations relative to later in development [6]. While this is clearly true of speech-sounds, as demonstrated by extensive cross-cultural variation in dialects, it has interestingly been shown also that non-verbal vocalisations show acoustic properties that mirror maternal language, even in neonatal infants [7]. The extent of cross-population variation in human vocal acoustics, however, varies greatly both between speech and non-speech, as well as within non-speech sounds, as laughter, for instance, seems to vary more acoustically across cultures than screams and cries, which seem to have more widely shared properties [8]. Altogether, this extensive variability in vocal acoustics indicates a significant process of shaping and learning throughout ontogeny which is little understood from an evolutionary perspective.

For nonhuman primates, cross-population variation in vocal acoustics has also been documented multiple times in recent decades (e.g., chimpanzees [9]; orangutans [10]; lemurs [11]; callitrichids [12]). Of particular relevance to human evolution, is the study of chimpanzees, our closest living relatives, who live in complex fission fusion societies with between-group conflicts, but no multi-level structuring with stable sub-units [1]. The first bioacoustic analysis confirming the presence of cross-population variability in call acoustics in chimpanzees came from a study of pant-hoot calls – a species-typical vocal sequence often used for long-distance communication [13]. In a cross-population study of pant-hoot call acoustics in Mahale and Gombe, significant differences in the acoustic characteristics of the ‘climax’ phase of pant-hoots were found [9]. Nonetheless, the authors remained agnostic about whether genetic, ecological, or social factors explained this variation. In a subsequent study of West African chimpanzees, it was found that neighboring communities living in the same habitat did indeed show community-specific vocal signatures which could also not be explained by genetics following DNA analysis of the sample populations [14]. The debate continues, however, since an analysis comparing East African chimpanzees at Kibale and Gombe found that apparent population-specific vocal signatures could instead be explained by identity encoding at the individual level [15].

Pant-hoot calls are used over long-distances and hence may be under pressure for encoding multiple types of information that are important when receivers are not visible, such as identity at the individual and group/population level [15,16]. Furthermore, long-distance calls may be under greater pressure to be modified for transmission in environments that are more or less conductive to sound propagation, such as dense versus more open forests [9]. Indeed, speech and laughter, which show the greatest cross-cultural variation in humans [8], are not long-distance forms of communication, calling into question the comparability between apparent chimpanzee and human dialects. However, in a unique translocation study on captive chimpanzees, it was found that different chimpanzee groups have an acoustically distinct grunt, a short-range food call. Fascinatingly, the groups converged acoustically after social integration [17]. Increasingly, it seems, chimpanzees do indeed show cross-population variability in vocal acoustics that points to the role of social processing in shaping call structure. Studies of chimpanzee laughter in sanctuary settings which have shown that more recently established colonies show different temporal communication patterns than older colonies further point towards social-shaping (e.g., [18]). All in all, there is notable evidence for group and population (i.e., neighboring groups and geographically separated populations) level vocal signatures in chimpanzees, but whether they develop early and become greater throughout ontogeny, like in humans, is currently unknown.

In fact, very little is known about the early ontogeny of chimpanzee vocal acoustics in general and there may be multiple explanations for it (e.g., infant vocalizations are often soft and difficult to record; mothers with infants often keep a distance from researchers and their equipment; the infant age class often has the least number of representatives within a chimpanzee colony). To our knowledge, only two studies to date have studied early infant chimpanzee vocal behaviour from a bioacoustic perspective. The first study was conducted on three-year-old chimpanzees, where whimpers and screams (close to mid-range distress calls used typically by young nonhuman primates) were found to show individual identity based on their spectral features [19]. The authors provided an explanation of the adaptive function, arguing that early vocal signatures are fundamental for the infants when they are no longer visible to their mothers and other kin. More recently, in the first systematic study of chimpanzee vocal ontogeny from an acoustic perspective throughout the repertoire it was found that acoustic gradation between calls significantly increases throughout ontogeny which theoretically can increase the encoding potential of chimpanzee calls [20]. However, whether young chimpanzees show population-level vocal signature, and whether between-population differences become greater throughout ontogeny as they do in humans in currently unknown. This gap in the literature is key to understanding the factors that shape cross-population call acoustics in chimpanzees and the evolutionary origins of the human vocal flexibility that facilitates complex sociality.

In the present study, our goal was therefore to fill this important gap in the nonhuman primate acoustics literature by combining developmental and cross-cultural perspectives. Specifically, we studied 30 chimpanzees across early developmental stages (infants and juveniles) and compared their vocal features across two populations in Africa (one population was from Chimfunshi Wildlife Orphanage in Zambia and the other one from Gombe National Park in Tanzania). We examined the most common call types produced by young chimpanzees (grunts, whimpers, and laughs). We hypothesised that if calls show population-level signatures, these signatures will be stronger encodings of population-identity later in ontogeny as is observed in humans. Since speech-related (e.g., grunt-like vocalisations; [21]) and positively valenced non-speech vocalisations such as laughter also show greater cross-population variability than negatively valenced non-speech sounds, such as screams and cries in humans [8], we expected that population-level signatures may be stronger for grunts and laughs compared to whimpers. If these hypotheses are supported, this would indicate the chimpanzee vocal repertoire structure is shaped by both widely shared biological constraints which operate in tandem with local social and/or ecological factors. This would suggest the foundations of the unparalleled versatility of human vocal structure are rooted in our non-human primate ancestry.

## Methods

### Ethics statement

All data collection was permitted by the University of Portsmouth, Animal Welfare and Ethical Review body (AWERB) and Chimfunshi Research Advisory Board (CRAB). This manuscript also meets the ASP ethical requirements.

### Inclusivity in global research

Additional information regarding the ethical, cultural, and scientific considerations specific to inclusivity in global research is included in the Supporting Information (S11 Checklist).

### Subjects

Subjects were 30 individual Chimpanzees. The subjects came from two populations: Chimfunshi Wildlife Orphanage (n = 17) and Gombe National Park (n = 13). Chimfunshi chimpanzees are semi-wild chimpanzees living in Miombo woodland. Gombe Chimpanzees are wild, living in semi-deciduous tropical rainforest. We focused on infant (N = 21) and juvenile (N = 10) subjects (one individual was recorded across both developmental stages). Twelve infants were from Chimfunshi and 9 were from Gombe. Five juveniles were from Chimfunshi and 5 were from Gombe. Infant ages ranged from 0–4 (*M*  =  1.25  ±  *SD*  =  1.29). Juveniles were aged >4–10 (*M*  =  6.41  ±  *SD*  =  1.06; S1 Table).

The infants comprised 9 females and 12 males, whereas the juveniles comprised 3 females and 7 males. An individual's status as an infant or juvenile was defined based on age alone.

### Data collection

A total of 1136 recordings were collected at Gombe, and 555 recordings were collected at Chimfunshi. At Chimfunshi, audio recordings were collected between 7 a.m. and 6 p.m. between April 2018 and August 2018 (excluding 12 p.m.–1 p.m. when daily feeding occurred) using a Sennheiser ME66 directional microphone which has a response frequency of 40 Hz – 20 kHz. Recordings were collected only when the subjects were outdoors, and the recordist was within 2–10 m of the subject. The main approach at Chimfunshi in collecting recordings was to use a 5-min focal sampling method in a randomized order each day that allowed us to have an equal representation of the sample in this study. However, due to the large size of the enclosures and the dense forest inside them, subjects were often not visible. For efficiency, we therefore decided to wait for 5 min to observe a subject. If the subject was not visible, we then recorded the visible subject who was next highest on the list. It was attempted to obtain two focal recordings on a subject in a single day—one in the morning (before 12 p.m.) and one in the afternoon (after 1 p.m.). Some of these recordings were incomplete as the subjects left the view of the camera for more than 30 s and could therefore no longer be seen and identified as the potential caller. Furthermore, when there were no visible subjects where 5-min focal recordings could be taken for that day, the subjects were also recorded ad libitum. For further information on the study population and data collection at Chimfunshi, see [20].

Sound recordings at Gombe were collected in the Kakombe Valley's feeding area. A Sennheiser MKH 815T directional microphone with a windscreen, which has a response frequency of 40 Hz – 20 kHz, and was connected to a portable Nagra recorder (mono, 19.05 cm/s), was pointed at the chimpanzees within a 5–15-meter range. The recordings included both chimpanzee vocalisations and spoken notes from the recordist, which identified the animals, their calls, and related behaviours. After recording, analogue audio samples of chimpanzee vocalisations were chosen from the tapes and linked to metadata, including Dutch transcriptions of the verbal commentary. These samples were created by cutting out relevant tape sections and joining them together, resulting in 28 reels with a total of 10 hours of chimpanzee sounds. Twenty reels focused on 17 young chimpanzees (one or more reels each), and 8 reels covered adults. In 2010, these analogue samples and the tapes were digitised and given to the Macaulay Library. The Dutch transcriptions were translated to English and, along with the metadata, were entered into spreadsheets (per individual) and then into the Macaulay Library database. For further information on the study population and data collection at Gombe [22]. Both sets of recording were digitised at the same sampling rate (e.g., 48 kHz with 16-bit accuracy).

### Identifying calls

We focused only on grunts, whimpers, and laughs (see Table 1 below for definitions), which have been demonstrated to be the most common and consistently used call types in the infant and juvenile periods [20,23,24]. Our dataset comprised 11225 units (Chimfunshi, N = 1087; Gombe, N = 10138). This comprised 3694 grunts (Chimfunshi, N = 300; Gombe, N = 3394), 4733 laughs (Chimfunshi, N = 210; Gombe, N = 4523) and 2798 whimpers (Chimfunshi, N = 577; Gombe, N = 2221).

**Table 1:** Definitions of call types based on previous studies of infant and adult chimpanzee vocalisations [20].

Call type	Definition	References
Grunt	Short, low-frequency calls given singularly or in short bouts. They may be tonal or noisy and produced with variable rhythm.	Kojima (2008) [23], Plooij (1984) [24] and Slocombe & Zuberbühler (2010) [13]
Whimper	Soft low-frequency tonal calls that can become higher in both frequency and amplitude as a bout progresses. Occasionally, they may contain harmonics.	Kojima (2008) [23], Plooij (1984) [24] and Slocombe & Zuberbühler (2010) [13]
Laughs	Staccato, noisy, low-frequency, alternating ingressive–egressive breathing patterns delivered in an irregular rhythm. Acoustic energy is audibly present in both ingression and egression, with most energy visible during ingresses. While some adopt a more comprehensive laughter definition that includes grunt-like sounds (e.g., Davila-Ross et al., 2009), we decided to adopt a narrower definition with minimal overlap with other call types given the aim of this study.	Plooij (1984) [24] and Slocombe & Zuberbühler (2010) [13]

### Acoustic analysis

Calls consist of either a single call element or a series of call elements, referred to as a “call bout.” A call was considered independent from a preceding call if it either occurred more than 5 seconds after the offset of the previous call or if there was a change in call type within a call bout.

Spectral and temporal features (Table 2) from each call element within each recording were extracted using the bioacoustics analysis program Raven Pro V1.6 [25]. The specific set of parameters was based on a previous study of chimpanzee vocal ontogeny on the same population, where this set of parameters was found to encode developmental shifts [20]. In Raven, spectrograms were generated using a fast Fourier transform. Since the majority of extracted measurements were spectral sound characteristics rather than temporal, narrowband spectrograms were chosen. A band-pass filter was applied to the spectrograms, ranging from 50 to 20,000 Hz. This bandwidth represented the range of frequencies where energy was visible in previous studies [26,27]. The sampling rate was 48,000 Hz with 16-bit accuracy.

**Table 2:** Acoustic parameters extracted and used in analysis with their definition.

Parameter	Definition
Total elements in bout	The total number of acoustic elements in a call.
Bout duration (s)	The amount of time between the onset and offset of a call.
Element rate (s^-1^)	The number of call elements in a call divided by the duration of that call.
Max Entropy (Bits)	The maximum entropy calculated for a spectrogram slice within the selection bounds.
Average Entropy (Bits)	The amount of disorder for a typical spectrum within the selection.
Average Power Density (db FS)	The typical power level of the signal within a specific frequency range.
Delta frequency (Hz)	The highest frequency (the highest frequency at which energy was detected in the call) minus the lowest frequency, corresponding to bandwidth.
Frequency 5% (Hz)	The frequency below which 5% of the total energy in the selection was found.
Q3 frequency (Hz)	The frequency below which 75% of the total energy in the selection was found.
Maximum frequency (Hz)	The frequency at which the maximum energy occurs in the call.
50% bandwidth (Hz)	The range of frequencies within which 50% of the total energy in the selection was found.
90% bandwidth (Hz)	The range of frequencies within which 90% of the total energy in the selection was found.
Peak frequency contour maximum frequency (Hz)	The highest frequency reached by the most prominent frequency component in a sound.
Peak frequency contour max slope (Hz/ms)	The steepest rate of change in frequency of the most prominent frequency component in a sound.
Peak frequency contour number of inflection points	How many times the *dominant* frequency of a sound changes direction

Call elements were manually selected by highlighting the lowest frequency where there was observable acoustic energy, the highest frequency where there was observable energy, the onset of the call, and the offset of the call. We aimed to highlight the full call element in a single selection. A Hanning window function was applied to call selections, which is the most appropriate window function for biological signal analysis because it prevents variation in onset–offset sound characteristics from introducing mathematical artefacts into the acoustic measurements [28]. When ambient sound from other animals overlapped with call elements of interest, we did not include these features in the selection.

To assess multicollinearity among acoustic variables, pairwise Pearson correlation coefficients were calculated for all acoustic parameters. Highly correlated variables were identified using the findCorrelation() function in the R package caret [29] with a threshold of r > 0.90. This algorithm iteratively removes the variable with the largest mean absolute correlation among highly correlated variables, thereby reducing redundancy while retaining representative descriptors. Since spectral and temporal acoustic features are under different selection pressures according to theory in evolutionary ecology (see [30]), we conducted this procedure for spectral and temporal acoustic variables separately, thereby allowing us to evaluate the contribution of both sets of parameters to population discrimination. Full correlation matrices for spectral variables before and after this procedure are shown in S1 and S2 Figs respectively. Only one correlation matrix is shown for temporal parameters (S3 Fig), since no temporal variables were removed because there were no correlations between variables that exceeded our threshold of 0.9. Finally, we had 15 acoustic parameters – 3 temporal and 12 spectral (See Table 2 for the names of retained parameters and their definitions).

### Statistical analysis

To investigate whether each chimpanzee vocalisation type (laughs, grunts, whimpers) could be discriminated between the two populations (Chimfunshi vs Gombe), we performed pDFA (Discriminant Function Analysis combined with a permutation test) in R version 4.4. using a function (provided by R. Mundry) which is based on the function *lda* of the R package *MASS* [31]. As explained by R. Mundry [32], standard DFA can lead to inflated results (due to pseudoreplication) when dealing with non-independent data, such as multiple calls from the same individual. The pDFA addresses this issue. It works by comparing the average correct classification rate (effect size) from 100 DFA iterations on the real data to the distribution of correct classification rates obtained from 1000 datasets where population sites were randomly assigned. This randomisation helps determine statistical significance. The effect size itself is calculated as the average percentage of correctly classified sounds across the 100 validation datasets in the cross-validation step. This percentage is then compared to chance, calculated as the average number of correct classifications across 1000 randomized permutations of call labels. Finally, we calculated the proportion of randomised datasets that yielded a correct classification rate at least as high as our observed effect size. This proportion indicated the significance of the discrimination level [32].

Two sets of pDFA models were conducted. The first set comprised three pDFA models, which examined discrimination for each vocalisation type between the two populations, controlling for individual identity (i.e., holding identity constant while randomizing group membership as per [32], ruling out explanation of difference based on individual identity, see [15]). This set of models included all 15 acoustic measurements. The second set comprised 6 pDFA models, which investigated discrimination within each vocalisation type between the two populations separately for infants and juveniles, controlling for individual identity as above. This set of models excluded the peak frequency contour number of inflection points as the models would not converge with this acoustic parameter included. From our pDFA models we extracted factor loadings to understand which acoustic variables played a role in discriminating between populations. Since the pDFA takes an iterative approach, we calculated the mean, standard deviation, median, and 95% confidence intervals of factor loadings, as well as the proportion of iterations in which each acoustic variable generated the same sign (i.e., + or – which indicated the direction of discrimination towards one population or the other and was held constant, specifically with positive values indicating Gombe and negative values indicating Chimfunshi).

Finally, to test whether population-level classification accuracy differed between infants and juveniles across development, we modelled correct classification rates using beta regression, which is appropriate for proportional response variables bounded between 0 and 1. Models were fitted in R using the *glmmTMB* package [33] with a logit link function. Model fit was assessed via simulated model residuals using the *DHARMa* package [34]. For each call type (grunts, whimpers, laughs), the classification rate was entered as the response variable and age group (infant vs. juvenile) as a fixed predictor. We did not use age as a continuous variable owing to an insufficient number of individuals at some ages (S1 Table). To account for the non-independence of calls produced within the same vocal bout, bout ID was included as a random intercept. The final model formula was therefore *class_rate_beta ~ age_group + (1 | id_bout).* We did not include individual ID as a random factor because, as mentioned above in our explanation of our pDFA procedure, we controlled for individual ID at that stage by holding ID constant during the permutation process, which is standard procedure when controlling for the influence of grouping factors where multiple data points come from the same group (see 33). Significance of fixed effects was assessed using Wald z-tests, and predicted marginal means with 95% confidence intervals were obtained from model estimates on the response scale.

## Results

We found significant discrimination between populations for the three call types examined. Specifically, whimpers exhibited strong population discrimination (p = 0.001), achieving 97.62% correctly cross-classified cases compared to an expected average of 69.91%, followed by laughs (p = 0.001; correctly cross-classified cases = 96.83%, expected average = 60.04%) and grunts (p = 0.001, correctly cross-classified cases = 89.67%, expected average = 60.04%). Descriptive statistics of call acoustics for each call type, per developmental stage and site are given in S2-S4 Tables.

### Infant calls

#### Grunts.

For infant grunts, population discrimination was significant (p = 0.002, correctly classified = 78.25%, expected = 52.02%). Factor loadings for infant grunts showed considerable variability across permutations, with wide confidence intervals for the highest-loading variables. Average entropy exhibited the largest negative mean loading (mean = −8.28, 95% CI: −35.24 to 2.11) and the highest directional consistency (89% of permutations with the same sign), suggesting it was the most stable contributor to population discrimination despite substantial variation in effect size. Maximum entropy also showed a relatively large positive loading (mean = 6.52, 95% CI: −4.94 to 27.69) with moderate sign consistency (76%), whereas element rate displayed a small positive mean loading (0.66, 95% CI: −21.97 to 29.59) and low sign consistency (52%), indicating little stability across permutations. Bout duration (mean = −0.41, 95% CI: −7.09 to 4.50; 61% same sign), total elements in bout (mean = 0.32, 95% CI: −2.32 to 3.54; 66% same sign), and average power density (mean = −0.07, 95% CI: −0.73 to 0.94; 66% same sign) all showed comparatively small loadings with confidence intervals overlapping zero. All remaining spectral and frequency-related variables had mean loadings close to zero and approximately chance-level sign consistency, indicating minimal contributions to discrimination. See S3 Table for descriptive statistics on acoustic variables and S5 Table for factor loadings.

Overall, population differentiation in infant grunts was primarily associated with entropy measures, particularly average entropy, although no acoustic parameter showed a confidence interval excluding zero.

#### Whimpers.

For infant whimpers, population discrimination was not significant (p = 0.123, correctly classified = 71.81%, expected = 55.31%). Despite the lack of significant discrimination, several variables showed relatively consistent directional effects. Average entropy exhibited the largest mean loading (mean = −7.41, 95% CI: −23.57 to 0.16) and the highest sign consistency (96%). Maximum entropy also showed a relatively large positive loading (mean = 4.60, 95% CI: −3.56 to 24.26) with high sign consistency (86%). In contrast, element rate showed substantial variability (mean = −0.95, 95% CI: −26.51 to 31.27; 53% same sign), while bout duration (mean = −0.28, 95% CI: −5.06 to 4.44; 58% same sign), total elements in bout (mean = 0.10, 95% CI: −1.04 to 1.60; 59% same sign), and average power density (mean = −0.05, 95% CI: −0.93 to 1.26; 64% same sign) showed relatively small and inconsistent effects. All remaining frequency and spectral variables had mean loadings close to zero with confidence intervals overlapping zero. See S2 Table for descriptive statistics on acoustic variables and S6 Table for factor loadings.

Overall, although average and maximum entropy displayed relatively consistent directional effects, no acoustic variable exhibited a stable loading sufficient to produce significant population discrimination.

#### Laughs.

For infant laughs, population discrimination was significant (p = 0.006, correctly classified = 93.17%, expected = 59.31%). Element rate showed the strongest contribution to population discrimination (mean = 1.91, 95% CI: −0.26 to 4.49) with high sign consistency (93%), although its confidence interval overlapped zero. Average entropy (mean = 0.37, 95% CI: −2.33 to 2.88; 64% same sign) and maximum entropy (mean = −0.11, 95% CI: −2.03 to 2.13; 57% same sign) contributed relatively little. Average power density exhibited a very small mean loading (0.02, 95% CI: −0.30 to 0.32) and low sign consistency (54%). Bout duration and total elements in bout also showed negligible effects, and all remaining spectral and frequency-related variables had mean loadings effectively equal to zero. See S4 Table for descriptive statistics on acoustic variables and S7 Table for factor loadings.

Overall, discrimination among infant laughs was driven primarily by variation in element rate, while all other acoustic parameters contributed only weakly and inconsistently.

### Juvenile calls

#### Grunts.

For juvenile grunts, population discrimination was significant (p = 0.022, correctly classified = 90.58%, expected = 56.77%). Element rate showed the largest mean loading (mean = 3.76, 95% CI: −0.28 to 13.41) together with high sign consistency (94%), indicating it was the dominant contributor to population discrimination despite confidence intervals overlapping zero. Average entropy (mean = −0.22, 95% CI: −1.55 to 1.23; 65% same sign), maximum entropy (mean = −0.13, 95% CI: −1.29 to 0.85; 56% same sign), and average power density (mean = −0.06, 95% CI: −0.35 to 0.27; 77% same sign) showed comparatively small effects. Bout duration, total elements in bout, and all remaining frequency-related variables had mean loadings close to zero with confidence intervals overlapping zero. See S3 Table for descriptive statistics on acoustic variables and S8 Table for factor loadings.

Overall, juvenile grunt discrimination was driven predominantly by temporal patterning through element rate, whereas all other acoustic variables contributed minimally.

#### Whimpers.

For juvenile whimpers, population discrimination was significant (p = 0.004, correctly classified = 87.89%, expected = 56.97%). Average entropy showed the largest negative mean loading (mean = −1.77, 95% CI: −9.10 to 2.21) and relatively high sign consistency (78%), while maximum entropy showed a positive loading (mean = 0.98, 95% CI: −4.38 to 6.79) with 71% sign consistency. Element rate had the largest absolute loading (mean = 1.80, 95% CI: −12.02 to 20.18) but lower sign consistency (58%), indicating substantial variability across permutations. Average power density contributed little (mean = 0.01, 95% CI: −0.58 to 0.47; 54% same sign), as did total elements in bout and bout duration. All remaining spectral and frequency-related variables had mean loadings near zero and confidence intervals overlapping zero. See S2 Table for descriptive statistics on acoustic variables and S9 Table for factor loadings.

Overall, juvenile whimper discrimination showed weak associations with entropy measures, but no acoustic variable demonstrated a consistently strong contribution across permutations.

#### Laughs.

For juvenile laughs, population discrimination was significant (p = 0.019, correctly classified = 96.70%, expected = 66.27%). Element rate showed the largest absolute loading (mean = −3.22, 95% CI: −7.78 to 2.18) with relatively high sign consistency (81%), suggesting temporal patterning contributed most strongly to discrimination despite confidence intervals overlapping zero. Maximum entropy (mean = 0.29, 95% CI: −0.90 to 1.52; 67% same sign), average entropy (mean = 0.19, 95% CI: −2.11 to 1.63; 67% same sign), and average power density (mean = 0.08, 95% CI: −0.20 to 0.21; 75% same sign) all showed comparatively small effects. Bout duration and total elements in bout likewise exhibited negligible loadings, and all remaining spectral variables were centred near zero. See S4 Table for descriptive statistics on acoustic variables and S10 Table for factor loadings.

Overall, juvenile laugh discrimination was primarily associated with element rate, whereas spectral and frequency-related variables showed only weak and inconsistent contributions.

### Development of dialects

Across all three call types, classification accuracy was significantly higher in juveniles than in infants.

#### Grunts.

For grunts (N = 3,694 calls), juveniles showed significantly higher classification rates than infants (β = 1.265 ± 0.063 SE, z = 20.10, p < 0.001). Predicted mean classification rates for infants were 0.761 (95% CI: 0.752–0.770) and 0.919 (95% CI: 0.910–0.927) for juveniles.

#### Whimpers.

For whimpers (N = 2,798 calls), classification accuracy was also higher in juveniles (β = 0.542 ± 0.105 SE, z = 5.19, p < 0.001). Predicted means were 0.766 (95% CI: 0.740–0.790) for infants and 0.849 (95% CI: 0.828–0.867) for juveniles.

#### Laughs.

For laughs (N = 4,733 calls), juveniles again showed higher classification accuracy than infants (β = 0.729 ± 0.108 SE, z = 6.73, p < 0.001). Predicted means were 0.929 (95% CI: 0.919–0.938) for infants and 0.965 (95% CI: 0.959–0.970) for juveniles.

**Fig 1:**
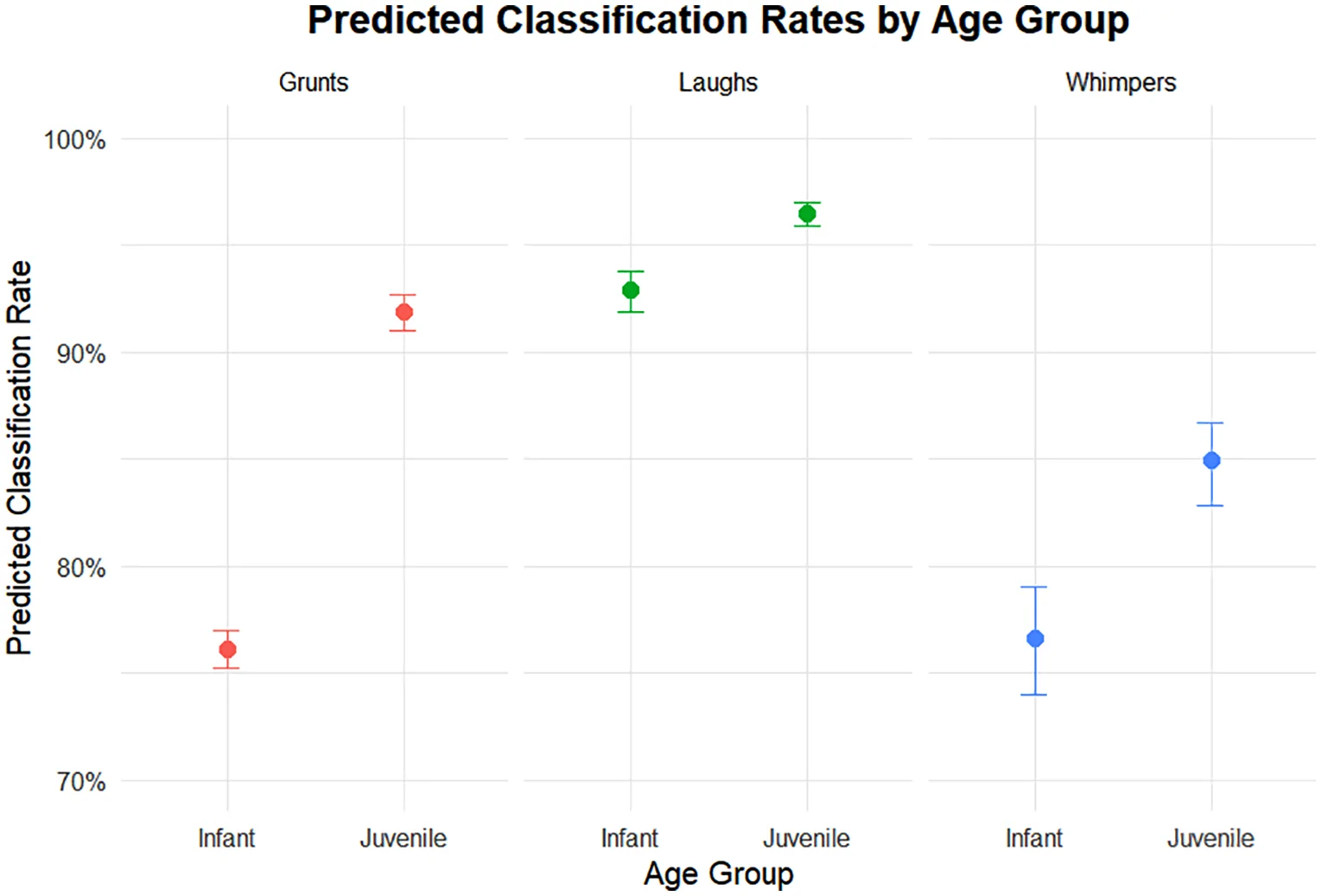
Comparison of vocalisation classification accuracy for each call type between the two age groups of infants and juveniles.

Overall, these results demonstrate a consistent developmental increase in population-level discriminability from infancy to the juvenile stage across all call types (Fig 1).

## Discussion

In the present study, we aimed to examine the ontogeny of vocal dialects in chimpanzees. We found that overall, all three of the studied call types encoded population-level identity. For grunts and laughter, we found this pattern for both infants and juveniles, consistent with our hypothesis that call types that are more speech-related (e.g., grunts; [21]) and positively grounded (e.g., laughs) [18] would show stronger signs of group identity, as is observed cross culturally in humans. For whimpers, we found it for juveniles only, consistent with our hypothesis that call types expressing negative affect are more constrained in their acoustic form across populations relative to other call types. Further, comparisons between infants and juveniles showed that classification accuracies of population were significantly higher for juveniles for all three call types. Our results suggest that population-level vocal signatures are present across the vocal repertoire, although the magnitude of variability may depend on vocal function. Furthermore, our observation of an increasingly strong signature of population-identity in vocal ontogeny suggests that vocal structure is shaped in ontogeny by non-genetic factors such as learning influenced by social interaction and or ecology.

Behavioural traits that appear early in ontogeny are often interpreted as being most likely innate [35]. One might interpret our finding of population-level identity in the grunts and laughs of infant chimpanzees as indications of a genetic explanation of cross-population variability in call structure. Indeed, this may be the case, since our populations have no between-population migration, making them most likely different in genetic composition despite being members of the same sub-species. However, we remain open to other interpretations also. In humans, it is known that even neonatal infants show variability in vocalisation acoustic structure depending on the ambient language environment [7], which may be due to either pre-natal effects, or, rapid impact of early social experience. We believe this possibility is certainly worth considering given that 1) our study species are the closest living relatives of humans who show signs of vocal learning at least as early as 9 months of age [36], and 2) most of our youngest infants in the sample are at least 6 months old and range up to 4 years of age. This, we believe, could be sufficient for vocal learning to occur, since studies of the ontogeny of chimpanzee vocal sequences have demonstrated rapid increases from ages 2–3 which continues until ages 8–9, which has been attributed to social learning processes [37].

Further evidence against a purely genetic explanation for the early acoustic variability observed in our sample is that population differences were not consistent across the entire vocal repertoire, but were restricted to grunts and laughs. Infant whimpers did not encode population identity. If genetic divergence between populations were the primary driver of the observed variation, we would not necessarily expect such call-type specificity. Instead, our findings suggest that different vocalisations may be subject to different developmental or selective pressures.

This interpretation is consistent with an increasing body of evidence for vocal plasticity in non-human primates. Experimental studies in common marmosets have demonstrated that social feedback plays a key role in shaping vocal development during infancy, supporting the idea that early vocal production is developmentally flexible rather than genetically fixed (e.g., [38–41]). Vocal plasticity during ontogeny has also been described in several other primate species (e.g., [42–45]), while population-specific vocal differences and evidence of vocal learning have also been reported in mouse lemurs ([46,47]). Together, these findings suggest that developmental plasticity and population-level acoustic variation may be more widespread among primates than previously recognised. This is particularly interesting with respect to recent studies that have suggested chimpanzee brains and vocal behaviour are much more precocial relative to co-operatively breeding species such as humans and marmosets [48].

Our results also parallel patterns reported in humans. Human speech exhibits clear population-specific acoustic variation (dialects), and even non-linguistic vocalisations such as laughter show cultural variation, whereas negatively valenced vocalisations such as screams and cries are considerably more conserved across cultures [8]. This pattern has been interpreted as reflecting different selective pressures acting on different classes of vocal signals, with distress-related calls maintaining a relatively conserved structure because of their importance for survival.

Although the absence of population differences in whimpers argues against genetics as the sole explanation for the observed acoustic variation, we cannot exclude an alternative evolutionary explanation. Because whimpers function as distress signals, genetic variants that substantially alter their acoustic structure could reduce the effectiveness of parent-offspring communication, potentially resulting in reduced maternal responsiveness and lower offspring survival. Such stronger stabilising selection on distress calls could therefore also contribute to their greater acoustic consistency across populations. Overall, we propose that chimpanzee vocal development is shaped by a combination of developmental plasticity and shared socio-environmental pressures, resulting in both repertoire-wide consistency and call-type-specific acoustic variability [49,50].

Although we observed population-level vocal signatures from early in ontogeny, at least for grunts and laughs, these signatures appeared to strengthen as development progressed. This pattern suggests that non-genetic factors contribute to shaping call structure during development, consistent with increasing vocal plasticity over ontogeny. While the specific acoustic features underlying population differentiation differed across call types, the observed developmental trajectories are compatible with the idea that vocal behaviour is progressively shaped by the social and ecological environment.

One potential source of such population-level variation is habitat. The two study populations inhabit acoustically distinct environments, with Gombe comprising dense tropical rainforest and Chimfunshi consisting predominantly of more open miombo woodland. Habitat-related differences in sound transmission may therefore contribute to divergence in vocal production or selection on acoustic features over development. Similar habitat-associated acoustic variation has been reported in other primates; for example, olive baboons produce grunts that differ in acoustic structure between open and closed habitats [51]. Although our study was not designed to test the acoustic adaptation hypothesis directly, the observed developmental increase in population-specific vocal signatures is consistent with the possibility that socio-environmental factors, including habitat, contribute to shaping chimpanzee vocal development. Interestingly, both spectral and temporal acoustic parameters were consistently ranked among the top contributors to population discrimination according to the factor loadings, suggesting multiple sources of selection may be shaping signal characteristics across populations.

At the same time, developmental changes in vocal anatomy and motor control likely also contribute to the observed strengthening of population discrimination. As chimpanzees mature, growth of the vocal tract [52] and improvements in respiratory and laryngeal control [53] may increase the stability and modulation capacity of calls, potentially amplifying population-level differences that are only weakly expressed in infancy. In addition, age-related differences in behavioural or recording context — such as infants being carried more frequently by their mothers and therefore vocalizing at closer range — could influence acoustic properties or signal transmission characteristics between age classes, although this is unlikely to account for the consistent population differences observed here. More broadly, developmental changes in the social contexts in which calls are produced may also contribute to the strengthening of population signatures. For example, laughs are predominantly produced during play and are most often directed toward peers, although juveniles may also engage in play with older or younger partners [54]. Increased peer interaction during the juvenile period may therefore provide opportunities for convergence through repeated social exposure or feedback within age cohorts. In contrast, whimpers are primarily directed toward mothers [50], and while systematic studies of developmental changes in their usage are limited, their predominantly dyadic context may constrain opportunities for broader social shaping, thereby explaining the lack of discriminability early in development. Grunts show a different developmental trajectory: although infants grunt toward a range of partners, call usage becomes increasingly socially specific with age, with juveniles — like adults — directing grunts preferentially toward higher-ranking individuals [55]. Such shifts in social targeting may alter both the frequency and diversity of models available for vocal exposure.

Taken together, these context-specific patterns raise the possibility that different mechanisms — including social feedback during play, convergence through repeated exposure, eavesdropping, or increasing sensitivity to socially salient partners — could contribute to the ontogenetic strengthening of population-specific vocal features. While our data do not allow us to distinguish among these mechanisms, incorporating fine-grained behavioural analyses alongside acoustic measures in future work will be essential for clarifying how social experience shapes developing vocal structure.

Before concluding, we would like to turn our attention to the key limitation of this study, namely, that it is a retrospective study. As such, the recordings used in this study were collected using different protocols and different equipment, although all recordings have been classified and measured using an identical protocol. We do not believe that this factor has played a role in our analysis. If differences between populations were primarily driven by recording apparatus or signal quality, we would expect systematic acoustic disparities across all call types and developmental stages, particularly in measures sensitive to recording fidelity such as entropy. However, average entropy values did not differ consistently between sites for all call types. This indicates that background noise or recording resolution differences are unlikely to explain the observed population-level patterns. Instead, we find that some call types encode population identity whereas others do not, and that the strength of this signature increases with ontogeny and varies between call types. We see no way in which the differences in equipment used could account for this structured, call type–specific pattern. Similarly, an imbalance in the amount of data per site cannot account for our findings, as this would also predict similar patterns across call types and age classes, and moreover, if the model simply predicted the majority class, we should expect no difference between expected average correct classification and correctly classified cross-validated cases. Yet, in almost every case, performance was significantly worse when site labels were randomized.

To conclude, in this study we aimed to assess whether there are signatures of population identity across the chimpanzee vocal repertoire, and how population identity signatures develop. We found that we could identify population-membership above chance levels for grunts and laughs, but not whimpers early in ontogeny, and classification accuracy significantly increased throughout ontogeny. We interpret our findings as suggesting that chimpanzee vocal structure at the repertoire level is likely shaped by local social and ecological environmental conditions throughout ontogeny. Our findings show a striking parallel with cross-population variability in speech and non-speech vocalisations, which implies deep evolutionary origins for human vocal versatility.

## Supporting information

S1 Table Overview of subject characteristics including developmental stage, age, sex, and population. (DOCX)

S2 Table Mean and standard deviation of each acoustic parameter included in the analysis for whimpers. (DOCX)

S3 Table Mean and standard deviation of each acoustic parameter included in the analysis for grunts. (DOCX)

S4 Table Mean and standard deviation of each acoustic parameter included in the analysis for laughs. (DOCX)

S5‍ Table Factor loading of infant grunt PDFA model. Positive values = Gombe. Negative values = Chimfunshi.
(DOCX)

S6‍ Table Factor loadings of infant whimper PDA model. Positive values = Gombe. Negative values = Chimfunshi.
(DOCX)

S7‍ Table Factor loading of infant laugh PDFA model. Positive values = Gombe. Negative values = Chimfunshi.
(DOCX)

S8‍ Table Factor loadings of juvenile grunt PDFA model. Positive values = Gombe. Negative values = Chimfunshi.
(DOCX)

S9 Table Factor loadings for juvenile whimper PDFA model. Positive values = Gombe. Negative values = Chimfunshi.
(DOCX)

S10 Table Factor loadings for juvenile laugh PDFA model. Positive values = Gombe. Negative values = Chimfunshi.
(DOCX)

S1 Checklist Inclusivity in global research questionnaire. (DOCX)

S1 Data Data used for analysis. (XLSX)

S1 R Script R script used for analysis. (DOCX)

S1 Fig Pearson correlation coefficient matrix between all originally measured spectral acoustic parameters. (DOCX)

S2 Fig Person correlation coefficient matrix between all remaining spectral acoustic parameters after removing for multi-collinearity. (DOCX)

S3 Fig Pearson correlation matrix between temporal variables showing no correlations between variables > 0.9. (DOCX)
