# Supplementary material for: Ontogeny of dialects across chimpanzee vocalisations
Source: PLoS One. 2026 Sep 2;21(9):e0355313. doi: 10.1371/journal.pone.0355313 (PMC13537595; doi:10.1371/journal.pone.0355313)
Supplement: Supplementary file 1 [file pone.0355313.s001.docx]

**S1 Table. Overview of subject characteristics including developmental stage, age (years) and population.**

| **Individual ID** | **Developmental Stage** | **Age (years)** | **Population** |
| --- | --- | --- | --- |
| Q | Infant | 0 | Chimfunshi |
| Y | Infant | 0 | Chimfunshi |
| CA | Infant | 0 | Chimfunshi |
| O | Infant | 0 | Chimfunshi |
| T | Infant | 0 | Chimfunshi |
| X | Infant | 1 | Chimfunshi |
| S | Infant | 2 | Chimfunshi |
| V | Infant | 2 | Chimfunshi |
| Z | Infant | 2 | Chimfunshi |
| W | Infant | 3 | Chimfunshi |
| AA | Infant | 3 | Chimfunshi |
| DA | Infant | 4 | Chimfunshi |
| U | Juvenile | 5 | Chimfunshi |
| N* | Juvenile | 5/6 | Chimfunshi |
| BA | Juvenile | 6 | Chimfunshi |
| R | Juvenile | 7 | Chimfunshi |
| P | Juvenile | 8 | Chimfunshi |
| I | Infant | 0 | Gombe |
| L | Infant | 0 | Gombe |
| A* | Infant | 0/1 | Gombe |
| H* | Infant | 0/1 | Gombe |
| M* | Infant | 0/2 | Gombe |
| D* | Infant | 0/2 | Gombe |
| G* | Infant | 1/2 | Gombe |
| J* | Infant | 2/3 | Gombe |
| K** | Infant-Juvenile | 3/5 | Gombe |
| F* | Juvenile | 5/7 | Gombe |
| E* | Juvenile | 6/7 | Gombe |
| B* | Juvenile | 6/8 | Gombe |
| C* | Juvenile | 7/8 | Gombe |

** Recorded at different ages (years) within the same developmental stage.*

*** Recorded at different developmental stages.*
