# Supplementary material for: Ontogeny of dialects across chimpanzee vocalisations
Source: PLoS One. 2026 Sep 2;21(9):e0355313. doi: 10.1371/journal.pone.0355313 (PMC13537595; doi:10.1371/journal.pone.0355313)
Supplement: Supplementary file 2 [file pone.0355313.s002.docx]

**S2 Table. Mean and standard deviation of each acoustic parameter included in the analysis for whimpers.**

|  | **Infants** | | | | **Juveniles** | | | |
| --- | --- | --- | --- | --- | --- | --- | --- | --- |
|  | **Gombe** | | **Chimfunshi** | | **Gombe** | | **Chimfunshi** | |
|  | **M** | **SD** | **M** | **SD** | **M** | **SD** | **M** | **SD** |
| Total Elements in bout | 52.51 | 45.26 | 15.38 | 17.89 | 39.99 | 34.54 | 7.50 | 7.31 |
| Bout duration (sec) | 13.13 | 11.61 | 4.26 | 4.80 | 24.60 | 24.28 | 4.09 | 4.86 |
| Element rate | 0.28 | 0.16 | 0.27 | 0.27 | 0.55 | 0.22 | 0.43 | 0.31 |
| Delta frequency (Hz) | 3058.46 | 2428.54 | 448.08 | 534.53 | 3117.89 | 2410.86 | 1042.77 | 1013.60 |
| Q3 frequency (Hz) | 991.32 | 752.70 | 628.61 | 478.95 | 1355.00 | 791.79 | 1098.69 | 657.68 |
| Average Entropy (Bits) | 2.58 | 0.81 | 2.30 | 0.84 | 3.06 | 0.72 | 2.66 | 0.95 |
| 90% bandwidth (Hz) | 1080.44 | 1268.01 | 307.97 | 427.41 | 1351.41 | 972.37 | 768.37 | 883.91 |
| Average Power Density (db FS) | -67.65 | 8.04 | -38.67 | 6.68 | -73.27 | 8.75 | -39.51 | 6.65 |
| 50% bandwidth (Hz) | 338.80 | 569.35 | 161.64 | 285.73 | 496.13 | 522.65 | 347.74 | 486.99 |
| Frequency 5% (Hz) | 584.54 | 278.43 | 416.10 | 240.91 | 692.94 | 369.45 | 652.61 | 352.64 |
| Max Entropy (Bits) | 3.48 | 1.02 | 2.76 | 1.02 | 4.33 | 1.02 | 3.47 | 1.15 |
| Maximum frequency (Hz) | 760.89 | 508.32 | 528.35 | 395.25 | 1056.48 | 691.70 | 900.38 | 591.25 |
| Peak frequency contour maximum frequence (Hz) | 986.48 | 867.29 | 548.22 | 417.16 | 1477.27 | 1063.22 | 1098.79 | 708.24 |
| Peak frequency contour max slope (Hz/ms) | 27.30 | 60.90 | 2.00 | 7.45 | 53.40 | 66.65 | 14.06 | 24.79 |
| Peak frequency contour number of inflection points | 1.48 | 1.68 | 0.39 | 0.79 | 3.45 | 5.29 | 1.91 | 2.35 |
