# Supplementary material for: Ontogeny of dialects across chimpanzee vocalisations
Source: PLoS One. 2026 Sep 2;21(9):e0355313. doi: 10.1371/journal.pone.0355313 (PMC13537595; doi:10.1371/journal.pone.0355313)
Supplement: Supplementary file 3 [file pone.0355313.s003.docx]

**S3 Table. Mean and standard deviation of each acoustic parameter included in the analysis for grunts.**

|  | **Infants** | | | | **Juveniles** | | | |
| --- | --- | --- | --- | --- | --- | --- | --- | --- |
|  | **Gombe** | | **Chimfunshi** | | **Gombe** | | **Chimfunshi** | |
|  | **M** | **SD** | **M** | **SD** | **M** | **SD** | **M** | **SD** |
| Total Elements in bout | 16.17 | 20.30 | 1.78 | 1.26 | 33.56 | 28.62 | 2.37 | 2.87 |
| Bout duration (sec) | 10.40 | 16.19 | 0.61 | 1.37 | 16.76 | 13.97 | 0.50 | 0.94 |
| Element rate | 0.56 | 0.42 | 0.20 | 0.32 | 0.55 | 0.38 | 0.12 | 0.09 |
| Delta frequency (Hz) | 4956.20 | 4174.80 | 1048.40 | 1489.05 | 3227.22 | 2842.52 | 1180.19 | 811.45 |
| Q3 frequency (Hz) | 1017.68 | 811.36 | 1149.11 | 1467.47 | 751.37 | 649.49 | 1125.00 | 491.60 |
| Average Entropy (Bits) | 3.85 | 0.87 | 3.59 | 1.11 | 3.50 | 0.90 | 3.85 | 0.86 |
| 90% bandwidth (Hz) | 1801.77 | 1542.59 | 778.37 | 1251.06 | 1529.90 | 1370.12 | 823.66 | 634.18 |
| Average Power Density (db FS) | -71.25 | 8.44 | -40.77 | 8.09 | -67.99 | 6.95 | -36.51 | 7.50 |
| 50% bandwidth (Hz) | 568.01 | 684.49 | 433.65 | 991.24 | 482.89 | 587.75 | 347.18 | 296.73 |
| Frequency 5% (Hz) | 286.57 | 231.48 | 574.72 | 831.54 | 160.34 | 111.28 | 502.23 | 425.03 |
| Max Entropy (Bits) | 4.77 | 0.99 | 3.91 | 1.07 | 4.42 | 1.13 | 4.33 | 0.93 |
| Maximum frequency (Hz) | 579.45 | 546.70 | 872.57 | 1026.72 | 349.05 | 349.55 | 960.94 | 530.24 |
| Peak frequency contour maximum frequence (Hz) | 1083.92 | 1071.82 | 938.17 | 1112.69 | 826.27 | 886.44 | 1040.78 | 526.55 |
| Peak frequency contour max slope (Hz/ms) | 48.52 | 84.29 | 4.86 | 14.94 | 39.44 | 66.84 | 6.47 | 12.15 |
| Peak frequency contour number of inflection points | 2.15 | 2.46 | 0.49 | 1.18 | 2.49 | 2.65 | 0.48 | 0.87 |
