# Supplementary material for: Ontogeny of dialects across chimpanzee vocalisations
Source: PLoS One. 2026 Sep 2;21(9):e0355313. doi: 10.1371/journal.pone.0355313 (PMC13537595; doi:10.1371/journal.pone.0355313)
Supplement: Supplementary file 4 [file pone.0355313.s004.docx]

**S4 Table. Mean and standard deviation of each acoustic parameter included in the analysis for laughs.**

|  | **Infants** | | | | **Juveniles** | | | |
| --- | --- | --- | --- | --- | --- | --- | --- | --- |
|  | **Gombe** | | **Chimfunshi** | | **Gombe** | | **Chimfunshi** | |
|  | **M** | **SD** | **M** | **SD** | **M** | **SD** | **M** | **SD** |
| Total Elements in bout | 41.64 | 35.02 | 7.25 | 7.46 | 96.11 | 71.80 | 12.44 | 9.12 |
| Bout duration (sec) | 23.17 | 22.30 | 1.73 | 1.85 | 27.59 | 20.29 | 3.63 | 2.43 |
| Element rate | 0.53 | 0.28 | 0.21 | 0.11 | 0.29 | 0.09 | 0.29 | 0.09 |
| Delta frequency (Hz) | 4543.60 | 3772.55 | 598.28 | 722.65 | 2694.52 | 2008.63 | 1030.00 | 629.77 |
| Q3 frequency (Hz) | 1240.25 | 1117.42 | 696.53 | 772.09 | 742.83 | 618.66 | 1621.50 | 893.34 |
| Average Entropy (Bits) | 4.08 | 0.98 | 3.34 | 0.90 | 3.34 | 0.94 | 4.32 | 1.00 |
| 90% bandwidth (Hz) | 2163.81 | 1625.19 | 462.40 | 650.12 | 1344.13 | 1036.86 | 797.29 | 533.77 |
| Average Power Density (db FS) | -74.96 | 9.18 | -44.14 | 5.75 | -68.94 | 7.17 | -49.58 | 4.66 |
| 50% bandwidth (Hz) | 798.45 | 868.33 | 246.34 | 382.78 | 451.07 | 508.21 | 430.72 | 359.09 |
| Frequency 5% (Hz) | 255.97 | 353.23 | 345.46 | 601.43 | 189.82 | 187.46 | 1018.91 | 760.41 |
| Max Entropy (Bits) | 4.90 | 1.03 | 3.67 | 0.96 | 4.21 | 1.09 | 4.57 | 0.97 |
| Maximum frequency (Hz) | 588.74 | 771.66 | 564.21 | 651.65 | 382.34 | 426.99 | 1383.02 | 882.23 |
| Peak frequency contour maximum frequence (Hz) | 1356.43 | 1371.47 | 612.06 | 649.82 | 744.09 | 767.62 | 1519.12 | 905.38 |
| Peak frequency contour max slope (Hz/ms) | 77.63 | 107.45 | 3.99 | 6.90 | 37.18 | 57.59 | 13.06 | 16.39 |
| Peak frequency contour number of inflection points | 3.85 | 3.38 | 0.69 | 1.33 | 3.72 | 3.67 | 1.04 | 1.52 |
