# Supplementary material for: Ontogeny of dialects across chimpanzee vocalisations
Source: PLoS One. 2026 Sep 2;21(9):e0355313. doi: 10.1371/journal.pone.0355313 (PMC13537595; doi:10.1371/journal.pone.0355313)
Supplement: Supplementary file 5 [file pone.0355313.s005.docx]

S5 ‍Table. Factor loading of infant grunt PDFA model. Positive values = Gombe. Negative values = Chimfunshi.

| **Variable** | **Mean loading** | **Standard deviation loading** | **Median loading** | **Lower confidence interval** | **Upper confidence interval** | **Proportion of the same sign** |
| --- | --- | --- | --- | --- | --- | --- |
| Average Entropy (Bits) | -8.28 | 10.21 | -5.16 | -35.24 | 2.11 | 0.89 |
| Maximum Entropy (Bits) | 6.52 | 9.32 | 3.73 | -4.94 | 27.69 | 0.76 |
| Element rate (s-1) | 0.66 | 12.89 | 0.26 | -21.97 | 29.59 | 0.52 |
| Bout duration (seconds) | -0.41 | 3.40 | -0.27 | -7.09 | 4.5 | 0.61 |
| Total elements in bout | 0.32 | 1.49 | 0.19 | -2.32 | 3.54 | 0.66 |
| Average Power Density (db FS) | -0.07 | 0.43 | -0.16 | -0.73 | 0.94 | 0.66 |
| Peak frequency contour max slope (Hz/ms) | 0.01 | 0.21 | 0.02 | -0.37 | 0.41 | 0.59 |
| Frequency 5% (Hz) | 0.00 | 0.03 | 0.00 | -0.06 | 0.07 | 0.53 |
| 50% bandwidth (Hz) | 0.00 | 0.04 | 0.00 | -0.07 | 0.07 | 0.53 |
| Q3 frequency (Hz) | 0.00 | 0.04 | 0.00 | -0.08 | 0.08 | 0.5 |
| 90% bandwidth (Hz) | 0.00 | 0.01 | 0.00 | -0.03 | 0.04 | 0.53 |
| Delta frequency (Hz) | 0.00 | 0.01 | 0.00 | -0.01 | 0.00 | 0.6 |
| Peak frequency contour maximum frequency (Hz) | 0 | 0.02 | 0 | -0.05 | 0.04 | 0.47 |
| Maximum frequency (Hz) | 0 | 0.02 | 0 | -0.03 | 0.04 | 0.46 |
