# Supplementary material for: Ontogeny of dialects across chimpanzee vocalisations
Source: PLoS One. 2026 Sep 2;21(9):e0355313. doi: 10.1371/journal.pone.0355313 (PMC13537595; doi:10.1371/journal.pone.0355313)
Supplement: Supplementary file 6 [file pone.0355313.s006.docx]

S6‍ Table. Factor loadings of infant whimper PDA model. Positive values = Gombe. Negative values = Chimfunshi.

| **Variable** | **Mean loading** | **Standard deviation loading** | **Median loading** | **Lower confidence interval** | **Upper confidence interval** | **Proportion of the same sign** | |
| --- | --- | --- | --- | --- | --- | --- | --- |
| Average Entropy (Bits) | -7.41 | 7.54 | -4.75 | -23.57 | 0.16 | 0.96 |  |
| Maximum Entropy (Bits) | 4.60 | 7.57 | 3.34 | -3.56 | 24.26 | 0.86 |  |
| Element rate (s-1) | -0.95 | 21.04 | -0.65 | -26.51 | 31.27 | 0.53 |  |
| Bout duration (seconds) | -0.28 | 3.09 | -0.34 | -5.06 | 4.44 | 0.58 |  |
| Total elements in bout | 0.10 | 0.75 | 0.08 | -1.04 | 1.60 | 0.59 |  |
| Average Power Density (db FS) | -0.05 | 0.55 | -0.19 | -0.93 | 1.26 | 0.64 |  |
| Frequency 5% (Hz) | -0.03 | 0.18 | -0.01 | -0.31 | 0.17 | 0.59 |  |
| Peak frequency contour max slope (Hz/ms) | 0.02 | 0.72 | -0.03 | -1.18 | 2.00 | 0.43 |  |
| Q3 frequency (Hz) | 0.02 | 0.19 | 0.00 | -0.23 | 0.31 | 0.48 |  |
| Maximum frequency (Hz) | 0.01 | 0.09 | 0.00 | -0.19 | 0.25 | 0.60 |  |
| 50% bandwidth (Hz) | -0.01 | 0.17 | 0.00 | -0.28 | 0.21 | 0.40 |  |
| Peak frequency contour maximum frequency (Hz) | -0.01 | 0.07 | 0.00 | -0.22 | 0.11 | 0.46 |  |
| 90% bandwidth (Hz) | 0.00 | 0.01 | 0.00 | -0.02 | 0.03 | 0.63 |  |
| Delta frequency (Hz) | 0.00 | 0.01 | 0.00 | -0.01 | 0.01 | 0.59 |  |
