# Supplementary material for: Ontogeny of dialects across chimpanzee vocalisations
Source: PLoS One. 2026 Sep 2;21(9):e0355313. doi: 10.1371/journal.pone.0355313 (PMC13537595; doi:10.1371/journal.pone.0355313)
Supplement: Supplementary file 7 [file pone.0355313.s007.docx]

‍S7 Table. Factor loading of infant laugh PDFA model. Positive values = Gombe. Negative values = Chimfunshi.

| **Variable** | **Mean loading** | **Standard deviation loading** | **Median loading** | **Lower confidence interval** | **Upper confidence interval** | **Proportion of the same sign** | |
| --- | --- | --- | --- | --- | --- | --- | --- |
| Element rate (s-1) | 1.91 | 1.31 | 1.81 | -0.26 | 4.49 | 0.93 |  |
| Average Entropy (Bits) | 0.37 | 1.44 | 0.55 | -2.33 | 2.88 | 0.64 |  |
| Maximum Entropy (Bits) | -0.11 | 1.05 | -0.17 | -2.03 | 2.13 | 0.57 |  |
| Bout duration (seconds) | -0.08 | 0.27 | -0.04 | -0.64 | 0.24 | 0.74 |  |
| Total elements in bout | 0.03 | 0.08 | 0.02 | -0.15 | 0.17 | 0.67 |  |
| Average Power Density (db FS) | 0.02 | 0.24 | 0.18 | -0.30 | 0.32 | 0.54 |  |
| Peak frequency contour max slope (Hz/ms) | 0.00 | 0.02 | 0.00 | -0.03 | 0.04 | 0.56 |  |
| Frequency 5% (Hz) | 0.00 | 0.01 | 0.00 | -0.01 | 0.01 | 0.56 |  |
| Q3 frequency (Hz) | 0.00 | 0.01 | 0.00 | -0.01 | 0.01 | 0.52 |  |
| Maximum frequency (Hz) | 0.00 | 0.00 | 0.00 | 0.00 | 0.00 | 0.51 |  |
| 90% bandwidth (Hz) | 0.00 | 0.00 | 0.00 | 0.00 | 0.00 | 0.54 |  |
| 50% bandwidth (Hz) | 0.00 | 0.01 | 0.00 | -0.01 | 0.01 | 0.51 |  |
| Delta frequency (Hz) | 4.24E-05 | 0 | 1.45E-05 | 0 | 0 | 0.53 |  |
| Peak frequency contour maximum frequency (Hz) | 1.67E-05 | 0 | -8.18E-05 | 0 | 0 | 0.48 |  |
