# Supplementary material for: Ontogeny of dialects across chimpanzee vocalisations
Source: PLoS One. 2026 Sep 2;21(9):e0355313. doi: 10.1371/journal.pone.0355313 (PMC13537595; doi:10.1371/journal.pone.0355313)
Supplement: Supplementary file 8 [file pone.0355313.s008.docx]

S8‍ Table. Factor loadings of juvenile grunt PDFA model. Positive values = Gombe. Negative values = Chimfunshi.

| **Variable** | **Mean loading** | **Standard deviation loading** | **Median loading** | **Lower confidence interval** | **Upper confidence interval** | **Proportion of the same sign** | |
| --- | --- | --- | --- | --- | --- | --- | --- |
| Element rate (s-1) | 3.76 | 4.03 | 2.22 | -0.28 | 13.41 | 0.94 |  |
| Average Entropy (Bits) | -0.22 | 0.73 | -0.17 | -1.55 | 1.23 | 0.65 |  |
| Maximum Entropy (Bits) | -0.13 | 0.61 | -0.08 | -1.29 | 0.85 | 0.56 |  |
| Average Power Density (db FS) | -0.06 | 0.16 | -0.08 | -0.35 | 0.27 | 0.77 |  |
| Bout duration (seconds) | 0.03 | 0.07 | 0.02 | -0.12 | 0.24 | 0.67 |  |
| Peak frequency contour max slope (Hz/ms) | 0.01 | 0.04 | 0.00 | -0.05 | 0.10 | 0.61 |  |
| Total elements in bout | 0.00 | 0.06 | 0.00 | -0.15 | 0.11 | 0.47 |  |
| Frequency 5% (Hz) | 0.00 | 0.01 | 0.00 | -0.02 | 0.01 | 0.48 |  |
| Maximum frequency (Hz) | 0.00 | 0.00 | 0.00 | 0.00 | 0.00 | 0.64 |  |
| 50% bandwidth (Hz) | 0.00 | 0.00 | 0.00 | 0.00 | 0.00 | 0.55 |  |
| Delta frequency (Hz) | 1.10E-04 | 0 | 5.75E-05 | 0 | 0 | 0.61 |  |
| Peak frequency contour maximum frequency (Hz) | 6.75E-05 | 0 | 9.85E-05 | 0 | 0 | 0.6 |  |
| 90% bandwidth (Hz) | 5.02E-05 | 0 | -6.15E-06 | 0 | 0 | 0.5 |  |
| Q3 frequency (Hz) | 1.19E-05 | 0 | 7.18E-05 | 0 | 0 | 0.55 |  |
