# Supplementary material for: Ontogeny of dialects across chimpanzee vocalisations
Source: PLoS One. 2026 Sep 2;21(9):e0355313. doi: 10.1371/journal.pone.0355313 (PMC13537595; doi:10.1371/journal.pone.0355313)
Supplement: Supplementary file 9 [file pone.0355313.s009.docx]

‍S9 Table. Factor loadings for juveniles whimper PDFA model. Positive values = Gombe. Negative values = Chimfunshi.

| **Variable** | **Mean loading** | **Standard deviation loading** | **Median loading** | **Lower confidence interval** | **Upper confidence interval** | **Proportion of the same sign** | |
| --- | --- | --- | --- | --- | --- | --- | --- |
| Element rate (s-1) | 1.80 | 11.16 | 0.69 | -12.02 | 20.18 | 0.58 |  |
| Average Entropy (Bits) | -1.77 | 3.18 | -1.67 | -9.10 | 2.21 | 0.78 |  |
| Maximum Entropy (Bits) | 0.98 | 2.82 | 0.75 | -4.38 | 6.79 | 0.71 |  |
| Total elements in bout | -0.04 | 0.49 | -0.02 | -0.69 | 0.54 | 0.55 |  |
| Average Power Density (db FS) | 0.01 | 0.32 | 0.15 | -0.58 | 0.47 | 0.54 |  |
| Bout duration (seconds) | -0.01 | 0.72 | 0.02 | -1.04 | 1.05 | 0.47 |  |
| Peak frequency contour max slope (Hz/ms) | 0.00 | 0.13 | -0.01 | -0.21 | 0.34 | 0.47 |  |
| Q3 frequency (Hz) | 0.00 | 0.02 | 0.00 | -0.03 | 0.04 | 0.55 |  |
| Frequency 5% (Hz) | 0.00 | 0.01 | 0.00 | -0.03 | 0.02 | 0.5 |  |
| Peak frequency contour maximum frequency (Hz) | 0.00 | 0.02 | 0.00 | -0.03 | 0.03 | 0.54 |  |
| 50% bandwidth (Hz) | 0.00 | 0.02 | 0.00 | -0.03 | 0.04 | 0.53 |  |
| Maximum frequency (Hz) | 0.00 | 0.01 | 0.00 | -0.02 | 0.02 | 0.56 |  |
| 90% bandwidth (Hz) | 1.49E-04 | 0 | 2.38E-04 | -0.01 | 0.01 | 0.56 |  |
| Delta frequency (Hz) | 3.74E-05 | 0 | -7.19E-05 | 0 | 0 | 0.48 |  |
