# Supplementary material for: Ontogeny of dialects across chimpanzee vocalisations
Source: PLoS One. 2026 Sep 2;21(9):e0355313. doi: 10.1371/journal.pone.0355313 (PMC13537595; doi:10.1371/journal.pone.0355313)
Supplement: Supplementary file 10 [file pone.0355313.s010.docx]

**S10 Table. Factor loadings for juvenile laugh PDFA model. Positive values = Gombe. Negative values = Chimfunshi.**

| **Variable** | **Mean loading** | **Standard deviation loading** | **Median loading** | **Lower confidence interval** | **Upper confidence interval** | **Proportion of the same sign** | |
| --- | --- | --- | --- | --- | --- | --- | --- |
| Element rate (s-1) | -3.22 | 2.84 | -3.40 | -7.78 | 2.18 | 0.81 |  |
| Maximum Entropy (Bits) | 0.29 | 0.65 | 0.28 | -0.90 | 1.52 | 0.67 |  |
| Average Entropy (Bits) | 0.19 | 1.14 | 0.57 | -2.11 | 1.63 | 0.67 |  |
| Average Power Density (db FS) | 0.08 | 0.15 | 0.16 | -0.20 | 0.21 | 0.75 |  |
| Bout duration (seconds) | 0.02 | 0.08 | 0.01 | -0.11 | 0.19 | 0.55 |  |
| Total elements in bout | -0.01 | 0.03 | -0.01 | -0.07 | 0.04 | 0.69 |  |
| Peak frequency contour max slope (Hz/ms) | 0.00 | 0.01 | 0.00 | -0.03 | 0.02 | 0.53 |  |
| Frequency 5% (Hz) | 0.00 | 0.00 | 0.00 | 0.00 | 0.01 | 0.7 |  |
| Q3 frequency (Hz) | 0.00 | 0.00 | 0.00 | -0.01 | 0.00 | 0.65 |  |
| 50% bandwidth (Hz) | 0.00 | 0.00 | 0.00 | 0.00 | 0.00 | 0.64 |  |
| Delta frequency (Hz) | 0.00 | 0.00 | 0.00 | 0.00 | 0.00 | 0.76 |  |
| Maximum frequency (Hz) | 0.00 | 0.00 | 0.00 | 0.00 | 0.00 | 0.53 |  |
| Peak frequency contour maximum frequency (Hz) | 0.00 | 0.00 | 0.00 | 0.00 | 0.00 | 0.54 |  |
| 90% bandwidth (Hz) | 5.47E-05 | 0 | -7.93E-05 | 0 | 0 | 0.44 |  |
