# Supplementary material for: Ontogeny of dialects across chimpanzee vocalisations
Source: PLoS One. 2026 Sep 2;21(9):e0355313. doi: 10.1371/journal.pone.0355313 (PMC13537595; doi:10.1371/journal.pone.0355313)
Supplement: Supplementary file 12 [file pone.0355313.s013.docx]

\# 1 Set up & Prepare data ####

library(readxl)

library(nnet)

library(MASS)

library(caret)

library(e1071)

library(dplyr) # for piping

library(tidyr) # for neat code

library(stringr) # for working with character strings

library(forcats) #for categorical data

library(ggplot2)

\# Load in excel dataset

Acoustic_Data <- read_excel("2026 Acoustic Dataset.xlsx",

sheet = 1)

Acoustic_Data$ID_Individual <- as.factor(Acoustic_Data$ID_Individual)

\# 2. pDFA 1 ----

\# load package MASS

library("MASS")

pDFA.nested<-function(test.fac, contr.fac=NULL, variables, restrict.by=NULL,

n.contr.fac.levels.to.sel=NULL, n.to.sel.per.contr.fac.level=NULL,

n.sel=100, n.perm=1000, pdfa.data){

#version from Feb 04 2014

if(length(contr.fac)==0){

contr.fac="contr.fac"

pdfa.data$contr.fac=as.factor(1:nrow(pdfa.data))

}

if(sum(c(test.fac, contr.fac, variables)%in%names(pdfa.data))<(length(variables)+1)){

stop("not all variables are in pdfa.data")

}

if(max(apply(X=table(pdfa.data[, contr.fac], pdfa.data[, test.fac])>0, MARGIN=1, FUN=sum))>1){

stop("Error: at least one level of contr.fac occurs in more than one level of test.fac")

}

if(length(restrict.by)==0){

restrict.by="restrict.by"

pdfa.data$restrict.by=as.factor(rep(1, nrow(pdfa.data)))

}

pdfa.data=data.frame(droplevels(na.omit(pdfa.data[, c(variables, test.fac, contr.fac, restrict.by)])))

pdfa.data[, test.fac]=as.factor(as.character(pdfa.data[, test.fac]))

pdfa.data[, contr.fac]=as.factor(as.character(pdfa.data[, contr.fac]))

pdfa.data[, restrict.by]=as.factor(as.character(pdfa.data[, restrict.by]))

model=as.formula(paste(c(test.fac, paste(variables, collapse="+")), collapse="~"))

ncf.levels=length(levels(pdfa.data[,contr.fac]))

ntf.levels=length(levels(pdfa.data[,test.fac]))

pr.prob=rep(1/ntf.levels, ntf.levels)#define prior probabilities to be equal for either of two groups

#get minimum number of perm.fac levels per level of test.fac:

if(length(n.contr.fac.levels.to.sel)==0){

n.contr.fac.levels.to.sel=min(apply(table(pdfa.data[,test.fac], pdfa.data[,contr.fac])>0, 1, sum))

}else if(n.contr.fac.levels.to.sel>min(apply(table(pdfa.data[,test.fac], pdfa.data[,contr.fac])>0, 1, sum))){

stop("Error: n.contr.fac.levels.to.sel is larger than minimum number of levels of contr.fac per level of test.fac")

}

if(length(n.to.sel.per.contr.fac.level)==0){

n.to.sel.per.contr.fac.level=min(table(pdfa.data[, contr.fac]))

}else if(n.to.sel.per.contr.fac.level>min(table(pdfa.data[, contr.fac]))){

stop("Error: n.to.sel.per.contr.fac.level is larger than minimum per level of contr.fac")

}

#sort data set to make it easier to permute:

pdfa.data=pdfa.data[order(pdfa.data[, restrict.by], pdfa.data[, test.fac], pdfa.data[, contr.fac]), ]

#create list with 1s and 0s to generate random selections within levels of contr.fac:

to.sel.from=lapply(as.vector(unique(pdfa.data[, contr.fac])), function(xcf){

c(rep(1, n.to.sel.per.contr.fac.level), rep(0, sum(pdfa.data[, contr.fac]==xcf)-n.to.sel.per.contr.fac.level))

})

#create list with levels of contr.fac per level of test.fac:

control.fac.levels.to.sel.from=lapply(as.vector(unique(pdfa.data[, test.fac])), function(xtf){

as.vector(unique(pdfa.data[pdfa.data[, test.fac]==xtf, contr.fac]))

})

#initialise test statistics:

conf.mat.sel=matrix(0, nrow=ntf.levels, ncol=ntf.levels)

colnames(conf.mat.sel)=unique(as.vector(pdfa.data[, test.fac]))

rownames(conf.mat.sel)=unique(as.vector(pdfa.data[, test.fac]))

conf.mat.cval=conf.mat.sel

conf.mat.sel.exp=conf.mat.sel

conf.mat.cval.exp=conf.mat.sel

conf.mat.cval.p=conf.mat.cval

conf.mat.sel.p=conf.mat.cval

conf.mat.obs=matrix(0, nrow=length(levels(pdfa.data[, test.fac])), ncol=length(levels(pdfa.data[, test.fac])))

conf.mat.exp=conf.mat.obs

ur.c.val=0

ur.sel=0

number=(1:nrow(pdfa.data))

correct.mat = matrix(0, nrow = nrow(pdfa.data), ncol = n.sel)

\# store discriminant coefficients from each DFA

scaling_list <- vector("list", n.sel)

for (k in 1:n.sel){

#make random selection of same number of cases per subject

sel=unlist(lapply(to.sel.from, function(x){

sample(x, length(x), replace=F)

}))

sel.perm.fac.levs=unlist(lapply(control.fac.levels.to.sel.from, function(xpf){

sample(xpf, n.contr.fac.levels.to.sel, replace=F)

}))

sel.index=number[sel==1 & pdfa.data[, contr.fac]%in%sel.perm.fac.levs]

#do a DFA and store results in 'res':

res <- lda(model, prior = pr.prob, data = pdfa.data[sel.index, ])

\# store discriminant coefficients

scaling_list[[k]] <- res$scaling

#get predictions and store them in 'pred':

pred=predict(res, pdfa.data, prior=pr.prob)$class

correct.mat[, k] = as.integer(pdfa.data[, test.fac] == pred)

ur.sel=ur.sel+sum((pdfa.data[, test.fac]==pred)[sel.index])

ur.c.val= ur.c.val+ sum((pdfa.data[, test.fac]==pred)[-sel.index])

conf.mat.obs=conf.mat.obs+table(pdfa.data[-sel.index, test.fac], pred[-sel.index])

conf.mat.sel=conf.mat.sel+table(pdfa.data[sel.index, test.fac], pred[sel.index])

conf.mat.cval=conf.mat.cval+table(pdfa.data[-sel.index, test.fac], pred[-sel.index])

}

conf.mat.obs=conf.mat.obs/n.sel

conf.mat.sel=conf.mat.sel/n.sel

conf.mat.cval=conf.mat.cval/n.sel

#save number of correctly classified calls in variable 'orig.res':

ur.sel= ur.sel/n.sel

ur.c.val= ur.c.val/n.sel

#set P-value to 1 (since original data should be treated as 1 permutation):

p.sel=1

p.c.val=1

all.corr=matrix(NA, nrow=n.perm, ncol=2)

all.corr[1,1]=ur.sel

all.corr[1,2]=ur.c.val

#reformulate model for randomized data:

model=as.formula(paste(c("r.tf", paste(variables, collapse="+")), collapse="~"))

#determine levels of test.fac:

tf.lev=levels(pdfa.data[, test.fac])

#determine levels of test.fac per level of contr.fac, separately per level of restict.by:

test.fac.per.lev.of.control.fac=lapply(as.vector(unique(pdfa.data[, restrict.by])), function(xrf){

unlist(lapply(unique(as.character(pdfa.data[pdfa.data[, restrict.by]==xrf, contr.fac])), function(xcf){

unique(as.character(pdfa.data[pdfa.data[, contr.fac]==xcf, test.fac]))

}))

})

#now the permutation:

for (k in 1:(n.perm-1)){

#randomize contr.facs assignments to test.fac:

r.tf=unlist(lapply(test.fac.per.lev.of.control.fac, function(x){

sample(x, length(x), replace=F)

}))

#make random selection of same number of cases per subject

sel=unlist(lapply(to.sel.from, function(x){

sample(x, length(x), replace=F)

}))

r.tf=as.factor(r.tf[match(pdfa.data[, contr.fac], as.vector(unique(pdfa.data[, contr.fac])))])

rpdfa.data=data.frame(pdfa.data, r.tf, sel)

rm(r.tf, sel)

rpdfa.data=rpdfa.data[order(rpdfa.data[, "r.tf"], rpdfa.data[, test.fac], rpdfa.data[, contr.fac]), ]

#create list with levels of perm.fac per level of test.fac:

control.fac.levels.to.sel.from=lapply(as.vector(unique(rpdfa.data[, "r.tf"])), function(xtf){

as.vector(unique(rpdfa.data[rpdfa.data[, "r.tf"]==xtf, contr.fac]))

})

r.cf=unlist(lapply(control.fac.levels.to.sel.from, function(x){

sample(x, n.contr.fac.levels.to.sel, replace=F)

}))

sel.index=number[rpdfa.data$sel==1 & rpdfa.data[, contr.fac]%in%r.cf]

#do a DFA and store results in 'res':

res=lda(model, prior=pr.prob, data=rpdfa.data[sel.index, ])

#get predictions and store them in 'pred':

pred=predict(res, rpdfa.data, prior=pr.prob)$class

ran.sel=sum((rpdfa.data[, "r.tf"]==pred)[sel.index])

ran.c.val=sum((rpdfa.data[, "r.tf"]==pred)[-sel.index])

conf.mat.exp=conf.mat.exp+table(rpdfa.data[-sel.index, "r.tf"], pred[-sel.index])

if (ran.sel>=ur.sel){p.sel = p.sel + 1}

if (ran.c.val>= ur.c.val){p.c.val= p.c.val + 1}

all.corr[k+1,1]=ran.sel

all.corr[k+1,2]=ran.c.val

conf.mat.sel.exp=conf.mat.sel.exp+table(pdfa.data[sel.index, test.fac], pred[sel.index])

conf.mat.cval.exp=conf.mat.cval.exp+table(pdfa.data[-sel.index, test.fac], pred[-sel.index])

conf.mat.sel.p=conf.mat.sel.p+as.numeric(table(pdfa.data[sel.index, test.fac], pred[sel.index])>=conf.mat.cval)

conf.mat.cval.p=conf.mat.cval.p+as.numeric(table(pdfa.data[-sel.index, test.fac], pred[-sel.index])>=conf.mat.cval)

}

conf.mat.exp=conf.mat.exp/(n.perm-1)

conf.mat.sel.exp=conf.mat.sel.exp/(n.perm-1)

conf.mat.cval.exp=conf.mat.cval.exp/(n.perm-1)

conf.mat.sel.p=(conf.mat.sel.p+1)/n.perm

conf.mat.cval.p=(conf.mat.cval.p+1)/n.perm

correct.rate <- rowMeans(correct.mat)

what=c("no.corr.classified.selected", "expected.no.corr.classified.selected",

"percent.correctly.classified.selected",

"expected.percent.correctly.classified.selected",

"P.for.selected",

"no.corr.cross.classified", "expected.no.corr.cross.classified",

"percent.corr.cross.classified", "expected.percent.corr.cross.classified", "P.for.cross.classified",

"no.levels.contr.factor", "no.levels.contr.factor.selected", "no.levels.test.factor", "no.cases",

"no.cases.selected", "no.cases.selected.per.level.of.control.fac", "no.permutations", "no.random.selections")

#... glue all the results together

result=c(ur.sel, mean(all.corr[,1]),

100*ur.sel/(ntf.levels*n.contr.fac.levels.to.sel*n.to.sel.per.contr.fac.level),

100*mean(mean(all.corr[,1]))/(ntf.levels*n.contr.fac.levels.to.sel*n.to.sel.per.contr.fac.level),

p.sel/n.perm,

ur.c.val, mean(all.corr[,2]), 100*ur.c.val/(nrow(pdfa.data)-ntf.levels*n.contr.fac.levels.to.sel*n.to.sel.per.contr.fac.level),

100*mean(all.corr[,2])/(nrow(pdfa.data)-ntf.levels*n.contr.fac.levels.to.sel*n.to.sel.per.contr.fac.level), p.c.val/n.perm,

ncf.levels, ntf.levels*n.contr.fac.levels.to.sel,

ntf.levels, nrow(pdfa.data), ntf.levels*n.contr.fac.levels.to.sel*n.to.sel.per.contr.fac.level, n.to.sel.per.contr.fac.level,

n.perm, n.sel)

names(result)=what

return(list(

result = result,

scaling = scaling_list,

conf.mat.sel = conf.mat.sel,

exp.conf.mat.sel = conf.mat.sel.exp,

p.conf.mat.sel = conf.mat.sel.p,

conf.mat.cval = conf.mat.cval,

exp.conf.mat.cval = conf.mat.cval.exp,

p.conf.mat.cval = conf.mat.cval.p,

correct.mat = correct.mat,

correct.rate = correct.rate

))

}

\# Sites - Can we discriminate between sites?

\# Is the discrimination of individuals (control) within different age groups (restriction)

\# more accurate for one site than the other (test)

\# For this analysis, we are testing Whimpers, Laughs and Whimpers in separate models.

\# This is due to the low numbers of observations of other vocalisations.

\# For this analysis, we are testing Infants and Juveniles only. This is due to

\# low numbers of observations from other age groups and being uneven between sites.

\# test.fac, character = factor tested for discriminality <- "Site"

\# contr.fac, character = factor to be controlled for <- "ID_Individual"

\# variables, character = variables to be inluded into the DFA <-

\# "Total.Elements.in.bout" "Bout.duration..sec."	"Element.rate"

\# "Delta_Freq"	"q3_freq"

\# "Avg_Entropy"	"BW_90%"

\# "Avg_Power_Density"	"BW_50%"

\# "Freq_5%"	"Max_Entropy"

\# "Max_Freq"	"PFC_Max_Freq"

\# "PFC_Max_Slope"	"PFC_Num_Inf_Pts"

\# restrict.by, character = factor by which the permutation should be restricted <- "Age group"

\## Model 1: Grunts ----

#Filter for Grunts, exclude

Grunts <- Acoustic_Data %>%

filter(Vocalisation == "Grunt") %>%

filter(`Age group` == "Infant" | `Age group` == "Juvenile")

write.table(Grunts, file = "pdfa.grunt.data.txt", sep = "\t", row.names = FALSE)

pdfa.grunt.data=read.table(file="pdfa.grunt.data.txt",

header=T, sep="\t", fill=T, dec=".")

summary(pdfa.grunt.data)

names(pdfa.grunt.data) # for correct variable naming

\# # > names(pdfa.grunt.data)

\# [1] "row_id"	"ID_Bout"	"ID_Individual"	"Site"

\# [5] "Age.group"	"Vocalisation"	"Total.Elements.in.bout" "Bout.duration..sec."

\# [9] "Element.rate"	"Delta_Freq"	"q3_freq"	"Avg_Entropy"

\# [13] "BW_90."	"Avg_Power_Density"	"BW_50."	"Freq_5."

\# [17] "Max_Entropy"	"Max_Freq"	"PFC_Max_Freq"	"PFC_Max_Slope"

\# [21] "PFC_Num_Inf_Pts"

pdfa.Grunts=pDFA.nested(test.fac="Site",

contr.fac="ID_Individual",

variables=c("Total.Elements.in.bout", "Bout.duration..sec.",

"Element.rate", "Delta_Freq",

"q3_freq", "Avg_Entropy", "BW_90.", "Avg_Power_Density",

"BW_50.", "Freq_5.", "Max_Entropy", "Max_Freq",

"PFC_Max_Freq", "PFC_Max_Slope", "PFC_Num_Inf_Pts"),

restrict.by = "Age.group",

n.contr.fac.levels.to.sel= NULL,

n.to.sel.per.contr.fac.level=NULL, n.sel=100, n.perm=1000,

pdfa.data=pdfa.grunt.data)

Grunts_Result <- as.data.frame(pdfa.Grunts$result)

Grunts_Result

\## Model 2: Whimpers ----

#Filter for Whimpers, exclude

Whimpers <- Acoustic_Data %>%

filter(Vocalisation == "Whimper") %>%

filter(`Age group` == "Infant" | `Age group` == "Juvenile")

write.table(Whimpers, file = "pdfa.whimper.data.txt", sep = "\t", row.names = FALSE)

pdfa.whimper.data=read.table(file="pdfa.whimper.data.txt",

header=T, sep="\t", fill=T, dec=".")

summary(pdfa.whimper.data)

names(pdfa.whimper.data) # for correct variable naming in the model

\# > names(pdfa.whimper.data)

\# [1] "row_id"	"ID_Bout"	"ID_Individual"	"Site"

\# [5] "Age.group"	"Vocalisation"	"Total.Elements.in.bout" "Bout.duration..sec."

\# [9] "Element.rate"	"Delta_Freq"	"q3_freq"	"Avg_Entropy"

\# [13] "BW_90."	"Avg_Power_Density"	"BW_50."	"Freq_5."

\# [17] "Max_Entropy"	"Max_Freq"	"PFC_Max_Freq"	"PFC_Max_Slope"

\# [21] "PFC_Num_Inf_Pts"

pdfa.Whimpers=pDFA.nested(test.fac="Site",

contr.fac="ID_Individual",

variables=c("Total.Elements.in.bout", "Bout.duration..sec.",

"Element.rate", "Delta_Freq",

"q3_freq", "Avg_Entropy", "BW_90.", "Avg_Power_Density",

"BW_50.", "Freq_5.", "Max_Entropy", "Max_Freq",

"PFC_Max_Freq", "PFC_Max_Slope", "PFC_Num_Inf_Pts"),

restrict.by = "Age.group",

n.contr.fac.levels.to.sel= NULL,

n.to.sel.per.contr.fac.level=NULL, n.sel=100, n.perm=1000,

pdfa.data=pdfa.whimper.data)

Whimpers_Result <- as.data.frame(pdfa.Whimpers$result)

Whimpers_Result

\## Model 3: Laughs ----

#Filter for Laughs, exclude

Laughs <- Acoustic_Data %>%

filter(Vocalisation == "Laugh") %>%

filter(`Age group` == "Infant" | `Age group` == "Juvenile")

write.table(Laughs, file = "pdfa.laugh.data.txt", sep = "\t", row.names = FALSE)

pdfa.laugh.data=read.table(file="pdfa.laugh.data.txt",

header=T, sep="\t", fill=T, dec=".")

summary(pdfa.laugh.data)

names(pdfa.laugh.data) # for correct variable naming in the model

\# > names(pdfa.laugh.data) # for correct variable naming in the model

\# [1] "row_id"	"ID_Bout"	"ID_Individual"	"Site"

\# [5] "Age.group"	"Vocalisation"	"Total.Elements.in.bout" "Bout.duration..sec."

\# [9] "Element.rate"	"Delta_Freq"	"q3_freq"	"Avg_Entropy"

\# [13] "BW_90."	"Avg_Power_Density"	"BW_50."	"Freq_5."

\# [17] "Max_Entropy"	"Max_Freq"	"PFC_Max_Freq"	"PFC_Max_Slope"

\# [21] "PFC_Num_Inf_Pts"

pdfa.Laughs=pDFA.nested(test.fac="Site",

contr.fac="ID_Individual",

variables=c("Total.Elements.in.bout", "Bout.duration..sec.",

"Element.rate", "Delta_Freq",

"q3_freq", "Avg_Entropy", "BW_90.", "Avg_Power_Density",

"BW_50.", "Freq_5.", "Max_Entropy", "Max_Freq",

"PFC_Max_Freq", "PFC_Max_Slope", "PFC_Num_Inf_Pts"),

restrict.by = "Age.group",

n.contr.fac.levels.to.sel= NULL,

n.to.sel.per.contr.fac.level=NULL, n.sel=100, n.perm=1000,

pdfa.data=pdfa.laugh.data)

Laughs_Result <- as.data.frame(pdfa.Laughs$result)

Laughs_Result

\# 3. pDFA 2 ----

\# Are the differences in the disciminality of juveniles and infants between each site?

\# Is the discrimination of infants and juveniles (control) more accurate for one site than the other (test)

\# How diff the percentages of correct class are between the two models of infants vs juveniles fr each call type

\# For this analysis, we are testing Whimpers, Laughs and Whimpers of Juveniles and Infants in separate models.

\## Model 1a: Grunts - Infants ----

#Filter for Grunts in infants

Grunts_Infants <- Acoustic_Data %>%

filter(Vocalisation == "Grunt") %>%

filter(`Age group` == "Infant")

Grunts_Infants_Age <- Grunts_Infants %>%

group_by(Site, ID_Individual, `Age group`) %>%

summarise(n=n())

n_distinct(Grunts_Infants$ID_Individual[Grunts_Infants$Site == "Chimfunshi"])#9

n_distinct(Grunts_Infants$ID_Individual[Grunts_Infants$Site == "Gombe"])#9

write.table(Grunts_Infants, file = "pdfa.grunt.infant.data.txt", sep = "\t", row.names = FALSE)

pdfa.grunt.infant.data=read.table(file="pdfa.grunt.infant.data.txt",

header=T, sep="\t", fill=T, dec=".")

summary(pdfa.grunt.infant.data)

names(pdfa.grunt.infant.data) # for correct variable naming

\# > names(pdfa.grunt.infant.data) # for correct variable naming

\# [1] "row_id"	"ID_Bout"	"ID_Individual"	"Site"

\# [5] "Age.group"	"Vocalisation"	"Total.Elements.in.bout" "Bout.duration..sec."

\# [9] "Element.rate"	"Delta_Freq"	"q3_freq"	"Avg_Entropy"

\# [13] "BW_90."	"Avg_Power_Density"	"BW_50."	"Freq_5."

\# [17] "Max_Entropy"	"Max_Freq"	"PFC_Max_Freq"	"PFC_Max_Slope"

\# [21] "PFC_Num_Inf_Pts"

pdfa.Grunts.Infants=pDFA.nested(test.fac="Site",

contr.fac="ID_Individual",

variables=c("Total.Elements.in.bout", "Bout.duration..sec.",

"Element.rate", "Delta_Freq",

"q3_freq", "Avg_Entropy", "BW_90.", "Avg_Power_Density",

"BW_50.", "Freq_5.", "Max_Entropy", "Max_Freq",

"PFC_Max_Freq", "PFC_Max_Slope"),

restrict.by = NULL,

n.contr.fac.levels.to.sel= NULL,

n.to.sel.per.contr.fac.level=NULL, n.sel=100, n.perm=1000,

pdfa.data=pdfa.grunt.infant.data)

Grunts.Infants_Result <- as.data.frame(pdfa.Grunts.Infants$result)

Grunts.Infants_Result

\# recalculates the data point after dropping it - percentage of how often that data point is classified correctly

pdfa.grunt.infant.data$class_rate <- pdfa.Grunts.Infants$correct.rate

\# use this variable for model - non-binary model - use a beta/gamma distribution (beta probably)

\# start with mixed model - add random effect for individual

\# predict class.rate according to age group (juvenile vs infant)

\# - if it turns out tricky, do a simple model (linear model) procedures account for non-independence in pdfa

mean(pdfa.grunt.infant.data$class_rate)

pdfa.Grunts.Infants$result["percent.corr.cross.classified"] / 100

hist(pdfa.grunt.infant.data$class_rate)

\### Factor loadings ----

\## 1. Stack all 100 LD1 loadings into one tidy data frame

loadings_long <- bind_rows(

lapply(seq_along(pdfa.Grunts.Infants$scaling), function(i) {

data.frame(

variable = rownames(pdfa.Grunts.Infants$scaling[[i]]),

loading	= pdfa.Grunts.Infants$scaling[[i]][, "LD1"],

iteration = i

)

})

)

\## 2. Summarise loadings + stability across all pDFAs

grunts.inf_loadings_long <- loadings_long %>%

group_by(variable) %>%

summarise(

mean_loading	= mean(loading),

sd_loading	= sd(loading),

median_loading = median(loading),

ci_lower	= quantile(loading, 0.025),

ci_upper	= quantile(loading, 0.975),

\# STABILITY: proportion of runs with same sign as mean loading

prop_same_sign = mean(sign(loading) == sign(mean(loading))),

.groups = "drop"

) %>%

arrange(desc(abs(mean_loading)))

\## 3. View final clean table

grunts.inf_loadings_long

grunts.inf_loadings_long <- grunts.inf_loadings_long |>

mutate(

variable = recode(

variable,

"Avg_Entropy"	= "Average Entropy (Bits)",

"Element.rate"	= "Element rate (s-1)",

"Max_Entropy"	= "Maximum Entropy (Bits)",

"Avg_Power_Density"	= "Average Power Density (db FS)",

"Bout.duration..sec."	= "Bout duration (seconds)",

"PFC_Max_Slope"	= "Peak frequency contour max slope (Hz/ms)",

"Total.Elements.in.bout"	= "Total elements in bout",

"Freq_5."	= "Frequency 5% (Hz)",

"q3_freq"	= "Q3 frequency (Hz)",

"BW_50."	= "50% bandwidth (Hz)",

"PFC_Max_Freq"	= "Peak frequency contour maximum frequence (Hz)",

"Max_Freq"	= "Maximum frequency (Hz)",

"Delta_Freq"	= "Delta frequency (Hz)",

"BW_90."	= "90% bandwidth (Hz)"

))

grunts.inf_loadings_long <- grunts.inf_loadings_long |>

dplyr::rename(

"Variable"	= variable,

"Mean loading"	= mean_loading,

"Standard deviation loading"	= sd_loading,

"Median loading"	= median_loading,

"Lower confidence interval"	= ci_lower,

"Upper confidence interval"	= ci_upper,

"Proportion of the same sign"	= prop_same_sign

)

\## Model 1b: Grunts - Juveniles ----

#Filter for Grunts in juveniles

Grunts_Juveniles <- Acoustic_Data %>%

filter(Vocalisation == "Grunt") %>%

filter(`Age group` == "Juvenile")

Grunts_Juveniles_Age <- Grunts_Juveniles %>%

group_by(Site, ID_Individual, `Age group`) %>%

summarise(n=n())

n_distinct(Grunts_Juveniles$ID_Individual[Grunts_Juveniles$Site == "Chimfunshi"])

n_distinct(Grunts_Juveniles$ID_Individual[Grunts_Juveniles$Site == "Gombe"])

write.table(Grunts_Juveniles, file = "pdfa.grunt.juv.data.txt", sep = "\t", row.names = FALSE)

pdfa.grunt.juv.data=read.table(file="pdfa.grunt.juv.data.txt",

header=T, sep="\t", fill=T, dec=".")

summary(pdfa.grunt.juv.data)

names(pdfa.grunt.juv.data) # for correct variable naming

\# > names(pdfa.grunt.juv.data) # for correct variable naming

\# [1] "row_id"	"ID_Bout"	"ID_Individual"	"Site"

\# [5] "Age.group"	"Vocalisation"	"Total.Elements.in.bout" "Bout.duration..sec."

\# [9] "Element.rate"	"Delta_Freq"	"q3_freq"	"Avg_Entropy"

\# [13] "BW_90."	"Avg_Power_Density"	"BW_50."	"Freq_5."

\# [17] "Max_Entropy"	"Max_Freq"	"PFC_Max_Freq"	"PFC_Max_Slope"

\# [21] "PFC_Num_Inf_Pts"

pdfa.Grunts.Juveniles=pDFA.nested(test.fac="Site",

contr.fac="ID_Individual",

variables=c("Total.Elements.in.bout", "Bout.duration..sec.",

"Element.rate", "Delta_Freq",

"q3_freq", "Avg_Entropy", "BW_90.", "Avg_Power_Density",

"BW_50.", "Freq_5.", "Max_Entropy", "Max_Freq",

"PFC_Max_Freq", "PFC_Max_Slope"),

restrict.by = NULL,

n.contr.fac.levels.to.sel= NULL,

n.to.sel.per.contr.fac.level=NULL, n.sel=100, n.perm=1000,

pdfa.data=pdfa.grunt.juv.data)

Grunts.Juvenile_Result <- as.data.frame(pdfa.Grunts.Juveniles$result)

Grunts.Juvenile_Result

\# recalculates the data point after dropping it - percentage of how often that data point is classified correctly

pdfa.grunt.juv.data$class_rate <- pdfa.Grunts.Juveniles$correct.rate

\# use this variable for model - non-binary model - use a beta/gamma distribution (beta probably)

\# start with mixed model - add random effect for individual

\# predict class.rate according to age group (juvenile vs infant)

\# - if it turns out tricky, do a simple model (linear model) procedures account for non-independence in pdfa

mean(pdfa.grunt.juv.data$class_rate)

pdfa.Grunts.Juveniles$result["percent.corr.cross.classified"] / 100

hist(pdfa.grunt.juv.data$class_rate)

\### Factor loadings ----

\## 1. Stack all 100 LD1 loadings into one tidy data frame

loadings_long <- bind_rows(

lapply(seq_along(pdfa.Grunts.Juveniles$scaling), function(i) {

data.frame(

variable = rownames(pdfa.Grunts.Juveniles$scaling[[i]]),

loading	= pdfa.Grunts.Juveniles$scaling[[i]][, "LD1"],

iteration = i

)

})

)

\## 2. Summarise loadings + stability across all pDFAs

grunts.juv_loadings_long <- loadings_long %>%

group_by(variable) %>%

summarise(

mean_loading	= mean(loading),

sd_loading	= sd(loading),

median_loading = median(loading),

ci_lower	= quantile(loading, 0.025),

ci_upper	= quantile(loading, 0.975),

\# STABILITY: proportion of runs with same sign as mean loading

prop_same_sign = mean(sign(loading) == sign(mean(loading))),

.groups = "drop"

) %>%

arrange(desc(abs(mean_loading)))

\## 3. View final clean table

grunts.juv_loadings_long

grunts.juv_loadings_long <- grunts.juv_loadings_long |>

mutate(

variable = recode(

variable,

"Avg_Entropy"	= "Average Entropy (Bits)",

"Element.rate"	= "Element rate (s-1)",

"Max_Entropy"	= "Maximum Entropy (Bits)",

"Avg_Power_Density"	= "Average Power Density (db FS)",

"Bout.duration..sec."	= "Bout duration (seconds)",

"PFC_Max_Slope"	= "Peak frequency contour max slope (Hz/ms)",

"Total.Elements.in.bout"	= "Total elements in bout",

"Freq_5."	= "Frequency 5% (Hz)",

"q3_freq"	= "Q3 frequency (Hz)",

"BW_50."	= "50% bandwidth (Hz)",

"PFC_Max_Freq"	= "Peak frequency contour maximum frequence (Hz)",

"Max_Freq"	= "Maximum frequency (Hz)",

"Delta_Freq"	= "Delta frequency (Hz)",

"BW_90."	= "90% bandwidth (Hz)"

))

grunts.juv_loadings_long <- grunts.juv_loadings_long |>

dplyr::rename(

"Variable"	= variable,

"Mean loading"	= mean_loading,

"Standard deviation loading"	= sd_loading,

"Median loading"	= median_loading,

"Lower confidence interval"	= ci_lower,

"Upper confidence interval"	= ci_upper,

"Proportion of the same sign"	= prop_same_sign

)

\## Model 2a: Whimpers - Infants ----

#Filter for Whimpers in infants

Whimpers_Infants <- Acoustic_Data %>%

filter(Vocalisation == "Whimper") %>%

filter(`Age group` == "Infant")

Whimpers_Infants_Age <- Whimpers_Infants %>%

group_by(Site, ID_Individual, `Age group`) %>%

summarise(n=n())

n_distinct(Whimpers_Infants$ID_Individual[Whimpers_Infants$Site == "Chimfunshi"])

n_distinct(Whimpers_Infants$ID_Individual[Whimpers_Infants$Site == "Gombe"])

write.table(Whimpers_Infants, file = "pdfa.whimp.infant.data.txt", sep = "\t", row.names = FALSE)

pdfa.whimp.infant.data=read.table(file="pdfa.whimp.infant.data.txt",

header=T, sep="\t", fill=T, dec=".")

summary(pdfa.whimp.infant.data)

names(pdfa.whimp.infant.data) # for correct variable naming

\# > names(pdfa.whimp.infant.data) # for correct variable naming

\# [1] "row_id"	"ID_Bout"	"ID_Individual"	"Site"

\# [5] "Age.group"	"Vocalisation"	"Total.Elements.in.bout" "Bout.duration..sec."

\# [9] "Element.rate"	"Delta_Freq"	"q3_freq"	"Avg_Entropy"

\# [13] "BW_90."	"Avg_Power_Density"	"BW_50."	"Freq_5."

\# [17] "Max_Entropy"	"Max_Freq"	"PFC_Max_Freq"	"PFC_Max_Slope"

\# [21] "PFC_Num_Inf_Pts"

pdfa.Whimpers.Infants=pDFA.nested(test.fac="Site",

contr.fac="ID_Individual",

variables=c("Total.Elements.in.bout", "Bout.duration..sec.",

"Element.rate", "Delta_Freq",

"q3_freq", "Avg_Entropy", "BW_90.", "Avg_Power_Density",

"BW_50.", "Freq_5.", "Max_Entropy", "Max_Freq",

"PFC_Max_Freq", "PFC_Max_Slope"),

restrict.by = NULL,

n.contr.fac.levels.to.sel= NULL,

n.to.sel.per.contr.fac.level=NULL, n.sel=100, n.perm=1000,

pdfa.data=pdfa.whimp.infant.data)

Whimpers.Infants_Result <- as.data.frame(pdfa.Whimpers.Infants$result)

Whimpers.Infants_Result

\# recalculates the data point after dropping it - percentage of how often that data point is classified correctly

pdfa.whimp.infant.data$class_rate <- pdfa.Whimpers.Infants$correct.rate

\# use this variable for model - non-binary model - use a beta/gamma distribution (beta probably)

\# start with mixed model - add random effect for individual

\# predict class.rate according to age group (juvenile vs infant)

\# - if it turns out tricky, do a simple model (linear model) procedures account for non-independence in pdfa

mean(pdfa.whimp.infant.data$class_rate)

pdfa.Whimpers.Infants$result["percent.corr.cross.classified"] / 100

hist(pdfa.whimp.infant.data$class_rate)

\### Factor loadings ----

\## 1. Stack all 100 LD1 loadings into one tidy data frame

loadings_long <- bind_rows(

lapply(seq_along(pdfa.Whimpers.Infants$scaling), function(i) {

data.frame(

variable = rownames(pdfa.Whimpers.Infants$scaling[[i]]),

loading	= pdfa.Whimpers.Infants$scaling[[i]][, "LD1"],

iteration = i

)

})

)

\## 2. Summarise loadings + stability across all pDFAs

whimp.inf_loadings_long <- loadings_long %>%

group_by(variable) %>%

summarise(

mean_loading	= mean(loading),

sd_loading	= sd(loading),

median_loading = median(loading),

ci_lower	= quantile(loading, 0.025),

ci_upper	= quantile(loading, 0.975),

\# STABILITY: proportion of runs with same sign as mean loading

prop_same_sign = mean(sign(loading) == sign(mean(loading))),

.groups = "drop"

) %>%

arrange(desc(abs(mean_loading)))

\## 3. View final clean table

whimp.inf_loadings_long

whimp.inf_loadings_long <- whimp.inf_loadings_long |>

mutate(

variable = recode(

variable,

"Avg_Entropy"	= "Average Entropy (Bits)",

"Element.rate"	= "Element rate (s-1)",

"Max_Entropy"	= "Maximum Entropy (Bits)",

"Avg_Power_Density"	= "Average Power Density (db FS)",

"Bout.duration..sec."	= "Bout duration (seconds)",

"PFC_Max_Slope"	= "Peak frequency contour max slope (Hz/ms)",

"Total.Elements.in.bout"	= "Total elements in bout",

"Freq_5."	= "Frequency 5% (Hz)",

"q3_freq"	= "Q3 frequency (Hz)",

"BW_50."	= "50% bandwidth (Hz)",

"PFC_Max_Freq"	= "Peak frequency contour maximum frequence (Hz)",

"Max_Freq"	= "Maximum frequency (Hz)",

"Delta_Freq"	= "Delta frequency (Hz)",

"BW_90."	= "90% bandwidth (Hz)"

))

whimp.inf_loadings_long <- whimp.inf_loadings_long |>

dplyr::rename(

"Variable"	= variable,

"Mean loading"	= mean_loading,

"Standard deviation loading"	= sd_loading,

"Median loading"	= median_loading,

"Lower confidence interval"	= ci_lower,

"Upper confidence interval"	= ci_upper,

"Proportion of the same sign"	= prop_same_sign

)

\## Model 2b: Whimpers - Juveniles ----

#Filter for Whimpers in juveniles

Whimpers_Juveniles <- Acoustic_Data %>%

filter(Vocalisation == "Whimper") %>%

filter(`Age group` == "Juvenile")

Whimpers_Juveniles_Age <- Whimpers_Juveniles %>%

group_by(Site, ID_Individual, `Age group`) %>%

summarise(n=n())

n_distinct(Whimpers_Juveniles$ID_Individual[Whimpers_Juveniles$Site == "Chimfunshi"])

n_distinct(Whimpers_Juveniles$ID_Individual[Whimpers_Juveniles$Site == "Gombe"])

write.table(Whimpers_Juveniles, file = "pdfa.whimp.juv.data.txt", sep = "\t", row.names = FALSE)

pdfa.whimp.juv.data=read.table(file="pdfa.whimp.juv.data.txt",

header=T, sep="\t", fill=T, dec=".")

summary(pdfa.whimp.juv.data)

names(pdfa.whimp.juv.data) # for correct variable naming

\#

\# > names(pdfa.whimp.juv.data) # for correct variable naming

\# [1] "row_id"	"ID_Bout"	"ID_Individual"	"Site"

\# [5] "Age.group"	"Vocalisation"	"Total.Elements.in.bout" "Bout.duration..sec."

\# [9] "Element.rate"	"Delta_Freq"	"q3_freq"	"Avg_Entropy"

\# [13] "BW_90."	"Avg_Power_Density"	"BW_50."	"Freq_5."

\# [17] "Max_Entropy"	"Max_Freq"	"PFC_Max_Freq"	"PFC_Max_Slope"

\# [21] "PFC_Num_Inf_Pts"

pdfa.Whimpers.Juveniles=pDFA.nested(test.fac="Site",

contr.fac="ID_Individual",

variables=c("Total.Elements.in.bout", "Bout.duration..sec.",

"Element.rate", "Delta_Freq",

"q3_freq", "Avg_Entropy", "BW_90.", "Avg_Power_Density",

"BW_50.", "Freq_5.", "Max_Entropy", "Max_Freq",

"PFC_Max_Freq", "PFC_Max_Slope"),

restrict.by = NULL,

n.contr.fac.levels.to.sel= NULL,

n.to.sel.per.contr.fac.level=NULL, n.sel=100, n.perm=1000,

pdfa.data=pdfa.whimp.juv.data)

Whimpers.Juveniles_Result <- as.data.frame(pdfa.Whimpers.Juveniles$result)

Whimpers.Juveniles_Result

\# recalculates the data point after dropping it - percentage of how often that data point is classified correctly

pdfa.whimp.juv.data$class_rate <- pdfa.Whimpers.Juveniles$correct.rate

\# use this variable for model - non-binary model - use a beta/gamma distribution (beta probably)

\# start with mixed model - add random effect for individual

\# predict class.rate according to age group (juvenile vs infant)

\# - if it turns out tricky, do a simple model (linear model) procedures account for non-independence in pdfa

mean(pdfa.whimp.juv.data$class_rate)

pdfa.Whimpers.Juveniles$result["percent.corr.cross.classified"] / 100

hist(pdfa.whimp.juv.data$class_rate)

\### Factor loadings ----

\## 1. Stack all 100 LD1 loadings into one tidy data frame

loadings_long <- bind_rows(

lapply(seq_along(pdfa.Whimpers.Juveniles$scaling), function(i) {

data.frame(

variable = rownames(pdfa.Whimpers.Juveniles$scaling[[i]]),

loading	= pdfa.Whimpers.Juveniles$scaling[[i]][, "LD1"],

iteration = i

)

})

)

\## 2. Summarise loadings + stability across all pDFAs

whimp.Juv_loadings_long <- loadings_long %>%

group_by(variable) %>%

summarise(

mean_loading	= mean(loading),

sd_loading	= sd(loading),

median_loading = median(loading),

ci_lower	= quantile(loading, 0.025),

ci_upper	= quantile(loading, 0.975),

\# STABILITY: proportion of runs with same sign as mean loading

prop_same_sign = mean(sign(loading) == sign(mean(loading))),

.groups = "drop"

) %>%

arrange(desc(abs(mean_loading)))

\## 3. View final clean table

whimp.Juv_loadings_long

whimp.Juv_loadings_long <- whimp.Juv_loadings_long |>

mutate(

variable = recode(

variable,

"Avg_Entropy"	= "Average Entropy (Bits)",

"Element.rate"	= "Element rate (s-1)",

"Max_Entropy"	= "Maximum Entropy (Bits)",

"Avg_Power_Density"	= "Average Power Density (db FS)",

"Bout.duration..sec."	= "Bout duration (seconds)",

"PFC_Max_Slope"	= "Peak frequency contour max slope (Hz/ms)",

"Total.Elements.in.bout"	= "Total elements in bout",

"Freq_5."	= "Frequency 5% (Hz)",

"q3_freq"	= "Q3 frequency (Hz)",

"BW_50."	= "50% bandwidth (Hz)",

"PFC_Max_Freq"	= "Peak frequency contour maximum frequence (Hz)",

"Max_Freq"	= "Maximum frequency (Hz)",

"Delta_Freq"	= "Delta frequency (Hz)",

"BW_90."	= "90% bandwidth (Hz)"

))

whimp.Juv_loadings_long <- whimp.Juv_loadings_long |>

dplyr::rename(

"Variable"	= variable,

"Mean loading"	= mean_loading,

"Standard deviation loading"	= sd_loading,

"Median loading"	= median_loading,

"Lower confidence interval"	= ci_lower,

"Upper confidence interval"	= ci_upper,

"Proportion of the same sign"	= prop_same_sign

)

\## Model 3a: Laughs - Infants ----

#Filter for Laughs in infants

Laughs_Infants <- Acoustic_Data %>%

filter(Vocalisation == "Laugh") %>%

filter(`Age group` == "Infant")

Laughs_Infants_Age <- Laughs_Infants %>%

group_by(Site, ID_Individual, `Age group`) %>%

summarise(n=n())

n_distinct(Laughs_Infants$ID_Individual[Laughs_Infants$Site == "Chimfunshi"])

n_distinct(Laughs_Infants$ID_Individual[Laughs_Infants$Site == "Gombe"])

write.table(Laughs_Infants, file = "pdfa.laugh.infant.data.txt", sep = "\t", row.names = FALSE)

pdfa.laugh.infant.data=read.table(file="pdfa.laugh.infant.data.txt",

header=T, sep="\t", fill=T, dec=".")

summary(pdfa.laugh.infant.data)

names(pdfa.laugh.infant.data) # for correct variable naming

\# > names(pdfa.laugh.infant.data) # for correct variable naming

\# [1] "row_id"	"ID_Bout"	"ID_Individual"	"Site"

\# [5] "Age.group"	"Vocalisation"	"Total.Elements.in.bout" "Bout.duration..sec."

\# [9] "Element.rate"	"Delta_Freq"	"q3_freq"	"Avg_Entropy"

\# [13] "BW_90."	"Avg_Power_Density"	"BW_50."	"Freq_5."

\# [17] "Max_Entropy"	"Max_Freq"	"PFC_Max_Freq"	"PFC_Max_Slope"

\# [21] "PFC_Num_Inf_Pts"

pdfa.Laughs.Infants=pDFA.nested(test.fac="Site",

contr.fac="ID_Individual",

variables=c("Total.Elements.in.bout", "Bout.duration..sec.",

"Element.rate", "Delta_Freq",

"q3_freq", "Avg_Entropy", "BW_90.", "Avg_Power_Density",

"BW_50.", "Freq_5.", "Max_Entropy", "Max_Freq",

"PFC_Max_Freq", "PFC_Max_Slope"),

restrict.by = NULL,

n.contr.fac.levels.to.sel= NULL,

n.to.sel.per.contr.fac.level=NULL, n.sel=100, n.perm=1000,

pdfa.data=pdfa.laugh.infant.data)

Laughs.Infants_Result <- as.data.frame(pdfa.Laughs.Infants$result)

Laughs.Infants_Result

\# recalculates the data point after dropping it - percentage of how often that data point is classified correctly

pdfa.laugh.infant.data$class_rate <- pdfa.Laughs.Infants$correct.rate

\# use this variable for model - non-binary model - use a beta/gamma distribution (beta probably)

\# start with mixed model - add random effect for individual

\# predict class.rate according to age group (juvenile vs infant)

\# - if it turns out tricky, do a simple model (linear model) procedures account for non-independence in pdfa

mean(pdfa.laugh.infant.data$class_rate)

pdfa.Laughs.Infants$result["percent.corr.cross.classified"] / 100

hist(pdfa.laugh.infant.data$class_rate)

\### Factor loadings ----

\## 1. Stack all 100 LD1 loadings into one tidy data frame

loadings_long <- bind_rows(

lapply(seq_along(pdfa.Laughs.Infants$scaling), function(i) {

data.frame(

variable = rownames(pdfa.Laughs.Infants$scaling[[i]]),

loading	= pdfa.Laughs.Infants$scaling[[i]][, "LD1"],

iteration = i

)

})

)

\## 2. Summarise loadings + stability across all pDFAs

laughs.Inf_loadings_long <- loadings_long %>%

group_by(variable) %>%

summarise(

mean_loading	= mean(loading),

sd_loading	= sd(loading),

median_loading = median(loading),

ci_lower	= quantile(loading, 0.025),

ci_upper	= quantile(loading, 0.975),

\# STABILITY: proportion of runs with same sign as mean loading

prop_same_sign = mean(sign(loading) == sign(mean(loading))),

.groups = "drop"

) %>%

arrange(desc(abs(mean_loading)))

\## 3. View final clean table

laughs.Inf_loadings_long

laughs.Inf_loadings_long <- laughs.Inf_loadings_long |>

mutate(

variable = recode(

variable,

"Avg_Entropy"	= "Average Entropy (Bits)",

"Element.rate"	= "Element rate (s-1)",

"Max_Entropy"	= "Maximum Entropy (Bits)",

"Avg_Power_Density"	= "Average Power Density (db FS)",

"Bout.duration..sec."	= "Bout duration (seconds)",

"PFC_Max_Slope"	= "Peak frequency contour max slope (Hz/ms)",

"Total.Elements.in.bout"	= "Total elements in bout",

"Freq_5."	= "Frequency 5% (Hz)",

"q3_freq"	= "Q3 frequency (Hz)",

"BW_50."	= "50% bandwidth (Hz)",

"PFC_Max_Freq"	= "Peak frequency contour maximum frequence (Hz)",

"Max_Freq"	= "Maximum frequency (Hz)",

"Delta_Freq"	= "Delta frequency (Hz)",

"BW_90."	= "90% bandwidth (Hz)"

))

laughs.Inf_loadings_long <- laughs.Inf_loadings_long |>

dplyr::rename(

"Variable"	= variable,

"Mean loading"	= mean_loading,

"Standard deviation loading"	= sd_loading,

"Median loading"	= median_loading,

"Lower confidence interval"	= ci_lower,

"Upper confidence interval"	= ci_upper,

"Proportion of the same sign"	= prop_same_sign

)

\## Model 3b: Laughs - Juveniles ----

Laughs_Juveniles <- Acoustic_Data %>%

filter(Vocalisation == "Laugh") %>%

filter(`Age group` == "Juvenile")

Laughs_Juveniles_Age <- Laughs_Juveniles %>%

group_by(Site, ID_Individual, `Age group`) %>%

summarise(n=n())

n_distinct(Laughs_Juveniles$ID_Individual[Laughs_Juveniles$Site == "Chimfunshi"])

n_distinct(Laughs_Juveniles$ID_Individual[Laughs_Juveniles$Site == "Gombe"])

write.table(Laughs_Juveniles, file = "pdfa.laugh.juv.data.txt", sep = "\t", row.names = FALSE)

pdfa.laugh.juv.data=read.table(file="pdfa.laugh.juv.data.txt",

header=T, sep="\t", fill=T, dec=".")

summary(pdfa.laugh.juv.data)

names(pdfa.laugh.juv.data) # for correct variable naming

\# > names(pdfa.laugh.juv.data) # for correct variable naming

\# [1] "row_id"	"ID_Bout"	"ID_Individual"	"Site"

\# [5] "Age.group"	"Vocalisation"	"Total.Elements.in.bout" "Bout.duration..sec."

\# [9] "Element.rate"	"Delta_Freq"	"q3_freq"	"Avg_Entropy"

\# [13] "BW_90."	"Avg_Power_Density"	"BW_50."	"Freq_5."

\# [17] "Max_Entropy"	"Max_Freq"	"PFC_Max_Freq"	"PFC_Max_Slope"

\# [21] "PFC_Num_Inf_Pts"

pdfa.Laughs.Juveniles=pDFA.nested(test.fac="Site",

contr.fac="ID_Individual",

variables=c("Total.Elements.in.bout", "Bout.duration..sec.",

"Element.rate", "Delta_Freq",

"q3_freq", "Avg_Entropy", "BW_90.", "Avg_Power_Density",

"BW_50.", "Freq_5.", "Max_Entropy", "Max_Freq",

"PFC_Max_Freq", "PFC_Max_Slope"),

restrict.by = NULL,

n.contr.fac.levels.to.sel= NULL,

n.to.sel.per.contr.fac.level=NULL, n.sel=100, n.perm=1000,

pdfa.data=pdfa.laugh.juv.data)

Laughs.Juv_Result <- as.data.frame(pdfa.Laughs.Juveniles$result)

Laughs.Juv_Result

\# recalculates the data point after dropping it - percentage of how often that data point is classified correctly

pdfa.laugh.juv.data$class_rate <- pdfa.Laughs.Juveniles$correct.rate

\# use this variable for model - non-binary model - use a beta/gamma distribution (beta probably)

\# start with mixed model - add random effect for individual

\# predict class.rate according to age group (juvenile vs infant)

\# - if it turns out tricky, do a simple model (linear model) procedures account for non-independence in pdfa

mean(pdfa.laugh.juv.data$class_rate)

pdfa.Laughs.Juveniles$result["percent.corr.cross.classified"] / 100

hist(pdfa.laugh.juv.data$class_rate)

\### Factor loadings ----

\## 1. Stack all 100 LD1 loadings into one tidy data frame

loadings_long <- bind_rows(

lapply(seq_along(pdfa.Laughs.Juveniles$scaling), function(i) {

data.frame(

variable = rownames(pdfa.Laughs.Juveniles$scaling[[i]]),

loading	= pdfa.Laughs.Juveniles$scaling[[i]][, "LD1"],

iteration = i

)

})

)

\## 2. Summarise loadings + stability across all pDFAs

laughs.Juv_loadings_long <- loadings_long %>%

group_by(variable) %>%

summarise(

mean_loading	= mean(loading),

sd_loading	= sd(loading),

median_loading = median(loading),

ci_lower	= quantile(loading, 0.025),

ci_upper	= quantile(loading, 0.975),

\# STABILITY: proportion of runs with same sign as mean loading

prop_same_sign = mean(sign(loading) == sign(mean(loading))),

.groups = "drop"

) %>%

arrange(desc(abs(mean_loading)))

\## 3. View final clean table

laughs.Juv_loadings_long

laughs.Juv_loadings_long <- laughs.Juv_loadings_long |>

mutate(

variable = recode(

variable,

"Avg_Entropy"	= "Average Entropy (Bits)",

"Element.rate"	= "Element rate (s-1)",

"Max_Entropy"	= "Maximum Entropy (Bits)",

"Avg_Power_Density"	= "Average Power Density (db FS)",

"Bout.duration..sec."	= "Bout duration (seconds)",

"PFC_Max_Slope"	= "Peak frequency contour max slope (Hz/ms)",

"Total.Elements.in.bout"	= "Total elements in bout",

"Freq_5."	= "Frequency 5% (Hz)",

"q3_freq"	= "Q3 frequency (Hz)",

"BW_50."	= "50% bandwidth (Hz)",

"PFC_Max_Freq"	= "Peak frequency contour maximum frequence (Hz)",

"Max_Freq"	= "Maximum frequency (Hz)",

"Delta_Freq"	= "Delta frequency (Hz)",

"BW_90."	= "90% bandwidth (Hz)"

))

laughs.Juv_loadings_long <- laughs.Juv_loadings_long |>

dplyr::rename(

"Variable"	= variable,

"Mean loading"	= mean_loading,

"Standard deviation loading"	= sd_loading,

"Median loading"	= median_loading,

"Lower confidence interval"	= ci_lower,

"Upper confidence interval"	= ci_upper,

"Proportion of the same sign"	= prop_same_sign

)

\# 4. Zero-one inflated beta regression for classification comparisons----

\# Outcome: class_rate - the rate of which each data point was classified correctly

\# Predictor: age_group - Juvenile or Infant (2 categories) - does the age group influence the rate of classification

\# Random effect: id_bout - Data points are repeated over the same bout, Individual is accounted for in pdfa

\## Grunts ----

\### Group data ----

\# Fuse dataframes for each age group together

class_rate_grunts <- rbind(

pdfa.grunt.infant.data[, c("row_id", "class_rate")],

pdfa.grunt.juv.data[, c("row_id", "class_rate")]

)

class.grunts <- merge(

Grunts,

class_rate_grunts,

by = "row_id",

all.x = TRUE

)

names(class.grunts)

library(janitor)

class.grunts <- clean_names(class.grunts)

hist(class.grunts$class_rate)

\### Run model ----

library(glmmTMB)

library(DHARMa)

\# Boundary adjustment as beta cannot run with 0s and 1s

\# Squeeze values slightly inside 0 & 1

eps <- 0.0001

class.grunts$class_rate_beta <- pmin(

pmax(class.grunts$class_rate, eps),

1 - eps

)

\# Run model

model.grunts <- glmmTMB(

class_rate_beta ~ age_group + (1 | id_bout),

family = beta_family(link = "logit"),

data = class.grunts

)

summary(model.grunts)

res <- simulateResiduals(fittedModel = model.grunts)

plot(res)

\### Calculate predictions ----

tidy_beta_predictions <- function(model) {

age_groups <- c("Infant", "Juvenile")

fe <- fixef(model)$cond

vcov_mat <- vcov(model)$cond

logit_values <- c(

Infant	= fe["(Intercept)"],

Juvenile = fe["(Intercept)"] + fe["age_groupJuvenile"]

)

se_logit <- c(

Infant	= sqrt(vcov_mat["(Intercept)","(Intercept)"]),

Juvenile = sqrt(vcov_mat["(Intercept)","(Intercept)"] +

vcov_mat["age_groupJuvenile","age_groupJuvenile"] +

2*vcov_mat["(Intercept)","age_groupJuvenile"])

)

z <- qnorm(0.975)

logit_ci <- t(sapply(1:2, function(i) {

lower <- logit_values[i] - z * se_logit[i]

upper <- logit_values[i] + z * se_logit[i]

c(lower, upper)

}))

pred_prob <- exp(logit_values) / (1 + exp(logit_values))

ci_prob <- exp(logit_ci) / (1 + exp(logit_ci))

data.frame(

age_group = age_groups,

predicted_class_rate = round(pred_prob, 3),

ci_lower = round(ci_prob[,1], 3),

ci_upper = round(ci_prob[,2], 3)

)

}

pred_grunts <- tidy_beta_predictions(model.grunts)

pred_grunts

\### Plot ----

library(ggplot2)

\# Plot predicted classification rates with 95% CI

ggplot(pred_grunts, aes(x = age_group, y = predicted_class_rate)) +

geom_point(size = 3, color = "steelblue") +	# predicted points

geom_errorbar(aes(ymin = ci_lower, ymax = ci_upper), # confidence intervals

width = 0.2, color = "steelblue") +

ylim(0, 1) +	# class_rate is between 0 and 1

labs(

x = "Age Group",

y = "Predicted Classification Rate",

title = "Predicted Classification Rate of Grunts by Age Group"

) +

theme_minimal(base_size = 14)

\## Whimpers ----

\### Group data ----

\# Fuse dataframes for each age group together

class_rate_whimp <- rbind(

pdfa.whimp.infant.data[, c("row_id", "class_rate")],

pdfa.whimp.juv.data[, c("row_id", "class_rate")]

)

class.whimp <- merge(

Whimpers,

class_rate_whimp,

by = "row_id",

all.x = TRUE

)

class.whimp <- clean_names(class.whimp)

hist(class.whimp$class_rate)

\# Boundary adjustment as beta cannot run with 0s and 1s

\# Squeeze values slightly inside 0 & 1

eps <- 0.0001

class.whimp$class_rate_beta <- pmin(

pmax(class.whimp$class_rate, eps),

1 - eps

)

\### Run model ----

model.whimp <- glmmTMB(

class_rate_beta ~ age_group + (1 | id_bout),

family = beta_family(link = "logit"),

data = class.whimp

)

summary(model.whimp)

res <- simulateResiduals(fittedModel = model.whimp)

plot(res)

\### Calculate predictions ----

pred_whimp <- tidy_beta_predictions(model.whimp)

pred_whimp

\### Plot ----

\# Plot predicted classification rates with 95% CI

ggplot(pred_whimp, aes(x = age_group, y = predicted_class_rate)) +

geom_point(size = 3, color = "steelblue") +	# predicted points

geom_errorbar(aes(ymin = ci_lower, ymax = ci_upper), # confidence intervals

width = 0.2, color = "steelblue") +

ylim(0, 1) +	# class_rate is between 0 and 1

labs(

x = "Age Group",

y = "Predicted Classification Rate",

title = "Predicted Classification Rate of Whimpers by Age Group"

) +

theme_minimal(base_size = 14)

\## Laughs ----

\### Group data ----

\# Fuse dataframes for each age group together

class_rate_laughs <- rbind(

pdfa.laugh.infant.data[, c("row_id", "class_rate")],

pdfa.laugh.juv.data[, c("row_id", "class_rate")]

)

class.laugh <- merge(

Laughs,

class_rate_laughs,

by = "row_id",

all.x = TRUE

)

class.laugh <- clean_names(class.laugh)

hist(class.laugh$class_rate)

\# Boundary adjustment as beta cannot run with 0s and 1s

\# Squeeze values slightly inside 0 & 1

eps <- 0.0001

class.laugh$class_rate_beta <- pmin(

pmax(class.laugh$class_rate, eps),

1 - eps

)

\### Run model ----

model.laugh <- glmmTMB(

class_rate_beta ~ age_group + (1 | id_bout),

family = beta_family(link = "logit"),

data = class.laugh

)

summary(model.laugh)

res <- simulateResiduals(fittedModel = model.laugh)

plot(res)

\### Calculate predictions ----

pred_laughs <- tidy_beta_predictions(model.laugh)

pred_laughs

\### Plot ----

ggplot(pred_laughs,

aes(x = age_group, y = predicted_class_rate)) +

geom_point(size = 4) +

geom_errorbar(

aes(ymin = ci_lower, ymax = ci_upper),

width = 0.15,

linewidth = 1

) +

scale_y_continuous(

limits = c(0.85, 1),

labels = scales::percent_format(accuracy = 1)

) +

labs(

title = "Predicted Classification Rate of Laughs by Age Group",

x = "Age Group",

y = "Predicted Classification Rate"

) +

theme_minimal(base_size = 14) +

theme(

plot.title = element_text(face = "bold", hjust = 0.5),

axis.title = element_text(face = "bold")

)

\# 5. Final plots ----

#Model plots

plot(model.grunts)

pred_whimp$vocalisation <- "Whimpers"

pred_laughs$vocalisation <- "Laughs"

pred_grunts$vocalisation <- "Grunts"

pred_all <- rbind(pred_whimp, pred_laughs, pred_grunts)

ggplot(pred_all, aes(x = age_group, y = predicted_class_rate,

color = vocalisation)) +

geom_point(

position = position_dodge(width = 0.4),

size = 3

) +

geom_errorbar(

aes(ymin = ci_lower, ymax = ci_upper),

width = 0.15,

linewidth = 1,

position = position_dodge(width = 0.4)

) +

scale_y_continuous(

limits = c(0.7, 1),

labels = scales::percent_format(accuracy = 1)

) +

labs(

title = "Predicted Classification Rates by Age Group and Vocalisation Type",

x = "Age Group",

y = "Predicted Classification Rate",

color = "Vocalisation"

) +

theme_minimal(base_size = 14) +

theme(

plot.title = element_text(face = "bold", hjust = 0.5),

axis.title = element_text(face = "bold")

)

ggplot(pred_all, aes(x = age_group, y = predicted_class_rate,

color = vocalisation)) +

geom_point(size = 3) +

geom_errorbar(

aes(ymin = ci_lower, ymax = ci_upper),

width = 0.15

) +

facet_wrap(~ vocalisation) +

scale_y_continuous(

limits = c(0.7, 1),

labels = scales::percent_format(accuracy = 1)

) +

labs(

title = "Predicted Classification Rates by Age Group",

x = "Age Group",

y = "Predicted Classification Rate"

) +

theme_minimal(base_size = 14) +

theme(

plot.title = element_text(face = "bold", hjust = 0.5),

legend.position = "none"

)
