# Supplementary material for: Ontogeny of dialects across chimpanzee vocalisations
Source: PLoS One. 2026 Sep 2;21(9):e0355313. doi: 10.1371/journal.pone.0355313 (PMC13537595; doi:10.1371/journal.pone.0355313)
Supplement: Supplementary file 15 [file pone.0355313.s016.docx]

**S16 Figure. Pearson correlation matrix between temporal variables showing no correlations between variables > 0.9.**
