# Supplementary material for: Ontogeny of dialects across chimpanzee vocalisations
Source: PLoS One. 2026 Sep 2;21(9):e0355313. doi: 10.1371/journal.pone.0355313 (PMC13537595; doi:10.1371/journal.pone.0355313)
Supplement: Supplementary file 16 [file pone.0355313.s018.docx]

**Ontogeny of dialects across chimpanzee vocalizations**

We would like to thank all the Reviewers and the Editor for their useful comments, which greatly helped us to improve our previous manuscript. Below, we discuss the Reviewers comments in detail. For convenience, we reproduce the Reviewers’ comments in italics (marked by “Reviewer comment x:”), followed by our response (marked by “Author response:”). We numbered Reviewers’ comments continuously in order to facilitate referring back to previous Reviewer comments and Author responses (points).

**Reviewer #1**

**Reviewer comment 1:** *The manuscript by Giragosian et al. analyzes infant and juvenile vocalizations of chimpanzees from two sites to test whether population-specific signatures exist and whether these signatures are stronger in juveniles than in infants. To address this question, the authors use a large dataset (N = 11225 calls) comprising three call types (grunts, whimpers, and laughs), from which they extract acoustic parameters and apply permuted discriminant function analyses to test for site-level differentiation. They report that the two sites can be discriminated for all call types, and that classification accuracy increases from infancy to the juvenile stage.

The dataset is rich, and this manuscript addresses a question of central importance for understanding chimpanzee vocal development and for informing broader debates on the evolution of vocal learning in both non-human primates and humans. However, several analytical decisions require clearer justification to assess the robustness of the findings, and additional contextualization would strengthen the interpretation. I recommend that the authors revisit their analytical decisions and justifications, and consider adding clearer explanations as well as complementary analyses or visualizations to make the underlying data patterns more transparent and the strength of the results easier for readers to evaluate.*

**Author response:** We thank the reviewer for their positive and constructive assessment of the manuscript and for highlighting the relevance of this work for understanding chimpanzee vocal development and vocal learning. We are grateful for their careful evaluation of our analytical approach and for their suggestions to improve transparency and interpretability.

In response, we have substantially revised the analyses and presentation. Most notably, we have adopted a new analytical framework to assess developmental changes in population-level discrimination, using generalized linear mixed models of correct classification rates rather than chi-square tests. This approach allows a more robust treatment of developmental trajectories while accounting for repeated measures and individual-level variation.

In addition, we now explicitly incorporate and interpret factor loadings from the discriminant function analyses. This enables us to identify which acoustic parameters drive population differentiation and to link these differences more directly to biologically and ecologically meaningful processes, rather than relying solely on classification accuracy.

We believe these changes significantly strengthen the robustness of the analyses and provide a clearer, more mechanistic interpretation of why and how populations differ across development. We thank the reviewer for comments that directly motivated these improvements.

**Reviewer comment 2:** *The authors use the term "accents" and, in lines 44–45, distinguish accents from dialects in the context of human speech. However, in the non-human vocal-learning literature, dialect is far more commonly used to describe population-level variation in vocal production (e.g., Janik & Slater 1997; Nowicki & Searcy 2014; Desai et al. 2022). Therefore, it is not clear why the authors are using accents rather than dialects in the context of non-human primates. I believe the use of this term requires further justification and would greatly benefit from references to other studies on vocal convergence of non-human primates (often described as vocal accommodation) and/or other vocal-learning species in which the term "accent" has been used instead of "dialect".*

**Author response:** We thank the reviewer for highlighting this issue. Our decision to use the term accent over dialect was that as we understood, in humans, dialects are cases in which different words are used to communicate the same things, whereas accents are cases in which the same word in pronounced differently. Since we were looking at how the same call is constructed acoustically in different age groups and populations, we opted for the latter terminology. However, we acknowledge that this is not the conventional use of the term in the animal communication literature, as demonstrated by the provided references. Since addressing the conceptual inconsistencies in the use of the terms accent and dialect in the communication literature more generally is beyond the scope of our paper, we have decided to change our use of the term ‘accent’ and instead have used the term ‘dialect’.

**Reviewer comment 3:** *Line 237: Why is a chi-squared test on call-level "correct vs. incorrect" classifications appropriate? Because multiple calls come from the same individuals, the observations are not independent, which violates a key assumption of the chi-squared test and may lead to underestimated uncertainty and inflated significance. For this reason, I am not sure that a chi-squared test is the most appropriate approach here, and I would encourage either a method that accounts for repeated measures or a more detailed justification of why the chi-squared test remains valid despite this non-independence.*

**Author response:** We thank the reviewer for raising this important concern regarding non-independence of observations. We agree that a simple chi-squared test on call-level classifications does not adequately account for repeated measures and could lead to underestimated uncertainty.

In response, we have revised our analytical approach. First, the discriminant function analyses were conducted using a permuted DFA framework in which individual identity was controlled by restricting permutations within individuals, thereby preventing inflation of classification accuracy due to repeated sampling of the same callers.

Secondly, we have now replaced the chi-squared tests with beta regression mixed models to examine developmental differences in classification accuracy. These models treat classification rate as a proportional response variable and include bout ID as a random intercept, thereby directly accounting for repeated measures within bouts. This approach provides a more statistically appropriate framework for assessing age-related differences in discrimination accuracy.

We believe this revised analysis addresses the reviewer’s concern and substantially strengthens the robustness of our results.

**Reviewer comment 4:** *Why was age treated categorically rather than continuously? Given the range of ages and the fact that some individuals were recorded at multiple ages, modelling age as a continuous predictor could avoid arbitrary developmental boundaries and provide a more nuanced view of ontogenetic change. Could the authors clarify why this option was not pursued, and whether continuous-age analyses might be feasible with this dataset to offer additional insight?*

**Author response:** We thank the reviewer for raising this point. We agree that modelling age continuously would be interesting and informative. Unfortunately, however, there is insufficient data to run a pDFA model at each age (i.e., there are not individuals from all ages in each group, see supplementary table S1). Since our pDFA models have been performed at the age class level, and the aim of our developmental analysis is to understand the relationship between our pDFA models (i.e., to see whether there are differences in correct classification rates), in combination with limited data at certain ages, we have decided to use age class as a categorical variable in our mixed-models of classification rates also. We have clarified this in our methods section, and we feel it is a reasonable approach, given that chimpanzee life-history can be meaningfully divided into infant and juvenile periods (which are behaviourally, anatomically, and physiologically distinct periods in the life span), which are commonly used in many studies of chimpanzee ontogeny.

**Reviewer comment 5:** *Why was the contribution of specific acoustic features to the site discrimination not explored? Reporting the underlying DFA loadings or structure coefficients could provide useful insight into the acoustic basis of the differences, even if these results need to be interpreted cautiously due to the sample-size imbalance (or addressed through resampling to reduce this imbalance).*

**Author response:** We thank the reviewer for this valuable suggestion. We agree that examining the contribution of specific acoustic parameters to site discrimination provides important insight into the acoustic basis of the observed differences.

In response, we now report the factor loadings from all discriminant function analyses in the Results section and provide full loading summaries (including means, standard deviations, confidence intervals, and sign consistency across permutations) in the Supplementary Materials for all models. Because our DFA framework was implemented using a permutation procedure that controls for individual identity, we summarise loadings across permutations to provide a robust estimate of each parameter’s contribution while accounting for sample-size imbalance and repeated measures.

We have also revised the Discussion to explicitly interpret which acoustic domains (e.g., power density, temporal patterning, entropy measures) drive site-level discrimination and how their contributions change across development.

We believe these additions substantially clarify the acoustic basis of population differentiation and strengthen the transparency and interpretability of our findings.

**Reviewer comment 6:** *The interpretation of developmental changes should acknowledge other factors that may contribute to the observed patterns, including anatomical maturation and age-related differences in recording context (e.g., infants being held by their mothers).*

**Author response:** We thank the reviewer for highlighting additional factors that may contribute to the observed developmental changes in classification accuracy. We agree that anatomical maturation and age-related differences in recording context are important considerations.

In the revised Discussion, we now explicitly acknowledge the potential role of vocal tract growth and improving motor control in shaping call structure across development. We note that anatomical maturation may increase the stability and modulation capacity of vocalizations, potentially amplifying population-level differences that are only weakly expressed in infancy.

We also address the possibility that age-related differences in behavioural or recording context (e.g., infants being carried by their mothers or vocalizing at closer range) could influence acoustic properties or transmission characteristics. While our analyses focus on structural acoustic parameters rather than raw amplitude measures, and thus are unlikely to be solely explained by recording distance, we acknowledge that contextual factors may interact with developmental changes in vocal production.

These additions broaden the interpretation of our findings and clarify that ecological calibration, anatomical maturation, and socially mediated processes are not mutually exclusive explanations for the observed developmental strengthening of population signatures.

**Reviewer comment 7:**  *A brief discussion of the social contexts in which juveniles produce these calls would strengthen the interpretation. Do juveniles converge primarily with peers, adults, or through general exposure? Even speculative discussion of potential mechanisms (e.g., social feedback vs. eavesdropping) would be valuable.*

**Author response:** We thank the reviewer for this helpful suggestion. In the revised Discussion, we now include a brief consideration of the social contexts in which juveniles produce each call type and how these may contribute to developmental strengthening of population-level vocal signatures.

Specifically, we note that laughs are primarily produced during play and are most often directed toward peers, potentially providing opportunities for convergence through repeated social interaction and feedback within age cohorts. In contrast, whimpers are predominantly directed toward mothers, which may limit broader social shaping opportunities. Grunts show a developmental shift in social targeting: although infants grunt toward a range of partners, juveniles increasingly direct grunts toward higher-ranking individuals, similar to adults. Such changes in interaction partners may alter the available models for vocal exposure and influence the emergence of population-specific structure.

We also briefly outline potential mechanisms, including social feedback and passive exposure (eavesdropping), while acknowledging that our current dataset does not allow us to disentangle these processes. We believe this addition strengthens the interpretation of our developmental findings and clarifies the potential social pathways through which population signatures may emerge.

**Reviewer comment 8:** *The two sites differ substantially in their environmental characteristics. Are the acoustic differences reported consistent with predictions based on habitat structure (e.g., dry vs. wet, open vs. dense environments) and known effects on sound transmission? The interpretation would benefit from a discussion of how environmental conditions might shape the specific acoustic parameters analyzed and whether the observed site differences align with these expectations.*

**Author response:** We thank the reviewer for this insightful comment. In the revised Discussion, we now more explicitly situate the observed acoustic differences within predictions derived from habitat structure and known effects of environmental conditions on sound transmission.

Specifically, we highlight that the population inhabiting dense tropical rainforest (Gombe) consistently exhibited higher average power density than the population living in the more open miombo woodland (Chimfunshi). Increased signal energy is consistent with expectations for structurally complex habitats, where dense vegetation increases attenuation and scattering of sound and may favour signals with greater energy to enhance transmission.

In addition, we now expand our interpretation of temporal parameters that contributed to site discrimination, including element rate and total number of elements per bout. We discuss the possibility that increased repetition within call bouts may introduce redundancy, thereby enhancing detectability and robustness to degradation in acoustically challenging environments. Repeated elements can increase the likelihood that at least part of a signal is received clearly despite environmental masking and may allow receivers to integrate information across multiple acoustic events. Thus, habitat-related constraints may act not only on spectral energy (e.g., power density) but also on the temporal organization of calls.

We also clarify that, while the direction of the power density differences aligns with ecological predictions, not all acoustic parameters map straightforwardly onto habitat-based expectations, and multiple processes — including ecological calibration, anatomical maturation, and socially mediated influences — may interact to produce the observed population signatures. We believe these revisions strengthen the ecological framing of our findings while maintaining appropriate caution in interpretation.

**Reviewer comment 9:** *In line 233, please clarify what is meant by "restricting by developmental stage".*

**Author response:** We have now clarified that this means a separate model was done for each age class for each call type.

**Reviewer comment 10:** *It is not clear why the specific spectral and temporal features listed in Table 2 were chosen. For example, Desai et al. (2022) justified their selection by choosing acoustic features that were as similar as possible to those used in previous studies of adult chimpanzee dialects (Mitani et al., 1992, 1999; Crockford et al., 2004).*

**Author response:** We thank the reviewer for pointing this out. We have justified our choice based on the use of these parameters in a previous acoustic study of chimpanzee vocal ontogeny on the same population, which found meaningful acoustic differences in ontogeny.

**Reviewer comment 11:** *Line 75: The authors note that a recent study found that apparent population-specific vocal signatures could instead be explained by individual identity cues. Could the authors clarify how their analyses account for this possibility? For instance, what steps were taken to ensure that site-level differences are not confounded with individual-level vocal signatures?*

**Author response:** We thank the reviewer for raising this important point. We agree that apparent population-level vocal signatures can, in some cases, arise from individual identity cues if these are unevenly distributed across sites. We therefore took explicit steps to account for this possibility in our analyses.

First, we used a permuted discriminant function analysis (pDFA), which controls for non-independence by restricting permutations within levels of a control factor. In our case, individual identity was included as a control factor, such that randomizations were performed while holding individual identity constant. This approach tests whether site-level classification exceeds chance expectations after accounting for consistent individual-level acoustic variation.

Secondly, our beta regression analyses of discrimination performance, alongside inspection of factor loadings, indicate consistent site-level differentiation across multiple acoustic dimensions. These patterns are unlikely to arise purely from individual-level distinctiveness unless individuals within sites were more acoustically similar to one another than to individuals at the other site — which itself would constitute population-level structuring.

We have clarified these points in the revised manuscript to make explicit how individual identity was controlled and why our results are unlikely to be explained solely by individual vocal signatures.

**Reviewer comment 12:** *Please explain and justify the choices in reference to previous analyses.*

**Author response:** As explained above in response to comment 11, and following the procedure of Mundry and Sommer (2007), we control for individual identity by holding individual identity constant while randomizing group membership in the permutation procedure. We have included this explanation and justification in our statistical analysis section.

**Reviewer comment 13:** *Does the production frequency of the three call types (grunts, whimpers, laughs) change across development?*

**Author response:** There are very few developmental studies that look into vocal production rates per call type across the lifespan. From experience, we can say that laughs and whimpers become less common in adulthood and grunts become more common. To provide more context on call usage throughout development, we have included additional literature in our discussion.

**Reviewer comment 14:** *The authors briefly mention that the infant category spans from 6 months to 4 years of age, which may provide enough time for vocal learning to occur. It would be helpful to provide some reference to evaluate what is "enough time" for vocal learning.*

**Author response:** We thank the reviewer for suggesting that we elaborate on this point. We have included additional literature from both humans and chimpanzees, which shows that humans show signs of vocal learning as early as 9 months of age, and chimpanzee vocal learning, within the context of vocal sequences, begins at around 2-3 years of age, to better illustrate what could, in principle, be sufficient time for social impacts to influence call structure.

**Reviewer comment 15:** *Could the analytical framework used here—in principle—be applied to human accents? How do the acoustic features most important for distinguishing human accents compare to those analyzed in chimpanzees?*

**Author response:** We thank the reviewer for raising this interesting point. We have now included new references in lines 437-440 showing that many of the parameters that were key to encoding group identity also have been empirically shown to encode human accents.

**Reviewer comment 16:** *The analysis code, as well as the original data points behind the means and variance measures, should be made available for reproducibility.*

**Author response:** We apologise for failing to provide all materials needed for full reproducibility at first submission and have now included all data and code needed to reproduce our results.
