# Supplementary material for: Ontogeny of dialects across chimpanzee vocalisations
Source: PLoS One. 2026 Sep 2;21(9):e0355313. doi: 10.1371/journal.pone.0355313 (PMC13537595; doi:10.1371/journal.pone.0355313)
Supplement: Supplementary file 17 [file pone.0355313.s019.docx]

**Ontogeny of dialects across chimpanzee vocalizations**

We would like to thank all the Reviewers and the Editor for their useful comments, which greatly helped us to improve our previous manuscript. Below, we discuss the Reviewers comments in detail. For convenience, we reproduce the Reviewers’ comments in italics (marked by “Reviewer comment x:”), followed by our response (marked by “Author response:”). We numbered Reviewers’ comments continuously in order to facilitate referring back to previous Reviewer comments and Author responses (points).

**Review Comments to the Author**

**Reviewer #1:** *The authors did a good job addressing all my comments, and this new version of the manuscript is significantly more robust and informative regarding their methods and result interpretations. I still have two comments about clarifications that should be made about their analysis:*

**Reviewer comment 1**: Accounting for individual identity in the infancy/juvenile discrimination: In lines 244-250, the authors describe the model they used to test population-level classification accuracy between age groups. They accounted for the non-independence of calls produced within the same vocal bout, but there is nothing about individual identity. Calls can have correlated classification rates both by being in the same bout and by being from the same individual. The current model does not account for this, but it could be easily included by adding individual identity as a random intercept in the model. Additionally, for clarity, I believe the model equation should be stated in the methods section or the supporting material; it took me a while to find it in the script (class_rate_beta ~ age_group + (1 | id_bout)).

**Author response 1:** We agree with the reviewer that individual ID is an important source of variability that needs to be adequately controlled for. The pDFA package that we used allows you to control for grouping factors unrelated to your factor of interest. We have two relevant grouping factors: individual ID and bout ID. However, the software only allows you to control a single grouping factor. So, we decided to control for individual ID by holding it constant during the permutation process (as described in lines 231-235), and we controlled for bout ID by including this factor as a random intercept in our model. We have added an additional sentence in the data analysis section (lines 260-265) to clearly explain this. In addition, as the reviewer suggests, we have explicitly stated our model formula also.

**Reviewer comment 2:** *The authors should make it clear in the methods section that group coding was held constant across all 100 iterations, as this is a requirement to use the sign consistency metric. Additionally, please include an explicit statement of which population is the positive reference in the factor loading analysis.*

**Author response 2:** We thank the reviewer for pointing this out. We now clarify in lines 248-251 that we held the sign constant and specify which group was represented by + and which was represented by -.

**Reviewer #2:** Giragosian et al. Investigate the existence of dialects across two distinct chimpanzee populations and across different age classes, with the ultimate goal of providing insight into the ontogeny of accent development in the species. In general, I found the manuscript well-written, the methods used appropriate, the findings very interesting and mostly well discussed. However, there are some minor points that I think should be addressed before the manuscript is ready for publication.

**Reviewer comment 1:** *L.32, L.74, L.399: you should use furthermore instead of further.*

**Author response:** We thank the reviewer for the suggestion. We have made the edits suggested in lines 33, 77 and 401.

**Reviewer comment 2:** *L .58-59: in the brackets use e.g., as you do not cover the entirety of nonhuman primates for which cross-population variation has been shown. In addition, you are missing the closing bracket in the sentence.*

**Author response:** We thank the reviewer for the suggestion. We have made the edits suggested in lines 60-61.

**Reviewer comment 3:** *L.65: Use Nonetheless instead of Although.*

**Author response:** We thank the reviewer for the suggestion. We have made the edit suggested in line 65.

**Reviewer comment 4:** *L72-74: add references in the end of the sentence, to support your statement.*

**Author response:** We thank the reviewer for the suggestion. We have added references to support the claim in lines 75-76.

**Reviewer comment 5:** *L.118: it should be and/or*

**Author response:** We thank the reviewer for the suggestion. We have made the edit suggested in line 123.

**Reviewer comment 6*:*** *L.129: as you have 30 subjects, but one animal appears twice in your dataset because it represented in both ages classes (resulting in N=31), I would suggest writing “30 individual chimpanzees”. In addition, I would add in brackets how many animals in total were from Chimfushi Wildlife Orphanage and how many from Gombe National Park in L.129-130.*

**Author response:** We thank the reviewer for the suggestion. We have made the edits suggested in lines 134-135.

**Reviewer comment 7:** *L.134-135: I think you must have made a mistake in the calculation of the Mean. With the ages you have in S1 Table and with the span of the ages being between 4 and 10 you should not have a mean of 10.90.*

**Author response:** We thank the reviewer for the suggestion. We have made the edits suggested in lines 140.

**Reviewer comment 8:** *L.137-140: as you classified the animals in juvenile or infant based solely on age, I would remove the characteristics you list here, as they are not relevant for your study.*
**Author response:** We thank the reviewer for the suggestion. We have removed the characteristics described in lines 143.

**Reviewer comment 9:** *L.145, L.159: you should add information concerning the frequency sensitivity of the microphones and with which sampling frequency you conducted your recordings.*

**Author response 10:** We have added the sensitivity range for both devices, which was the same, in lines 148, 162-163 and 173-174. One set of recordings was analogue, so there is no sampling rate during recording, but we also now specify in the same section that both sets of recordings were digitised using the same sampling rate and bit-accuracy

**Reviewer comment 10:** *L.172-177: the coded unit in your study were not call types in general, but only the call types grunt, whimper and laughs. Therefore, I would suggest removing the first sentences and directly say that you only focused on the three call types whose definitions you provide, as they are the most predominant ones in infants and juveniles.*

**Author response:** We thank the reviewer for the suggestion. We have removed the first sentences of the paragraph as suggested, starting at line 179.

**Reviewer comment 11:** *Table 1: the references should be cited not only with the names of the authors and years of publication, but also be numbered, so one can directly find them in the references section of the publication.*

**Author response:** We thank the reviewer for the suggestion. We have numbered the citations in Table 1.

**Reviewer comment 12:** *L.186: 5 seconds after the offset or the onset of the previous call? Please add this information in the sentence.*

**Author response:** We thank the reviewer for the suggestion. We made the edit in line 187.

**Reviewer comment 13:** *L.194-195: were the calls recorded with this sampling rate or were they transformed before the analysis?*

**Author response 13:** This is the sampling rate set in Raven Pro after fast Fourier Transform of the signals, which we now specify in line 192.

**Reviewer comment 14:** *L.205: I find the threshold of 0.9 too high to reduce collinearity issues. Usually more conservative thresholds of 0.7 or 0.8 are used. How high were the correlations between your acoustic parameters and how can you justify the use of such a high threshold? Please add the correlations you calculated in the supplementary and justify in your manuscript the threshold you used.*

**Author response 14:** We thank the reviewer for this suggestion. We have now included the full pairwise correlation matrix in the Supplementary Material (Supplementary Figs. S1, S2, and S3). Pairwise Pearson correlation coefficients were calculated among all acoustic parameters, and highly correlated variables were identified using the findCorrelation() function in the R package **caret**, with a cutoff of r > 0.90. We chose this threshold to remove only variables that were nearly redundant while retaining descriptors that capture different aspects of call structure. Because many acoustic variables are inherently correlated, using a lower threshold (e.g. 0.7 or 0.8) would have resulted in the removal of variables that still provide biologically distinct information. We have clarified this rationale in the Methods in lines 205-218.

**Reviewer comment 15:** *L.206-207: how many acoustic parameters did you measure in total, how many and which ones did you remove due to collinearity issues and why did you choose the ones you chose? Please add this information in your manuscript.*

**Author response 15:** We have revised the Methods in lines 205-218 to specify the number of acoustic parameters included in the correlation analysis, the variables removed, and the procedure used for variable selection. Initially, 22 acoustic parameters were considered. Pairwise correlations were calculated among all variables, and variables with r > 0.90 were identified using findCorrelation() from the **caret** package. This function iteratively removes the variable with the largest mean absolute correlation among highly correlated pairs, thereby minimizing redundancy in the retained dataset. Following this procedure, 7 variables were removed (see supplementary figure S1, S2 and S3), leaving 15 variables for subsequent analyses.

**Reviewer comment 16:** *L.222: did you use the same number of calls from each population to have ~50% chance? The fact that you have two populations is not on its own sufficient to result in a 50% chance.*

**Author response 16:** Thank you for pointing this out. The expected classification is actually calculated across 1000 randomised permutations, and this gives the base rate to which the model performance is compared to, which is explained in the sentence that follows, and this part referring to a 50% chance has been removed in line 233.

**Reviewer comment 17:** *L.235: you have a small typo, it should be our and not out.*

**Author response:** We thank the reviewer for the suggestion. We have corrected the typo in line 245.
**Reviewer comment 18:** *L.251: a general remark about the Results section. I find your results in general nicely presented, but I find that you could use nicer introductory sentences throughout the results, to make the section easier to read and comprehend. For example, in L260 instead of “Population discrimination was significant” you could have something like “For infant grunts population discrimination was significant” or “For infant grunts there were significant differences between the two populations” or something like that. That is something that I would suggest doing for the introductory sentence of every single call type.*

**Author response:** We thank the reviewer for the suggestion. We have changed the introductory sentence for each call type in the results section as suggested throughout lines 268-384.

**Reviewer comment 19*:*** *L.390-395: this sentence is too long. I would suggest diving it in two sentences.*

**Author response:** We thank the reviewer for the suggestion. We have separated the sentence into two sentences in lines 389-394.

**Reviewer comment 20*:*** *L.392: you should add a reference for laughs*

**Author response:** We thank the reviewer for the suggestion. We have added a reference for laughs in line 391.

**Reviewer comment 21*:*** *L.402-415: I do not agree that the only comparison you bring into the discussion is with humans. There is extensive literature on many other nonhuman primates that you should integrate. For example, there is substantial work on vocal learning in marmosets during infancy that fully supports your arguments (e.g., Ghazanfar et al., 2026; Gultekin & Hage, 2017, 2018; Takahashi et al., 2017). In addition, differences in the vocalisations of different populations of primates have been described in mouse lemurs (e.g., Hafen et al.,1998), for which also vocal learning during adulthood and vocal plasticity during ontogeny have been suggested (Langehennig-Peristenidou et al. 2023, 2024). Finally, vocal plasticity during infancy has also been described for other primate species (e.g., Gouzoules & Gouzoules, 1990; Hauser, 1989; Koda et al., 2013; Omedes, 1985; Seyfarth & Cheney, 1986).
L.416-431: Even though I agree with you that the fact that whimpers do not encode population identity makes it more likely that genetics cannot be the sole explanation for the variability in calls, you should still mention that an alternative possibility exists: Since whimpers encode distress, it cannot be excluded that individuals which, due to genetic variation, produce altered calls may receive less maternal attention and thus have reduced chances of survival, ultimately causing this phenotype to disappear before it can become established. This possibility should be mentioned.*

**Author response 21:** We thank the reviewer for this helpful suggestion. We have substantially revised this section of the Discussion in lines 415-466 to place our findings within the broader context of vocal plasticity in non-human primates. Specifically, we now discuss evidence for socially guided vocal development in common marmosets, reports of vocal plasticity during ontogeny in several other primate species, and population-level vocal variation and vocal learning in mouse lemurs. We also retain the comparison with humans, as we believe it provides a useful parallel for the observed call-type differences in acoustic variability.

We also agree that the absence of population differences in whimpers does not completely exclude a genetic contribution. We have therefore added a paragraph acknowledging the alternative possibility that distress calls may be subject to stronger stabilising selection because altered acoustic structure could reduce maternal responsiveness and ultimately offspring survival. We now present this as an additional, non-mutually exclusive explanation for the observed conservation of whimpers.
