# Supplementary material for: Ontogeny of dialects across chimpanzee vocalisations
Source: PLoS One. 2026 Sep 2;21(9):e0355313. doi: 10.1371/journal.pone.0355313 (PMC13537595; doi:10.1371/journal.pone.0355313)
Supplement: Peer review history

# Peer review: Decision Letter 0

**DOI:** 10.1371/journal.pone.0355313.r001

**Authors:** Catalina Villamil

23 Dec 2025

Dear Dr. Giragosian,

Please submit your revised manuscript by Feb 06 2026 11:59PM. If you will need more time than this to complete your revisions, please reply to this message or contact the journal office at plosone@plos.org. When you're ready to submit your revision, log on to https://www.editorialmanager.com/pone/ and select the 'Submissions Needing Revision' folder to locate your manuscript file.

- A letter that responds to each point raised by the academic editor and reviewer(s). You should upload this letter as a separate file labeled 'Response to Reviewers'.
- A marked-up copy of your manuscript that highlights changes made to the original version. You should upload this as a separate file labeled 'Revised Manuscript with Track Changes'.
- An unmarked version of your revised paper without tracked changes. You should upload this as a separate file labeled 'Manuscript'.

If you would like to make changes to your financial disclosure, please include your updated statement in your cover letter. Guidelines for resubmitting your figure files are available below the reviewer comments at the end of this letter.

If applicable, we recommend that you deposit your laboratory protocols in protocols.io to enhance the reproducibility of your results. Protocols.io assigns your protocol its own identifier (DOI) so that it can be cited independently in the future. For instructions see: https://journals.plos.org/plosone/s/submission-guidelines#loc-laboratory-protocols. Additionally, PLOS ONE offers an option for publishing peer-reviewed Lab Protocol articles, which describe protocols hosted on protocols.io. Read more information on sharing protocols at https://plos.org/protocols?utm_medium=editorial-email&utm_source=authorletters&utm_campaign=protocols.

We look forward to receiving your revised manuscript.

Kind regards,

Catalina I Villamil

Academic Editor

PLOS One

Journal Requirements:

When submitting your revision, we need you to address these additional requirements.

1.Please ensure that your manuscript meets PLOS ONE's style requirements, including those for file naming. The PLOS ONE style templates can be found at

https://journals.plos.org/plosone/s/file?id=wjVg/PLOSOne_formatting_sample_main_body.pdf and

https://journals.plos.org/plosone/s/file?id=ba62/PLOSOne_formatting_sample_title_authors_affiliations.pdf

2. Please include a complete copy of PLOS’ questionnaire on inclusivity in global research in your revised manuscript. Our policy for research in this area aims to improve transparency in the reporting of research performed outside of researchers’ own country or community. The policy applies to researchers who have travelled to a different country to conduct research, research with Indigenous populations or their lands, and research on cultural artefacts. The questionnaire can also be requested at the journal’s discretion for any other submissions, even if these conditions are not met.  Please find more information on the policy and a link to download a blank copy of the questionnaire here: https://journals.plos.org/plosone/s/best-practices-in-research-reporting. Please upload a completed version of your questionnaire as Supporting Information when you resubmit your manuscript.

3. We note that your Data Availability Statement is currently as follows: All relevant data are within the manuscript and in Supporting Information files.

Please confirm at this time whether or not your submission contains all raw data required to replicate the results of your study. Authors must share the “minimal data set” for their submission. PLOS defines the minimal data set to consist of the data required to replicate all study findings reported in the article, as well as related metadata and methods (https://journals.plos.org/plosone/s/data-availability#loc-minimal-data-set-definition).

For example, authors should submit the following data:

- The values behind the means, standard deviations and other measures reported;

- The values used to build graphs;

- The points extracted from images for analysis.

Authors do not need to submit their entire data set if only a portion of the data was used in the reported study.

If your submission does not contain these data, please either upload them as Supporting Information files or deposit them to a stable, public repository and provide us with the relevant URLs, DOIs, or accession numbers. For a list of recommended repositories, please see https://journals.plos.org/plosone/s/recommended-repositories.

If there are ethical or legal restrictions on sharing a de-identified data set, please explain them in detail (e.g., data contain potentially sensitive information, data are owned by a third-party organization, etc.) and who has imposed them (e.g., an ethics committee). Please also provide contact information for a data access committee, ethics committee, or other institutional body to which data requests may be sent. If data are owned by a third party, please indicate how others may request data access.

4. We notice that your supplementary tables are included in the manuscript file. Please remove them and upload them with the file type 'Supporting Information'. Please ensure that each Supporting Information file has a legend listed in the manuscript after the references list.

5. We note that there is identifying data in the Supporting Information Table 1 and Supporting Information file “Acoustic Data Analysis.xlsx”. Due to the inclusion of these potentially identifying data, we have removed this file from your file inventory. Prior to sharing human research participant data, authors should consult with an ethics committee to ensure data are shared in accordance with participant consent and all applicable local laws.

Data sharing should never compromise participant privacy. It is therefore not appropriate to publicly share personally identifiable data on human research participants. The following are examples of data that should not be shared:

-Name, initials, physical address

-Ages more specific than whole numbers

-Internet protocol (IP) address

-Specific dates (birth dates, death dates, examination dates, etc.)

-Contact information such as phone number or email address

-Location data

-ID numbers that seem specific (long numbers, include initials, titled “Hospital ID”) rather than random (small numbers in numerical order)

Data that are not directly identifying may also be inappropriate to share, as in combination they can become identifying. For example, data collected from a small group of participants, vulnerable populations, or private groups should not be shared if they involve indirect identifiers (such as sex, ethnicity, location, etc.) that may risk the identification of study participants.

Additional guidance on preparing raw data for publication can be found in our Data Policy (https://journals.plos.org/plosone/s/data-availability#loc-human-research-participant-data-and-other-sensitive-data) and in the following article: http://www.bmj.com/content/340/bmj.c181.long.

Please remove or anonymize all personal information (<specific identifying information in file to be removed>), ensure that the data shared are in accordance with participant consent, and re-upload a fully anonymized data set. Please note that spreadsheet columns with personal information must be removed and not hidden as all hidden columns will appear in the published file.

6. If the reviewer comments include a recommendation to cite specific previously published works, please review and evaluate these publications to determine whether they are relevant and should be cited. There is no requirement to cite these works unless the editor has indicated otherwise.

Additional Editor Comments:

The reviewer notes several issues that should be addressed. This includes aspects of language (accent versus dialect), as well as several statistical issues that should be addressed. I would also note that the breakdown of the number of grunts, laughs, and whimpers by site should include a breakdown of the number by age category. There should also be a clearer indication of the number of repeat measurements to better evaluate the results (e.g. the number of individuals represented within each breakdown).

Reviewers' comments:

Reviewer's Responses to Questions

**Comments to the Author**

1. Is the manuscript technically sound, and do the data support the conclusions?

Reviewer #1: Yes

**********

2. Has the statistical analysis been performed appropriately and rigorously?

Reviewer #1: Yes

**********

3. Have the authors made all data underlying the findings in their manuscript fully available?

The PLOS Data policy

Reviewer #1: No

**********

4. Is the manuscript presented in an intelligible fashion and written in standard English?

Reviewer #1: Yes

**********

Reviewer #1: Comments:

The manuscript by Giragosian et al. analyzes infant and juvenile vocalizations of chimpanzees from two sites to test whether population-specific signatures exist and whether these signatures are stronger in juveniles than in infants. To address this question, the authors use a large dataset (N = 11225 calls) comprising three call types (grunts, whimpers, and laughs), from which they extract acoustic parameters and apply permuted discriminant function analyses to test for site-level differentiation. They report that the two sites can be discriminated for all call types, and that classification accuracy increases from infancy to the juvenile stage.

The dataset is rich, and this manuscript addresses a question of central importance for understanding chimpanzee vocal development and for informing broader debates on the evolution of vocal learning in both non-human primates and humans. However, several analytical decisions require clearer justification to assess the robustness of the findings, and additional contextualization would strengthen the interpretation. I recommend that the authors revisit their analytical decisions and justifications, and consider adding clearer explanations as well as complementary analyses or visualizations to make the underlying data patterns more transparent and the strength of the results easier for readers to evaluate.

Major comments

- The authors use the term “accents” and, in lines 44–45, distinguish accents from dialects in the context of human speech. However, in the non-human vocal-learning literature, dialect is far more commonly used to describe population-level variation in vocal production (e.g., Janik & Slater 1997; Nowicki & Searcy 2014; Desai et al. 2022). Therefore, it is not clear why the authors are using accents rather than dialects in the context of non-human primates. I believe the use of this term requires further justification and would greatly benefit from references to other studies on vocal convergence of non-human primates (often described as vocal accommodation) and/or other vocal-learning species in which the term “accent” has been used instead of “dialect”.

- Line 237: Why is a chi-squared test on call-level “correct vs. incorrect” classifications appropriate? Because multiple calls come from the same individuals, the observations are not independent, which violates a key assumption of the chi-squared test and may lead to underestimated uncertainty and inflated significance. For this reason, I am not sure that a chi-squared test is the most appropriate approach here, and I would encourage either a method that accounts for repeated measures or a more detailed justification of why the chi-squared test remains valid despite this non-independence.

- Why was age treated categorically rather than continuously? Given the range of ages and the fact that some individuals were recorded at multiple ages, modelling age as a continuous predictor could avoid arbitrary developmental boundaries and provide a more nuanced view of ontogenetic change. Could the authors clarify why this option was not pursued, and whether continuous-age analyses might be feasible with this dataset to offer additional insight?

- Why was the contribution of specific acoustic features to the site discrimination not explored? Reporting the underlying DFA loadings or structure coefficients could provide useful insight into the acoustic basis of the differences, even if these results need to be interpreted cautiously due to the sample-size imbalance (or addressed through resampling to reduce this imbalance).

- The interpretation of developmental changes should acknowledge other factors that may contribute to the observed patterns, including anatomical maturation and age-related differences in recording context (e.g., infants being held by their mothers).

- A brief discussion of the social contexts in which juveniles produce these calls would strengthen the interpretation. Do juveniles converge primarily with peers, adults, or through general exposure? Even speculative discussion of potential mechanisms (e.g., social feedback vs. eavesdropping) would be valuable.

- The two sites differ substantially in their environmental characteristics. Are the acoustic differences reported consistent with predictions based on habitat structure (e.g., dry vs. wet, open vs. dense environments) and known effects on sound transmission? The interpretation would benefit from a discussion of how environmental conditions might shape the specific acoustic parameters analyzed and whether the observed site differences align with these expectations.

Minor comments

- In line 233, please clarify what is meant by “restricting by developmental stage”.

- It is not clear why the specific spectral and temporal features listed in Table 2 were chosen. For example, Desai et al. (2022) justified their selection by choosing acoustic features that were as similar as possible to those used in previous studies of adult chimpanzee dialects (Mitani et al., 1992, 1999; Crockford et al., 2004).

- Line 75: The authors note that a recent study found that apparent population-specific vocal signatures could instead be explained by individual identity cues. Could the authors clarify how their analyses account for this possibility? For instance, what steps were taken to ensure that site-level differences are not confounded with individual-level vocal signatures?

- Please explain and justify the choices in reference to previous analyses.

- Does the production frequency of the three call types (grunts, whimpers, laughs) change across development?

- The authors briefly mention that the infant category spans from 6 months to 4 years of age, which may provide enough time for vocal learning to occur. It would be helpful to provide some reference to evaluate what is “enough time” for vocal learning.

- Could the analytical framework used here—in principle—be applied to human accents? How do the acoustic features most important for distinguishing human accents compare to those analyzed in chimpanzees?

- The analysis code, as well as the original data points behind the means and variance measures, should be made available for reproducibility.

**********

what does this mean?). If published, this will include your full peer review and any attached files.

If you choose “no”, your identity will remain anonymous but your review may still be made public.

**Do you want your identity to be public for this peer review?**  For information about this choice, including consent withdrawal, please see our Privacy Policy

Reviewer #1: No

**********

[NOTE: If reviewer comments were submitted as an attachment file, they will be attached to this email and accessible via the submission site. Please log into your account, locate the manuscript record, and check for the action link "View Attachments". If this link does not appear, there are no attachment files.]

To ensure your figures meet our technical requirements, please review our figure guidelines: https://journals.plos.org/plosone/s/figures

You may also use PLOS’s free figure tool, NAAS, to help you prepare publication quality figures: https://journals.plos.org/plosone/s/figures#loc-tools-for-figure-preparation.

NAAS will assess whether your figures meet our technical requirements by comparing each figure against our figure specifications.

# Author comment: Author response to Decision Letter 1

**DOI:** 10.1371/journal.pone.0355313.r002

2 Mar 2026

Ontogeny of dialects across chimpanzee vocalizations

We would like to thank all the Reviewers and the Editor for their useful comments, which greatly helped us to improve our previous manuscript. Below, we discuss the Reviewers comments in detail. For convenience, we reproduce the Reviewers’ comments in italics (marked by “Reviewer comment x:”), followed by our response (marked by “Author response:”). We numbered Reviewers’ comments continuously in order to facilitate referring back to previous Reviewer comments and Author responses (points).

Reviewer #1

Reviewer comment 1: The manuscript by Giragosian et al. analyzes infant and juvenile vocalizations of chimpanzees from two sites to test whether population-specific signatures exist and whether these signatures are stronger in juveniles than in infants. To address this question, the authors use a large dataset (N = 11225 calls) comprising three call types (grunts, whimpers, and laughs), from which they extract acoustic parameters and apply permuted discriminant function analyses to test for site-level differentiation. They report that the two sites can be discriminated for all call types, and that classification accuracy increases from infancy to the juvenile stage.

The dataset is rich, and this manuscript addresses a question of central importance for understanding chimpanzee vocal development and for informing broader debates on the evolution of vocal learning in both non-human primates and humans. However, several analytical decisions require clearer justification to assess the robustness of the findings, and additional contextualization would strengthen the interpretation. I recommend that the authors revisit their analytical decisions and justifications, and consider adding clearer explanations as well as complementary analyses or visualizations to make the underlying data patterns more transparent and the strength of the results easier for readers to evaluate.

Author response: We thank the reviewer for their positive and constructive assessment of the manuscript and for highlighting the relevance of this work for understanding chimpanzee vocal development and vocal learning. We are grateful for their careful evaluation of our analytical approach and for their suggestions to improve transparency and interpretability.

In response, we have substantially revised the analyses and presentation. Most notably, we have adopted a new analytical framework to assess developmental changes in population-level discrimination, using generalized linear mixed models of correct classification rates rather than chi-square tests. This approach allows a more robust treatment of developmental trajectories while accounting for repeated measures and individual-level variation.

In addition, we now explicitly incorporate and interpret factor loadings from the discriminant function analyses. This enables us to identify which acoustic parameters drive population differentiation and to link these differences more directly to biologically and ecologically meaningful processes, rather than relying solely on classification accuracy.

We believe these changes significantly strengthen the robustness of the analyses and provide a clearer, more mechanistic interpretation of why and how populations differ across development. We thank the reviewer for comments that directly motivated these improvements.

Reviewer comment 2: The authors use the term "accents" and, in lines 44–45, distinguish accents from dialects in the context of human speech. However, in the non-human vocal-learning literature, dialect is far more commonly used to describe population-level variation in vocal production (e.g., Janik & Slater 1997; Nowicki & Searcy 2014; Desai et al. 2022). Therefore, it is not clear why the authors are using accents rather than dialects in the context of non-human primates. I believe the use of this term requires further justification and would greatly benefit from references to other studies on vocal convergence of non-human primates (often described as vocal accommodation) and/or other vocal-learning species in which the term "accent" has been used instead of "dialect".

Author response: We thank the reviewer for highlighting this issue. Our decision to use the term accent over dialect was that as we understood, in humans, dialects are cases in which different words are used to communicate the same things, whereas accents are cases in which the same word in pronounced differently. Since we were looking at how the same call is constructed acoustically in different age groups and populations, we opted for the latter terminology. However, we acknowledge that this is not the conventional use of the term in the animal communication literature, as demonstrated by the provided references. Since addressing the conceptual inconsistencies in the use of the terms accent and dialect in the communication literature more generally is beyond the scope of our paper, we have decided to change our use of the term ‘accent’ and instead have used the term ‘dialect’.

Reviewer comment 3: Line 237: Why is a chi-squared test on call-level "correct vs. incorrect" classifications appropriate? Because multiple calls come from the same individuals, the observations are not independent, which violates a key assumption of the chi-squared test and may lead to underestimated uncertainty and inflated significance. For this reason, I am not sure that a chi-squared test is the most appropriate approach here, and I would encourage either a method that accounts for repeated measures or a more detailed justification of why the chi-squared test remains valid despite this non-independence.

Author response: We thank the reviewer for raising this important concern regarding non-independence of observations. We agree that a simple chi-squared test on call-level classifications does not adequately account for repeated measures and could lead to underestimated uncertainty.

In response, we have revised our analytical approach. First, the discriminant function analyses were conducted using a permuted DFA framework in which individual identity was controlled by restricting permutations within individuals, thereby preventing inflation of classification accuracy due to repeated sampling of the same callers.

Secondly, we have now replaced the chi-squared tests with beta regression mixed models to examine developmental differences in classification accuracy. These models treat classification rate as a proportional response variable and include bout ID as a random intercept, thereby directly accounting for repeated measures within bouts. This approach provides a more statistically appropriate framework for assessing age-related differences in discrimination accuracy.

We believe this revised analysis addresses the reviewer’s concern and substantially strengthens the robustness of our results.

Reviewer comment 4: Why was age treated categorically rather than continuously? Given the range of ages and the fact that some individuals were recorded at multiple ages, modelling age as a continuous predictor could avoid arbitrary developmental boundaries and provide a more nuanced view of ontogenetic change. Could the authors clarify why this option was not pursued, and whether continuous-age analyses might be feasible with this dataset to offer additional insight?

Author response: We thank the reviewer for raising this point. We agree that modelling age continuously would be interesting and informative. Unfortunately, however, there is insufficient data to run a pDFA model at each age (i.e., there are not individuals from all ages in each group, see supplementary table S1). Since our pDFA models have been performed at the age class level, and the aim of our developmental analysis is to understand the relationship between our pDFA models (i.e., to see whether there are differences in correct classification rates), in combination with limited data at certain ages, we have decided to use age class as a categorical variable in our mixed-models of classification rates also. We have clarified this in our methods section, and we feel it is a reasonable approach, given that chimpanzee life-history can be meaningfully divided into infant and juvenile periods (which are behaviourally, anatomically, and physiologically distinct periods in the life span), which are commonly used in many studies of chimpanzee ontogeny.

Reviewer comment 5: Why was the contribution of specific acoustic features to the site discrimination not explored? Reporting the underlying DFA loadings or structure coefficients could provide useful insight into the acoustic basis of the differences, even if these results need to be interpreted cautiously due to the sample-size imbalance (or addressed through resampling to reduce this imbalance).

Author response: We thank the reviewer for this valuable suggestion. We agree that examining the contribution of specific acoustic parameters to site discrimination provides important insight into the acoustic basis of the observed differences.

In response, we now report the factor loadings from all discriminant function analyses in the Results section and provide full loading summaries (including means, standard deviations, confidence intervals, and sign consistency across permutations) in the Supplementary Materials for all models. Because our DFA framework was implemented using a permutation procedure that controls for individual identity, we summarise loadings across permutations to provide a robust estimate of each parameter’s contribution while accounting for sample-size imbalance and repeated measures.

We have also revised the Discussion to explicitly interpret which acoustic domains (e.g., power density, temporal patterning, entropy measures) drive site-level discrimination and how their contributions change across development.

We believe these additions substantially clarify the acoustic basis of population differentiation and strengthen the transparency and interpretability of our findings.

Reviewer comment 6: The interpretation of developmental changes should acknowledge other factors that may contribute to the observed patterns, including anatomical maturation and age-related differences in recording context (e.g., infants being held by their mothers).

Author response: We thank the reviewer for highlighting additional factors that may contribute to the observed developmental changes in classification accuracy. We agree that anatomical maturation and age-related differences in recording context are important considerations.

In the revised Discussion, we now explicitly acknowledge the potential role of vocal tract growth and improving motor control in shaping call structure across development. We note that anatomical maturation may increase the stability and modulation capacity of vocalizations, potentially amplifying population-level differences that are only weakly expressed in infancy.

We also address the possibility that age-related differences in behavioural or recording context (e.g., infants being carried by their mothers or vocalizing at closer range) could influence acoustic properties or transmission characteristics. While our analyses focus on structural acoustic parameters rather than raw amplitude measures, and thus are unlikely to be solely explained by recording distance, we acknowledge that contextual factors may interact with developmental changes in vocal production.

These additions broaden the interpretation of our findings and clarify that ecological calibration, anatomical maturation, and socially mediated processes are not mutually exclusive explanations for the observed developmental strengthening of population signatures.

Reviewer comment 7: A brief discussion of the social contexts in which juveniles produce these calls would strengthen the interpretation. Do juveniles converge primarily with peers, adults, or through general exposure? Even speculative discussion of potential mechanisms (e.g., social feedback vs. eavesdropping) would be valuable.

Author response: We thank the reviewer for this helpful suggestion. In the revised Discussion, we now include a brief consideration of the social contexts in which juveniles produce each call type and how these may contribute to developmental strengthening of population-level vocal signatures.

Specifically, we note that laughs are primarily produced during play and are most often directed toward peers, potentially providing opportunities for convergence through repeated social interaction and feedback within age cohorts. In contrast, whimpers are predominantly directed toward mothers, which may limit broader social shaping opportunities. Grunts show a developmental shift in social targeting: although infants grunt toward a range of partners, juveniles increasingly direct grunts toward higher-ranking individuals, similar to adults. Such changes in interaction partners may alter the available models for vocal exposure and influence the emergence of population-specific structure.

We also briefly outline potential mechanisms, including social feedback and passive exposure (eavesdropping), while acknowledging that our current dataset does not allow us to disentangle these processes. We believe this addition strengthens the interpretation of our developmental findings and clarifies the potential social pathways through which population signatures may emerge.

Reviewer comment 8: The two sites differ substantially in their environmental characteristics. Are the acoustic differences reported consistent with predictions based on habitat structure (e.g., dry vs. wet, open vs. dense environments) and known effects on sound transmission? The interpretation would benefit from a discussion of how environmental conditions might shape the specific acoustic parameters analyzed and whether the observed site differences align with these expectations.

Author response: We thank the reviewer for this insightful comment. In the revised Discussion, we now more explicitly situate the observed acoustic differences within predictions derived from habitat structure and known effects of environmental conditions on sound transmission.

Specifically, we highlight that the population inhabiting dense tropical rainforest (Gombe) consistently exhibited higher average power density than the population living in the more open miombo woodland (Chimfunshi). Increased signal energy is consistent with expectations for structurally complex habitats, where dense vegetation increases attenuation and scattering of sound and may favour signals with greater energy to enhance transmission.

In addition, we now expand our interpretation of temporal parameters that contributed to site discrimination, including element rate and total number of elements per bout. We discuss the possibility that increased repetition within call bouts may introduce redundancy, thereby enhancing detectability and robustness to degradation in acoustically challenging environments. Repeated elements can increase the likelihood that at least part of a signal is received clearly despite environmental masking and may allow receivers to integrate information across multiple acoustic events. Thus, habitat-related constraints may act not only on spectral energy (e.g., power density) but also on the temporal organization of calls.

We also clarify that, while the direction of the power density differences aligns with ecological predictions, not all acoustic parameters map straightforwardly onto habitat-based expectations, and multiple processes — including ecological calibration, anatomical maturation, and socially mediated influences — may interact to produce the observed population signatures. We believe these revisions strengthen the ecological framing of our findings while maintaining appropriate caution in interpretation.

Reviewer comment 9: In line 233, please clarify what is meant by "restricting by developmental stage".

Author response: We have now clarified that this means a separate model was done for each age class for each call type.

Reviewer comment 10: It is not clear why the specific spectral and temporal features listed in Table 2 were chosen. For example, Desai et al. (2022) justified their selection by choosing acoustic features that wer

Attachment Submitted filename: Response to Reviewers.docx

# Peer review: Decision Letter 1

**DOI:** 10.1371/journal.pone.0355313.r003

**Authors:** Catalina Villamil

6 Apr 2026

Dear Dr. Giragosian,

Thank you for submitting your manuscript to PLOS ONE. After careful consideration, we feel that it has merit but does not fully meet PLOS ONE’s publication criteria as it currently stands. Therefore, we invite you to submit a revised version of the manuscript that addresses the points raised during the review process.

==============================

Thank you for your resubmission of this interesting manuscript. Two reviewers have evaluated the manuscript and find that it is much improved. One reviewer requests minor revisions to address a specific methodological question, while the other reviewer requests a variety of small revisions, primarily in terms of clarity and explanation in the text. I generally concur with the findings of the two reviewers. Please see their detailed comments below.

==============================

Please submit your revised manuscript by May 21 2026 11:59PM. If you will need more time than this to complete your revisions, please reply to this message or contact the journal office at plosone@plos.org. When you're ready to submit your revision, log on to https://www.editorialmanager.com/pone/ and select the 'Submissions Needing Revision' folder to locate your manuscript file.

- A letter that responds to each point raised by the academic editor and reviewer(s). You should upload this letter as a separate file labeled 'Response to Reviewers'.
- A marked-up copy of your manuscript that highlights changes made to the original version. You should upload this as a separate file labeled 'Revised Manuscript with Track Changes'.
- An unmarked version of your revised paper without tracked changes. You should upload this as a separate file labeled 'Manuscript'.

If you would like to make changes to your financial disclosure, please include your updated statement in your cover letter. Guidelines for resubmitting your figure files are available below the reviewer comments at the end of this letter.

If applicable, we recommend that you deposit your laboratory protocols in protocols.io to enhance the reproducibility of your results. Protocols.io assigns your protocol its own identifier (DOI) so that it can be cited independently in the future. For instructions see: https://journals.plos.org/plosone/s/submission-guidelines#loc-laboratory-protocols. Additionally, PLOS ONE offers an option for publishing peer-reviewed Lab Protocol articles, which describe protocols hosted on protocols.io. Read more information on sharing protocols at https://plos.org/protocols?utm_medium=editorial-email&utm_source=authorletters&utm_campaign=protocols.

As the corresponding author, your ORCID iD is verified in the submission system and will appear in the published article. PLOS supports the use of ORCID, and we encourage all coauthors to register for an ORCID iD and use it as well. Please encourage your coauthors to verify their ORCID iD within the submission system before final acceptance, as unverified ORCID iDs will not appear in the published article. *Only*  the individual author can complete the verification step; PLOS staff *cannot*  verify ORCID iDs on behalf of authors.

We look forward to receiving your revised manuscript.

Kind regards,

Catalina I Villamil

Academic Editor

PLOS One

Journal Requirements:

1. If the reviewer comments include a recommendation to cite specific previously published works, please review and evaluate these publications to determine whether they are relevant and should be cited. There is no requirement to cite these works unless the editor has indicated otherwise.

2. Please review your reference list to ensure that it is complete and correct. If you have cited papers that have been retracted, please include the rationale for doing so in the manuscript text, or remove these references and replace them with relevant current references. Any changes to the reference list should be mentioned in the rebuttal letter that accompanies your revised manuscript. If you need to cite a retracted article, indicate the article’s retracted status in the References list and also include a citation and full reference for the retraction notice.

[Note: HTML markup is below. Please do not edit.]

Reviewer's Responses to Questions

**Comments to the Author**

Reviewer #1: (No Response)

Reviewer #2: (No Response)

**********

2. Is the manuscript technically sound, and do the data support the conclusions?

Reviewer #1: Yes

Reviewer #2: Yes

**********

3. Has the statistical analysis been performed appropriately and rigorously?

Reviewer #1: Yes

Reviewer #2: Yes

**********

4. Have the authors made all data underlying the findings in their manuscript fully available?

The PLOS Data policy

Reviewer #1: Yes

Reviewer #2: Yes

**********

5. Is the manuscript presented in an intelligible fashion and written in standard English?

Reviewer #1: Yes

Reviewer #2: Yes

**********

Reviewer #1: The authors did a good job addressing all my comments, and this new version of the manuscript is significantly more robust and informative regarding their methods and result interpretations.

I still have two comments about clarifications that should be made about their analysis:

1) Accounting for individual identity in the infancy/juvenile discrimination:

In lines 244-250, the authors describe the model they used to test population-level classification accuracy between age groups.

They accounted for the non-independence of calls produced within the same vocal bout, but there is nothing about individual identity. Calls can have correlated classification rates both by being in the same bout and by being from the same individual. The current model does not account for this, but it could be easily included by adding individual identity as a random intercept in the model.

Additionally, for clarity, I believe the model equation should be stated in the methods section or the supporting material; it took me a while to find it in the script (class_rate_beta ~ age_group + (1 | id_bout)).

2) The authors should make it clear in the methods section that group coding was held constant across all 100 iterations, as this is a requirement to use the sign consistency metric. Additionally, please include an explicit statement of which population is the positive reference in the factor loading analysis.

Reviewer #2: Giragosian et al. Investigate the existence of dialects across two distinct chimpanzee populations and across different age classes, with the ultimate goal of providing insight into the ontogeny of accent development in the species. In general, I found the manuscript well-written, the methods used appropriate, the findings very interesting and mostly well discussed. However, there are some minor points that I think should be addressed before the manuscript is ready for publication.

L.32, L.74, L.399: you should use furthermore instead of further.

L .58-59: in the brackets use e.g., as you do not cover the entirety of nonhuman primates for which cross-population variation has been shown. In addition, you are missing the closing bracket in the sentence.

L.65: Use Nonetheless instead of Although.

L72-74: add references in the end of the sentence, to support your statement.

L.118: it should be and/or

L.129: as you have 30 subjects, but one animal appears twice in your dataset because it represented in both ages classes (resulting in N=31), I would suggest writing “30 individual chimpanzees”. In addition, I would add in brackets how many animals in total were from Chimfushi Wildlife Orphanage and how many from Gombe National Park in L.129-130.

L.134-135: I think you must have made a mistake in the calculation of the Mean. With the ages you have in S1 Table and with the span of the ages being between 4 and 10 you should not have a mean of 10.90.

L.137-140: as you classified the animals in juvenile or infant based solely on age, I would remove the characteristics you list here, as they are not relevant for your study.

L.145, L.159: you should add information concerning the frequency sensitivity of the microphones and with which sampling frequency you conducted your recordings.

L.172-177: the coded unit in your study were not call types in general, but only the call types grunt, whimper and laughs. Therefore, I would suggest removing the first sentences and directly say that you only focused on the three call types whose definitions you provide, as they are the most predominant ones in infants and juveniles.

Table 1: the references should be cited not only with the names of the authors and years of publication, but also be numbered, so one can directly find them in the references section of the publication.

L.186: 5 seconds after the offset or the onset of the previous call? Please add this information in the sentence.

L.194-195: were the calls recorded with this sampling rate or were they transformed before the analysis?

L.205: I find the threshold of 0.9 too high to reduce collinearity issues. Usually more conservative thresholds of 0.7 or 0.8 are used. How high were the correlations between your acoustic parameters and how can you justify the use of such a high threshold? Please add the correlations you calculated in the supplementary and justify in your manuscript the threshold you used.

L.206-207: how many acoustic parameters did you measure in total, how many and which ones did you remove due to collinearity issues and why did you choose the ones you chose? Please add this information in your manuscript.

L.222: did you use the same number of calls from each population to have ~50% chance? The fact that you have two populations is not on its own sufficient to result in a 50% chance.

L.235: you have a small typo, it should be our and not out.

L.251: a general remark about the Results section. I find your results in general nicely presented, but I find that you could use nicer introductory sentences throughout the results, to make the section easier to read and comprehend. For example, in L260 instead of “Population discrimination was significant” you could have something like “For infant grunts population discrimination was significant” or “For infant grunts there were significant differences between the two populations” or something like that. That is something that I would suggest doing for the introductory sentence of every single call type.

L.390-395: this sentence is too long. I would suggest diving it in two sentences.

L.392: you should add a reference for laughs

L.402-415: I do not agree that the only comparison you bring into the discussion is with humans. There is extensive literature on many other nonhuman primates that you should integrate. For example, there is substantial work on vocal learning in marmosets during infancy that fully supports your arguments (e.g., Ghazanfar et al., 2026; Gultekin & Hage, 2017, 2018; Takahashi et al., 2017). In addition, differences in the vocalisations of different populations of primates have been described in mouse lemurs (e.g., Hafen et al.,1998), for which also vocal learning during adulthood and vocal plasticity during ontogeny have been suggested (Langehennig-Peristenidou et al. 2023, 2024). Finally, vocal plasticity during infancy has also been described for other primate species (e.g., Gouzoules & Gouzoules, 1990; Hauser, 1989; Koda et al., 2013; Omedes, 1985; Seyfarth & Cheney, 1986).

L.416-431: Even though I agree with you that the fact that whimpers do not encode population identity makes it more likely that genetics cannot be the sole explanation for the variability in calls, you should still mention that an alternative possibility exists: Since whimpers encode distress, it cannot be excluded that individuals which, due to genetic variation, produce altered calls may receive less maternal attention and thus have reduced chances of survival, ultimately causing this phenotype to disappear before it can become established. This possibility should be mentioned.

**********

what does this mean?). If published, this will include your full peer review and any attached files.

If you choose “no”, your identity will remain anonymous but your review may still be made public.

**Do you want your identity to be public for this peer review?**  For information about this choice, including consent withdrawal, please see our Privacy Policy

Reviewer #1: No

Reviewer #2: No

**********

[NOTE: If reviewer comments were submitted as an attachment file, they will be attached to this email and accessible via the submission site. Please log into your account, locate the manuscript record, and check for the action link "View Attachments". If this link does not appear, there are no attachment files.]

To ensure your figures meet our technical requirements, please review our figure guidelines: https://journals.plos.org/plosone/s/figures

You may also use PLOS’s free figure tool, NAAS, to help you prepare publication quality figures: https://journals.plos.org/plosone/s/figures#loc-tools-for-figure-preparation.

NAAS will assess whether your figures meet our technical requirements by comparing each figure against our figure specifications.

# Author comment: Author response to Decision Letter 2

**DOI:** 10.1371/journal.pone.0355313.r004

2 Jul 2026

Ontogeny of dialects across chimpanzee vocalizations

We would like to thank all the Reviewers and the Editor for their useful comments, which greatly helped us to improve our previous manuscript. Below, we discuss the Reviewers comments in detail. For convenience, we reproduce the Reviewers’ comments in italics (marked by “Reviewer comment x:”), followed by our response (marked by “Author response:”). We numbered Reviewers’ comments continuously in order to facilitate referring back to previous Reviewer comments and Author responses (points).

Review Comments to the Author

Reviewer #1: The authors did a good job addressing all my comments, and this new version of the manuscript is significantly more robust and informative regarding their methods and result interpretations. I still have two comments about clarifications that should be made about their analysis:

Reviewer comment 1: Accounting for individual identity in the infancy/juvenile discrimination: In lines 244-250, the authors describe the model they used to test population-level classification accuracy between age groups. They accounted for the non-independence of calls produced within the same vocal bout, but there is nothing about individual identity. Calls can have correlated classification rates both by being in the same bout and by being from the same individual. The current model does not account for this, but it could be easily included by adding individual identity as a random intercept in the model. Additionally, for clarity, I believe the model equation should be stated in the methods section or the supporting material; it took me a while to find it in the script (class_rate_beta ~ age_group + (1 | id_bout)).

Author response 1: We agree with the reviewer that individual ID is an important source of variability that needs to be adequately controlled for. The pDFA package that we used allows you to control for grouping factors unrelated to your factor of interest. We have two relevant grouping factors: individual ID and bout ID. However, the software only allows you to control a single grouping factor. So, we decided to control for individual ID by holding it constant during the permutation process (as described in lines 231-235), and we controlled for bout ID by including this factor as a random intercept in our model. We have added an additional sentence in the data analysis section (lines 260-265) to clearly explain this. In addition, as the reviewer suggests, we have explicitly stated our model formula also.

Reviewer comment 2: The authors should make it clear in the methods section that group coding was held constant across all 100 iterations, as this is a requirement to use the sign consistency metric. Additionally, please include an explicit statement of which population is the positive reference in the factor loading analysis.

Author response 2: We thank the reviewer for pointing this out. We now clarify in lines 248-251 that we held the sign constant and specify which group was represented by + and which was represented by -.

Reviewer #2: Giragosian et al. Investigate the existence of dialects across two distinct chimpanzee populations and across different age classes, with the ultimate goal of providing insight into the ontogeny of accent development in the species. In general, I found the manuscript well-written, the methods used appropriate, the findings very interesting and mostly well discussed. However, there are some minor points that I think should be addressed before the manuscript is ready for publication.

Reviewer comment 1: L.32, L.74, L.399: you should use furthermore instead of further.

Author response: We thank the reviewer for the suggestion. We have made the edits suggested in lines 33, 77 and 401.

Reviewer comment 2: L .58-59: in the brackets use e.g., as you do not cover the entirety of nonhuman primates for which cross-population variation has been shown. In addition, you are missing the closing bracket in the sentence.

Author response: We thank the reviewer for the suggestion. We have made the edits suggested in lines 60-61.

Reviewer comment 3: L.65: Use Nonetheless instead of Although.

Author response: We thank the reviewer for the suggestion. We have made the edit suggested in line 65.

Reviewer comment 4: L72-74: add references in the end of the sentence, to support your statement.

Author response: We thank the reviewer for the suggestion. We have added references to support the claim in lines 75-76.

Reviewer comment 5: L.118: it should be and/or

Author response: We thank the reviewer for the suggestion. We have made the edit suggested in line 123.

Reviewer comment 6: L.129: as you have 30 subjects, but one animal appears twice in your dataset because it represented in both ages classes (resulting in N=31), I would suggest writing “30 individual chimpanzees”. In addition, I would add in brackets how many animals in total were from Chimfushi Wildlife Orphanage and how many from Gombe National Park in L.129-130.

Author response: We thank the reviewer for the suggestion. We have made the edits suggested in lines 134-135.

Reviewer comment 7: L.134-135: I think you must have made a mistake in the calculation of the Mean. With the ages you have in S1 Table and with the span of the ages being between 4 and 10 you should not have a mean of 10.90.

Author response: We thank the reviewer for the suggestion. We have made the edits suggested in lines 140.

Reviewer comment 8: L.137-140: as you classified the animals in juvenile or infant based solely on age, I would remove the characteristics you list here, as they are not relevant for your study.

Author response: We thank the reviewer for the suggestion. We have removed the characteristics described in lines 143.

Reviewer comment 9: L.145, L.159: you should add information concerning the frequency sensitivity of the microphones and with which sampling frequency you conducted your recordings.

Author response 10: We have added the sensitivity range for both devices, which was the same, in lines 148, 162-163 and 173-174. One set of recordings was analogue, so there is no sampling rate during recording, but we also now specify in the same section that both sets of recordings were digitised using the same sampling rate and bit-accuracy

Reviewer comment 10: L.172-177: the coded unit in your study were not call types in general, but only the call types grunt, whimper and laughs. Therefore, I would suggest removing the first sentences and directly say that you only focused on the three call types whose definitions you provide, as they are the most predominant ones in infants and juveniles.

Author response: We thank the reviewer for the suggestion. We have removed the first sentences of the paragraph as suggested, starting at line 179.

Reviewer comment 11: Table 1: the references should be cited not only with the names of the authors and years of publication, but also be numbered, so one can directly find them in the references section of the publication.

Author response: We thank the reviewer for the suggestion. We have numbered the citations in Table 1.

Reviewer comment 12: L.186: 5 seconds after the offset or the onset of the previous call? Please add this information in the sentence.

Author response: We thank the reviewer for the suggestion. We made the edit in line 187.

Reviewer comment 13: L.194-195: were the calls recorded with this sampling rate or were they transformed before the analysis?

Author response 13: This is the sampling rate set in Raven Pro after fast Fourier Transform of the signals, which we now specify in line 192.

Reviewer comment 14: L.205: I find the threshold of 0.9 too high to reduce collinearity issues. Usually more conservative thresholds of 0.7 or 0.8 are used. How high were the correlations between your acoustic parameters and how can you justify the use of such a high threshold? Please add the correlations you calculated in the supplementary and justify in your manuscript the threshold you used.

Author response 14: We thank the reviewer for this suggestion. We have now included the full pairwise correlation matrix in the Supplementary Material (Supplementary Figs. S1, S2, and S3). Pairwise Pearson correlation coefficients were calculated among all acoustic parameters, and highly correlated variables were identified using the findCorrelation() function in the R package caret, with a cutoff of r > 0.90. We chose this threshold to remove only variables that were nearly redundant while retaining descriptors that capture different aspects of call structure. Because many acoustic variables are inherently correlated, using a lower threshold (e.g. 0.7 or 0.8) would have resulted in the removal of variables that still provide biologically distinct information. We have clarified this rationale in the Methods in lines 205-218.

Reviewer comment 15: L.206-207: how many acoustic parameters did you measure in total, how many and which ones did you remove due to collinearity issues and why did you choose the ones you chose? Please add this information in your manuscript.

Author response 15: We have revised the Methods in lines 205-218 to specify the number of acoustic parameters included in the correlation analysis, the variables removed, and the procedure used for variable selection. Initially, 22 acoustic parameters were considered. Pairwise correlations were calculated among all variables, and variables with r > 0.90 were identified using findCorrelation() from the caret package. This function iteratively removes the variable with the largest mean absolute correlation among highly correlated pairs, thereby minimizing redundancy in the retained dataset. Following this procedure, 7 variables were removed (see supplementary figure S1, S2 and S3), leaving 15 variables for subsequent analyses.

Reviewer comment 16: L.222: did you use the same number of calls from each population to have ~50% chance? The fact that you have two populations is not on its own sufficient to result in a 50% chance.

Author response 16: Thank you for pointing this out. The expected classification is actually calculated across 1000 randomised permutations, and this gives the base rate to which the model performance is compared to, which is explained in the sentence that follows, and this part referring to a 50% chance has been removed in line 233.

Reviewer comment 17: L.235: you have a small typo, it should be our and not out.

Author response: We thank the reviewer for the suggestion. We have corrected the typo in line 245.

Reviewer comment 18: L.251: a general remark about the Results section. I find your results in general nicely presented, but I find that you could use nicer introductory sentences throughout the results, to make the section easier to read and comprehend. For example, in L260 instead of “Population discrimination was significant” you could have something like “For infant grunts population discrimination was significant” or “For infant grunts there were significant differences between the two populations” or something like that. That is something that I would suggest doing for the introductory sentence of every single call type.

Author response: We thank the reviewer for the suggestion. We have changed the introductory sentence for each call type in the results section as suggested throughout lines 268-384.

Reviewer comment 19: L.390-395: this sentence is too long. I would suggest diving it in two sentences.

Author response: We thank the reviewer for the suggestion. We have separated the sentence into two sentences in lines 389-394.

Reviewer comment 20: L.392: you should add a reference for laughs

Author response: We thank the reviewer for the suggestion. We have added a reference for laughs in line 391.

Reviewer comment 21: L.402-415: I do not agree that the only comparison you bring into the discussion is with humans. There is extensive literature on many other nonhuman primates that you should integrate. For example, there is substantial work on vocal learning in marmosets during infancy that fully supports your arguments (e.g., Ghazanfar et al., 2026; Gultekin & Hage, 2017, 2018; Takahashi et al., 2017). In addition, differences in the vocalisations of different populations of primates have been described in mouse lemurs (e.g., Hafen et al.,1998), for which also vocal learning during adulthood and vocal plasticity during ontogeny have been suggested (Langehennig-Peristenidou et al. 2023, 2024). Finally, vocal plasticity during infancy has also been described for other primate species (e.g., Gouzoules & Gouzoules, 1990; Hauser, 1989; Koda et al., 2013; Omedes, 1985; Seyfarth & Cheney, 1986).

L.416-431: Even though I agree with you that the fact that whimpers do not encode population identity makes it more likely that genetics cannot be the sole explanation for the variability in calls, you should still mention that an alternative possibility exists: Since whimpers encode distress, it cannot be excluded that individuals which, due to genetic variation, produce altered calls may receive less maternal attention and thus have reduced chances of survival, ultimately causing this phenotype to disappear before it can become established. This possibility should be mentioned.

Author response 21: We thank the reviewer for this helpful suggestion. We have substantially revised this section of the Discussion in lines 415-466 to place our findings within the broader context of vocal plasticity in non-human primates. Specifically, we now discuss evidence for socially guided vocal development in common marmosets, reports of vocal plasticity during ontogeny in several other primate species, and population-level vocal variation and vocal learning in mouse lemurs. We also retain the comparison with humans, as we believe it provides a useful parallel for the observed call-type differences in acoustic variability.

We also agree that the absence of population differences in whimpers does not completely exclude a genetic contribution. We have therefore added a paragraph acknowledging the alternative possibility that distress calls may be subject to stronger stabilising selection because altered acoustic structure could reduce maternal responsiveness and ultimately offspring survival. We now present this as an additional, non-mutually exclusive explanation for the observed conservation of whimpers.

Attachment Submitted filename: Response_to_Reviewers_auresp_2.docx

# Peer review: Decision Letter 2

**DOI:** 10.1371/journal.pone.0355313.r005

**Authors:** Catalina Villamil

21 Jul 2026

Ontogeny of dialects across chimpanzee vocalisations.

PONE-D-25-57421R2

Dear Dr. Giragosian,

We’re pleased to inform you that your manuscript has been judged scientifically suitable for publication and will be formally accepted for publication once it meets all outstanding technical requirements.

Within one week, you’ll receive an e-mail detailing the required amendments. When these have been addressed, you’ll receive a formal acceptance letter and your manuscript will be scheduled for publication.

An invoice will be generated when your article is formally accepted. Please note, if your institution has a publishing partnership with PLOS and your article meets the relevant criteria, all or part of your publication costs will be covered. Please make sure your user information is up-to-date by logging into Editorial Manager at Editorial Manager® and clicking the ‘Update My Information' link at the top of the page. For questions related to billing, please contact billing support.

If your institution or institutions have a press office, please notify them about your upcoming paper to help maximize its impact. If they’ll be preparing press materials, please inform our press team as soon as possible -- no later than 48 hours after receiving the formal acceptance. Your manuscript will remain under strict press embargo until 2 pm Eastern Time on the date of publication. For more information, please contact onepress@plos.org.

Kind regards,

Catalina I Villamil

Academic Editor

PLOS One

Additional Editor Comments (optional):

Thank you for revising your manuscript. Both reviewers agreed that the manuscript is much improved and has addressed their concerns.

Reviewers' comments:

Reviewer's Responses to Questions

**Comments to the Author**

Reviewer #1: All comments have been addressed

Reviewer #2: All comments have been addressed

**********

2. Is the manuscript technically sound, and do the data support the conclusions?

Reviewer #1: Yes

Reviewer #2: Yes

**********

3. Has the statistical analysis been performed appropriately and rigorously?

Reviewer #1: Yes

Reviewer #2: Yes

**********

4. Have the authors made all data underlying the findings in their manuscript fully available?

The PLOS Data policy

Reviewer #1: Yes

Reviewer #2: Yes

**********

5. Is the manuscript presented in an intelligible fashion and written in standard English?

Reviewer #1: Yes

Reviewer #2: Yes

**********

Reviewer #1: This version of the manuscript is significantly better.

Adding evidence for vocal plasticity during ontogeny for other nonhuman primates in the discussion is a key improvement in contextualizing the author’s findings.

The finding that "Classification accuracy was significantly higher in juveniles than in infants" could also be contextualized in terms of other nonhuman primates. Specifically, there are some hypotheses on how variation in both neural development and social environment might be related to variation in vocal plasticity, both within development and between species (Biazzi, Takahashi, and Ghazanfar. "Altricial brains and the evolution of infant vocal learning." (2025)).

My final suggestion is that placing your findings in light of these (or possibly other) hypotheses would improve the contextualization of your results within the literature on the evolution of vocal flexibility during ontogeny in primates.

Reviewer #2: The authors have adressed all comments that I made in an appropriate manner and in my opinion the manuscript is ready for publication.

**********

what does this mean?). If published, this will include your full peer review and any attached files.

If you choose “no”, your identity will remain anonymous but your review may still be made public.

**Do you want your identity to be public for this peer review?**  For information about this choice, including consent withdrawal, please see our Privacy Policy

Reviewer #1: No

Reviewer #2: No

**********

# Editor report: Acceptance letter

**DOI:** 10.1371/journal.pone.0355313.r006

**Authors:** Catalina Villamil

PONE-D-25-57421R2

PLOS One

Dear Dr. Giragosian,

I'm pleased to inform you that your manuscript has been deemed suitable for publication in PLOS One. Congratulations! Your manuscript is now being handed over to our production team.

At this stage, our production department will prepare your paper for publication. This includes ensuring the following:

* All references, tables, and figures are properly cited

* All relevant supporting information is included in the manuscript submission,

* There are no issues that prevent the paper from being properly typeset

You will receive further instructions from the production team, including instructions on how to review your proof when it is ready. Please keep in mind that we are working through a large volume of accepted articles, so please give us a few days to review your paper and let you know the next and final steps.

Lastly, if your institution or institutions have a press office, please let them know about your upcoming paper now to help maximize its impact. If they'll be preparing press materials, please inform our press team within the next 48 hours. Your manuscript will remain under strict press embargo until 2 pm Eastern Time on the date of publication. For more information, please contact onepress@plos.org.

You will receive an invoice from PLOS for your publication fee after your manuscript has reached the completed accept phase. If you receive an email requesting payment before acceptance or for any other service, this may be a phishing scheme. Learn how to identify phishing emails and protect your accounts at https://explore.plos.org/phishing.

If we can help with anything else, please email us at customercare@plos.org.

Thank you for submitting your work to PLOS ONE and supporting open access.

Kind regards,

PLOS ONE Editorial Office Staff

on behalf of

Dr. Catalina I Villamil

Academic Editor

PLOS One
